# Combination of Chromatographic and Machine Learning-Driven Virtual Fractionation Identifies Aryl Hydrocarbon Receptor Agonists in Sediments

**DOI:** 10.1021/acs.est.6c02059

**Published:** 2026-05-11

**Authors:** Haotian Wang, Georg Braun, Norbert Kamjunke, Martin Krauss, Guibin Jiang, Beate I. Escher

**Affiliations:** † Department of Cell Toxicology, Helmholtz Centre for Environmental Research−UFZ, Leipzig 04318, Germany; ‡ State Key Laboratory of Environmental Chemistry and Toxicology, Research Center for Eco-Environmental Sciences, 12381 Chinese Academy of Sciences, Beijing 100085, China; § Department of River Ecology, Helmholtz Centre for Environmental Research−UFZ, Magdeburg 39114, Germany; ∥ Department of Exposure Science, Helmholtz Centre for Environmental Research−UFZ, Leipzig 04318, Germany; ⊥ Environmental Toxicology, Department of Geosciences, Eberhard Karls University Tübingen, Tübingen 72076, Germany

**Keywords:** environmental monitoring, new approach methodologies (NAMs), effect-directed analysis (EDA), in vitro bioassay, high-resolution mass spectrometry, suspect screening analysis

## Abstract

Complex organic chemical mixtures in aquatic ecosystems may cause adverse effects on aquatic and sediment-dwelling organisms. Identified chemicals typically explain less than 10% of the observed in vitro bioactivities of such complex mixtures extracted from sediments. In a proof-of-concept study, we combined high-resolution fractionation with machine-learning-driven virtual fractionation to identify aryl hydrocarbon receptor (AhR) agonists in sediment from the Elbe River, Germany. Reporter gene assay showed that only apolar fractions activated the AhR but without specificity among them, necessitating additional virtual fractionation after analysis by gas chromatography coupled with high-resolution mass spectrometry (GC-HRMS). Activity and potency predictions by machine learning models for deconvoluted GC-HRMS features from mass spectral reference library matching allowed the identification of 145 AhR-active HRMS features, with 26 chemicals bioanalytically and chemically confirmed, most of which were polycyclic aromatic hydrocarbons (PAHs). With semiquantified concentrations and estimated potency for the tentatively identified chemicals, the mixture effects of the identified agonists accounted for 14% to 47% of AhR activation in sediments, doubling the contribution of known US EPA priority PAHs. This study provides an effective tool for early screening of AhR agonists, serving as a blueprint to identify causative chemicals for other environmentally relevant modes of toxic action.

## Graphical abstract

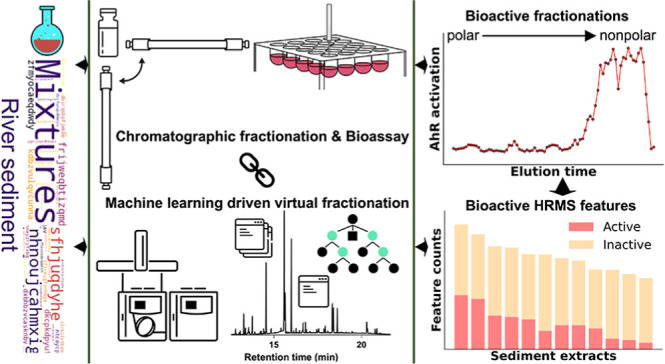

## Introduction

Hundreds to thousands of chemical compounds have been detected in aquatic ecosystems worldwide due to chemical pollution.^1,2^ Emerging chemicals and legacy pollutants threaten aquatic ecosystems and human health.^3,4^ Concerns have grown about the biological pathway disruption effects, referred to as bioactivity, caused by long-term exposure to complex chemical mixtures at environmentally relevant concentrations, which are associated with various chronic toxicities.^5^ Cell-based *in vitro* bioassays are well developed and increasingly used to quantify various bioactivities caused by single chemical compounds or chemical mixtures extracted from environmental matrices.^6,7^ Nevertheless, known chemical contaminants by the chemical target analysis generally account for less than 10% of the observed bioactivity in most frequently studied transcriptional receptor bioassays, such as aryl hydrocarbon receptor (AhR), peroxisome proliferator-activated receptors, and pregnane X receptor.^8^

The AhR is a ligand-activated transcription factor abundant in the liver and barrier organs such as the skin, lung, and gut across various species.^9,10^ Certain xenobiotics, such as 2,3,7,8-tetrachlorodibenzo-p-dioxin (TCDD), can activate AhR by binding to its ligand pocket, causing a conformational change that triggers its translocation from the cytosol to the nucleus in the cell, where it activates the transcription of target genes.^11,12^ Specifically, AhR1 and AhR2 with 2 variants have been identified in fish, with AhR2 being more sensitive than AhR1, while humans and rodents only have one AhR-encoding gene.^13,14^ Activation of AhR is associated with teratogenicity, reproductive toxicity, immune toxicity, cardiotoxicity, dermal toxicity, and hepatotoxicity.^15^ AhR activation is frequently detected in aquatic organisms, surface water, and sediment organic extracts worldwide.^13,16–20^ However, the overall explained percentage remains limited,^21–23^ partly due to the focus on a small set of known AhR agonists and lack of potency data for various chemical classes. There is a need to identify more causative chemical compounds to increase the explained percentage of AhR activation in complex mixtures.

Effect-directed analysis (EDA) has commonly been used for identifying causative chemicals in past decades by integrating bioassay, chromatographic fractionation, and chemical analysis. High-resolution mass spectrometry (HRMS) has been integrated into EDA to broaden the chemical space for identification, when combined with gas chromatography (GC) and liquid chromatography.^24^ Each sample extract can yield thousands or tens of thousands of HRMS features, but their bioactivity often remains unknown, and only a subset of the underlying compounds are bioactive. Bioassays combined with the chromatographic fractionation of sample extracts allow us to reduce complexity by constantly isolating primary bioactive fractions, but this reduction is often labor-intensive and low-throughput. Existing efforts aim to enhance EDA efficiency by combining high-throughput bioassays and high-resolution fractionation (at second scale) with the help of automation,^25,26^ reducing complexity by avoiding repeated fractionation and decreasing the number of HRMS features per fraction.^27–30^

However, successfully identifying causative chemicals that explain a higher percentage of bioactivity in sample extracts using high-throughput EDA remains difficult. Efficiently separating bioactive compounds from complex mixtures is still challenging. The complexity is reduced only if a few fractions experimentally prove bioactive, which may depend on the chemical space of sample extracts and bioactivity end point of measurement. Limited experimental evidence hampers our understanding of fractionation efficacy. Even when a few fractions are bioactive, each may contain hundreds or thousands of HRMS features, complicating bioactive feature identification. Furthermore, bioactive feature separation alone is insufficient, and bioactivity potency is required to be quantified for further risk assessment. Previous studies have shown that machine learning model can be used to efficiently annotate bioactive and inactive HRMS features, thus guiding the prioritization of chemicals.^31–33^ Nevertheless, current nontarget analysis workflows lack tools that integrate semiquantification with bioactivity potency prediction for tentatively identified HRMS features, limiting the explained percent of the identified chemicals to the observed bioactivity in sample extracts. Moreover, few machine learning models exist for bioactivity potency prediction, while most are limited to categorical responses (active/inactive). New approaches are warranted to enable early screening for bioactive compounds in a comprehensive, rapid, and robust manner.

In this study, we performed a high-performance EDA analysis of AhR agonists in sediment organic extracts from the Elbe River basin in Germany by integrating AhR CALUX (chemically activated luciferase expression) in vitro bioassay, high-resolution chromatographic fractionation, gas chromatography coupled with high-resolution mass spectrometry (GC-HRMS), and machine learning models (Figure 1A,B). High-resolution chromatographic fractionation combined with high-throughput AhR CALUX bioassay was expected to reduce the chemical space for suspect screening. Machine learning models were developed to predict from molecular structures AhR-active and AhR-inactive molecules using a binary classification model as well as a regression model to predict potency. The classification model was then integrated with mass spectral reference library matching to effectively screen AhR-active HRMS features, enabling virtual fractionation and isomer-level structure elucidation. The mixture effects of the identified AhR agonists were modeled, and their contribution to the observed AhR activation effects in sediments was ultimately quantified.

**Figure 1:**
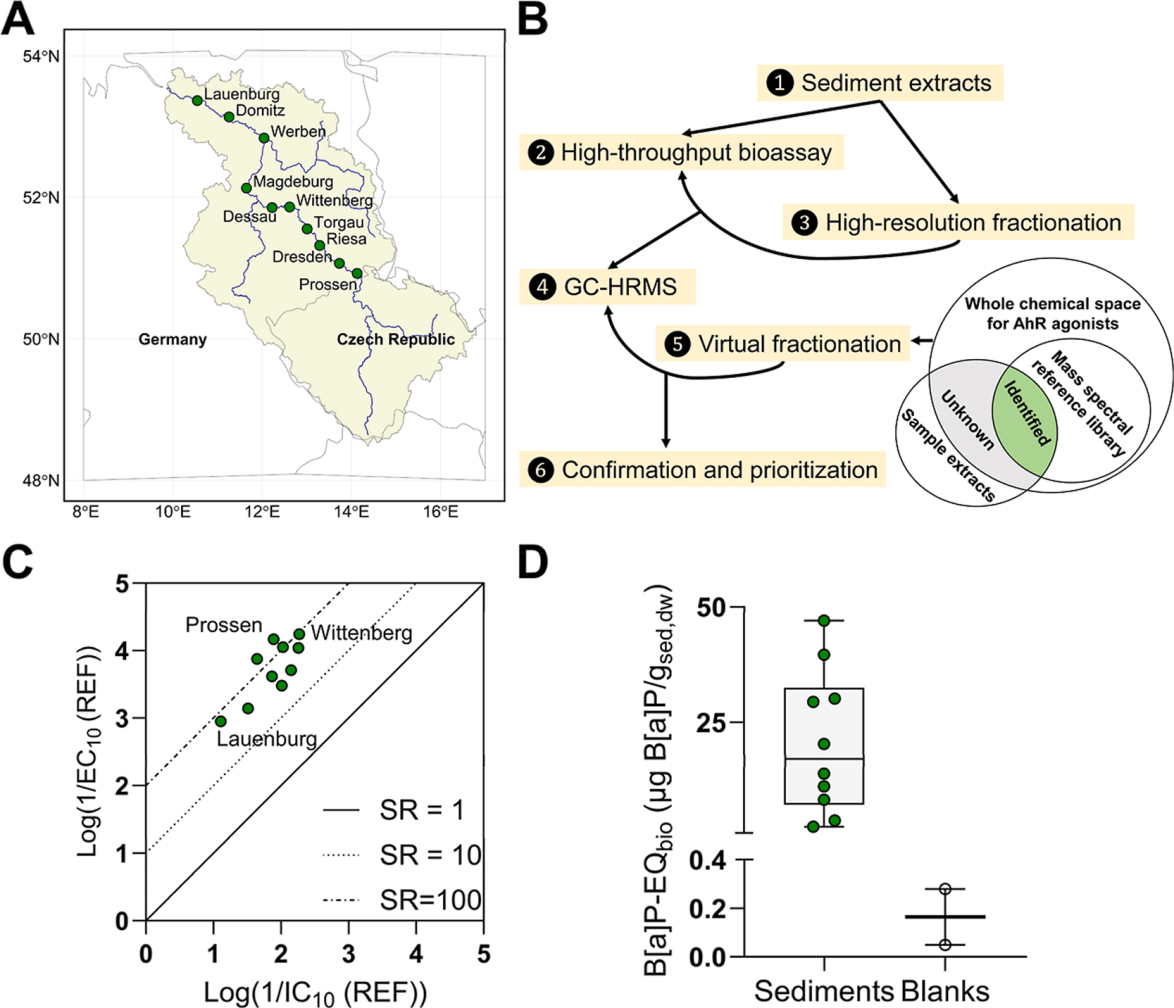
High-performance EDA of AhR agonists in sediments. (A) Sampling sites (green points) shown as sample names in Elbe River basin (beige region). The sampling site names are shown in Table S1; (B) High-performance EDA workflow illustration; (C) Cytotoxicity IC_10_ and EC_10_ for activation of the AhR with high specificity ratios (SR) indicated by diagonal lines; (D) Bioanalytical equivalent concentrations relative to benzo­[*a*]­pyrene (B­[*a*]­P-EQ_bio_) of the Elbe River sediment and two extraction blanks (Blanks).

## Materials and Methods

### Sampling and Sediment Sample Extraction

Ten composite sediment samples were collected by a grab sampler at calm sites such as harbor basins in the Elbe River basin. We sampled the 585 km long region of the free-flowing River Elbe in Germany located between the upstream Czech part which is disturbed by numerous impoundments and the tide-affected part downstream of Geesthacht. Sedimentation occurs always mainly at calm sites and low flow velocity in the shore regions (e.g., groin fields and harbor basins), while the middle of the river is characterized by shifting sand and erosion. The sediment samples were sieved to 63 μm and freeze-dried. The total organic carbon (TOC) content was analyzed for each sample by a TOC analyzer. The coordinates of sampling sites and the corresponding sample names are provided in Table S1 in the Supporting Information.

Organic chemicals were extracted from sediments (*n* = 10) by a pressurized liquid extraction method using an automated solvent extraction system (EDGE, CEM Corporation). Two extraction blank samples were prepared using the same procedure. The extraction and cleanup were conducted according to a previously published standard operation procedure with minor modifications.^34^ The organic extracts from sediments had an enrichment factor (EF, the mass of the sediment sample, g, for extraction divided by the methanol volume in the final stock, mL) ranging from 0.513 to 1.23 g_sed_,_dw_/mL_methanol_. For the extraction blank sample extracts, the EF was set at 1 g_sed,dw_/mL_methanol_. The extracts were stored at −20 °C until further bioassay, fractionation, and chemical analysis. Details on sampling campaigns and extractions are given in Text S1 and Table S2 in the Supporting Information.

### High-Resolution Chromatographic Fractionation

The organic extract was fractionated by high-performance liquid chromatography (Vanquish, Thermo Scientific) using a reversed-phase C18 column (Waters Acquity UPLC BEH C18 1.7 μm, 2.1 × 100 mm). A 10 μL aliquot of the methanolic sediment extract was injected and eluted through a three-Eluent gradient consisting of water, methanol, and acetonitrile in sequence. Details of the gradient are provided in Text S2 in the Supporting Information. The Eluent was collected into 96-well plates (flat bottom, nonpyrogenic, polystyrene, Corning) using the FractioMate collector (from SPARKHolland & VU, Emmen & Amsterdam, The Netherlands). This method was adapted from a previous study.^33^

Briefly, each fraction spreads over 18 s. Eighty fractions were designed for collection over 24 min of elution, but only 78 fractions were collected due to a slight delay between spots. A total of three 96-well plates were reproduced for each sediment sample, including two blank extraction samples. The setup parameters of the FractionMate are provided in Table S3 in the Supporting Information. Afterward, the 96-well plates were evaporated to dryness at 37 °C under a gentle flow of nitrogen gas using a sample concentrator for 96-well plates (TECHNE, Fisher Scientific, Pittsburgh, USA) and stored at −80 °C until further analysis.

### AhR CALUX Bioassay

The AhR CALUX cell bioassay was performed for sample extracts, fractions, and reference compounds. The cell line H4L7.5c2 used in this study was derived from rat hepatoma (H4IIE) and obtained by courtesy of Prof. Michael Denison, UC Davis, USA.^35^

For dosing an aliquot of methanolic sediment extracts (*n* = 10) and extraction blanks (*n* = 2), the sample extract was placed in a 1.5 mL clear glass vial with an inner cone, evaporated to complete dryness using a Barkey evaporator under gentle nitrogen flow, and then reconstituted with the cell medium. This sample was serially diluted using a Hamilton Microlab STAR pipetting platform (Hamilton, Bonaduz, Switzerland), resulting in 11 concentrations, in a 96-well plate. An aliquot of 10 μL each of the dilution was transferred to a 384-well plate containing 30 μL cell medium and 3250 cells per well that had been preincubated for 24 h.

In each bioassay, the cells were exposed to chemicals for 24 h. The cell confluency was detected using an IncuCyte S3 live cell imager (Sartorius, Göttingen, Germany) before and after exposure. Afterward, the luminescence was measured for each plate. Details of the quantification of the reporter enzyme luciferase can be found in our previous study.^23^

The relative enrichment factor (REF) for the sample in the 384-well plate was calculated using eq 1 by the multiplication of EF and dilution factor, volume from the methanolic stock (mL) divided by the final volume in the bioassay medium (mL).

1 REF=EF×DF

For the sediment samples, the final REF in the bioassay ranged from 5.13 × 10^–3^ to 1.23 × 10^–2^ g_sed,dw_/mL_bioassay medium_ after reconstitution with the cell medium. For extracts of extraction blank samples, the REF was 0.01 g_sed,dw_/mL_bioassay medium_ in the 384-well plate. Each concentration had two technical replicates. TCDD was used as a positive control reference, with concentrations ranging from 2.08 × 10^–14^ M to 2.13 × 10^–10^ M. TCDD reference standards were tested in each cell plate, and all experiments were repeated three times.

In addition, the AhR-responsive CALUX bioassay was also conducted for the mixtures of sediment organic extracts for probing the hypothesis of concentration addition, sediment fractions for screening AhR-active fractions, and 30 chemicals for bioanalytical confirmation using the same procedure as that mentioned above. Details are elaborated in Text S3 in the Supporting Information. Chemical information is provided in Table S4 in the Supporting Information.

### Bioassay Data Evaluation

#### Concentration–Response Curve Evaluation

The bioassay data were evaluated following our previously published workflow.^36^ The nominal concentrations at which the chemical exerts 10% of the maximum AhR activation effect (EC_10_) of the positive control (TCDD) and cytotoxicity reaches absolute 10% (IC_10_) were estimated from the concentration–response curve. The specificity ratio (SR_cytotoxicity_) value was calculated (SR = IC_10_/EC_10_). For details, see Text S4 in the Supporting Information.

#### Bioanalytical Equivalent Concentrations and Effect-Based Trigger Values for Sediments

Bioanalytical equivalent concentrations (BEQs) relative to benzo­[*a*]­pyrene (B­[*a*]­P) were calculated for sediment extracts (B­[*a*]­P-EQ_bio_) using eq 2 ^37^ with an EC_10_ for B­[*a*]P of 108 ± 7 μM (2.67 ± 0.17 μg/L).

2 $${B\left[a\right]P‐EQ}_{bio}=\frac{{EC}_{10}\left(B\left[a\right]P\right)}{{EC}_{10}\left(sedimentextracts\right)}$$

B­[*a*]­P-EQ_bio_ values are typically expressed in units of μg_B[*a*]P_/g_sed,dw_, that is, per sediment dry weight (dw). As organic chemicals bind mainly to the OC, B­[*a*]­P-EQ_bio_ can also be expressed in units of μg_B[*a*]P_/g_OC_, by normalizing to the TOC content of the sediments (Table S1).

In the literature, BEQ is often expressed as TCDD-EQ for sediments and as B­[*a*]­P-EQ for single compounds. To enable comparison with literature data, we converted TCDD-EQ_bio_ from the literature to B­[*a*]­P-EQ_bio_ with eq 3.

3 $${B\left[a\right]P‐EQ}_{bio}={TCDD‐EQ}_{bio}\frac{{EC}_{10}\left(B\left[a\right]P\right)}{{EC}_{10}\left(TCDD\right)}$$

As there are no effect-based trigger values (EBT) available for sediments, in previous work, we have suggested to use the OC–water partition constant *K*_oc_ of the reference chemical B­[*a*]P to convert the EBT-BEQ from water to sediment using eq 4.^38^ With a log *K*_oc_ of 4.82, the EBT- B­[*a*]­P-EQ value amounts to 16.4 μg_B[*a*]P_/g_OC_.

4 $$EBT‐BEQ\;\left(sediment\right)=K_{oc}\times EBT‐BEQ\left(water\right)$$

We can evaluate if the toxic burden is rather in the water phase or in the sediments by defining a bioassay-derived distribution ratio of B­[*a*]­P-EQ between sediments and water (*D*_sed/w_) with eq 5.

5 $$D_{sed/w}=\frac{BEQ\left(sediment\right)}{BEQ\left(water\right)}$$

#### Iceberg Modeling

Cumulative bioanalytical equivalent concentrations for chemicals with known concentrations (B­[*a*]­P-EQ_chem_) were calculated by using eq 6, in which BEQ_i_ represents individual chemicals. The relative effect potency (REP) to the reference compound B­[*a*]P for each chemical was calculated using eq 7.

6 $${B\left[a\right]P‐EQ}_{chem}=\sum_{i=1}^{n}{REP}_{i}C_{i}=\sum_{i=1}^{n}{BEQ}_{i}$$

7 $$REP=\frac{{EC}_{10}\left(B\left[a\right]P\right)}{{EC}_{10}\left(chemical\right)}$$

The contribution of the identified chemicals to the observed AhR activation caused by the organic extracts from sediment samples can be quantified with eq 8 by comparing the B­[*a*]­P-EQ_chem_ and B­[*a*]­P-EQ_bio_ values.

8 $$contribution=\frac{{B\left[a\right]P‐EQ}_{chem}}{{B\left[a\right]P‐EQ}_{bio}}$$

### Machine Learning

Two machine learning models were established to predict the binding affinity of molecular structures to AhR and the potency of the AhR agonists. Specifically, a binary classification model was trained to distinguish AhR agonists from inactive molecular structures, and a regression model was trained to predict the EC_10_ values (μM) of these agonists. Very few machine learning models can predict EC_10_ values, and the diagnosis thresholds of active and inactive AhR agonists in the training data set vary greatly for the available machine learning classification models. It is preferable to train classification and regression models specific to the same end point using the same training data set and molecular descriptors.

To this end, the concentration–response curves for 8116 chemical substances from the Tox21 data set (TOX21_AhR_LUC_Agonist) were re-evaluated to reclassify activity (the threshold was set at efficacy >10% of the reference compound and a good linear fit) and derive EC_10_ values according to our previous method.^39^ Details are provided in Text S5 of the Supporting Information and Figure S1.

The Tox21 data set contains few dioxin-like compounds (DLCs), which are well-known potent AhR agonists.^40^ To enhance structural diversity and improve the classification model’s performance, 29 DLCs, including chlorinated dibenzo-p-dioxins (*n* = 7), chlorinated dibenzofurans (*n* = 10), non-ortho-substituted polychlorinated biphenyls (PCBs) (*n* = 4), and mono-ortho-substituted PCBs (*n* = 8), were added to the training data set (Table S5 in the Supporting Information).^41^ Two di-ortho-substituted PCB congeners (PCB 153 and PCB 209) were also added to the training data set; PCB 153 was confirmed inactive, and PCB 209 was confirmed active in this study. Additionally, 14 polycyclic aromatic hydrocarbons (PAHs) that were confirmed in the present study were included in the training data set, many of which are either within the US EPA priority list or are known to be AhR agonists (Table S21 in the Supporting Information).

To better characterize the molecular structure and predict the binding affinity to the AhR ligand pocket, 3D coordinates of chemicals were retrieved from the PubChem database in an SDF format. To this end, CIDs, compound identifiers in the PubChem database, were obtained using chemical names, and parent CIDs were retrieved using PubChem Identifier Exchange Service (https://pubchem.ncbi.nlm.nih.gov/idexchange/idexchange.cgi) to retain main substructures typical of mixtures or salts.

Ultimately, there were 5778 molecules curated, including 832 AhR agonists and 4946 inactive compounds (Table S8 in the Supporting Information). An in-house data set was curated and used as an unseen test set (*n* = 60, Table S9 in the Supporting Information ). A subset of the curated data set was further refined for regression model training (Table S10 in the Supporting Information). A total of 1826 molecular descriptors—1613 2D and 213 3D—were calculated for each molecular structure using the Mordred Python package.^42^ Descriptors with correlations above 0.95 were reduced by retaining only one from each group, leaving 529 numerical descriptors. These were normalized between 0 and 1 based on their minimum and maximum values to define the model’s applicability domain. The eXtreme gradient boosting, XGBoost, was used in this study to train both the binary classification model (XGBClassifier, version 3.0.2) and the regression model (XGBregressor, version 2.1.4). Details on model training are elaborated in Text S5 in the Supporting Information.

### Chemical Analysis

The organic extracts from sediment samples were analyzed by GC-HRMS, comprising a Gerstel MPS autosampler with a TDU3 thermodesorption unit, a Trace 1300 GC (Thermo) instrument with a Gerstel Cold Injection System, and a Q-Exactive HF mass spectrometer (Thermo). A 5 μL aliquot from the sediment extract was injected. An alkane mixture standard (Sigma-Aldrich) at 1 μg/mL was measured along with the samples in every sequence to calculate Kovats’ retention index for each HRMS feature. Details for the HRMS setup are provided in Text S6 and Table S6 in the Supporting Information.

### Suspect Screening Analysis

To both comprehensively and reliably identify AhR agonists in the organic extracts of sediment samples, all of the detected GC-HRMS features, defined by a deconvoluted mass spectrum and retention time (RT), were identified by mass spectral reference library NIST 23 matching. A suspect screening analysis workflow was established, including peak detection and deconvolution, GC-HRMS feature prefilter, mass spectra reference library matching, AhR activation prediction for molecular structure candidates, and identification of HRMS features and structure elucidation at an isomer-group level. Details are given in Text S7 and Table S7 in the Supporting Information.

The identified AhR agonists in sediment extracts were assigned with a communicating confidence level (CL) of 1, 2, or 3, with CL 1 indicating a confirmed structure by GC-EI spectra and RT matching with the reference standard; CL 2 indicating a probable structure based on definitive spectral database-matching results on predefined thresholds within Kovats retention index tolerance (200); CL 3 indicating a tentative structure based on chromatography and characteristic ions but without deconvolution of GC-EI spectra.^43^

### Quantification

The identified features were quantified using the available reference standards, and tentatively identified features were semiquantified using the calibration curve of features with the available reference standards in the same structure similarity group. In addition, 16 EPA PAHs were identified using a target analysis method. Analyte concentrations were quantified by using calibration curves after normalization with internal standards (50 ng/mL in each vial by spiking before instrumental analysis) to correct instrumental variation during each injection and sequence. For analytes with retention times below 12 min, the model ion area was normalized to acenaphthene-D10 (RT = 10.41 min); for retention times between 12 and 19 min to PCB118-13C12 (RT = 16.83 min); and for retention times above 19 min to benzo­[*a*]­pyrene-D12 (RT = 20.82 min). Details for deriving method detection limits (MDLs) are provided in Text S8 in the Supporting Information.

## Results and Discussion

### High Specific AhR Activation Caused by Sediment Organic Extracts

All sediments induced dose-dependent AhR activation effects at REF ranging from 5.01 × 10^–6^ to 1.23 × 10^–2^ g_sed,dw_/mL_bioassay medium_, with efficacy up to 100% of the positive control TCDD (Figure S2). The EC_10_ and IC_10_ values were derived from the concentration response curve of the sediment extracts (Table S11). As shown in Figure 1C, all sediment extracts showed highly specific AhR activation effects, with SR ranging from 29 to 188. An SR of 1 indicates that the activation at AhR only occurs at concentrations that are already cytotoxic (i.e., unspecific activation), and the SR value higher than 10 indicates a high specificity of AhR activation.^39^ The equivolume and equipotent mixture experiments with the sediment extracts demonstrated that the extracts act according to concentration addition (Text S9 in the Supporting Information and Figures S3 and S4). Therefore, it can be assumed that individual chemicals in each extract act as concentration-additives.

The B­[*a*]­P-EQ_bio_ values ranged from 2.39 to 47 μg_B[*a*]P_/g_sed,dw_ (Figure 1D and Table S11). The Prossen sediments had the highest B­[*a*]­P-EQ after TOC normalization, whereas the sediment from Lauenburg had the lowest. The extraction blank contributed 0.4–7.0% of the sediment B­[*a*]­P-EQ_bio_ using the average of blank B­[*a*]­P-EQ_bio_ values, and therefore no blank subtraction was necessary. Previous studies on the River Elbe in Germany reported a mean B­[*a*]­P-EQ_bio_ of 5.7 μg_B[*a*]P_/g_sed,dw_, with only organic extracts from Riesa and Lauenburg showing lower B­[*a*]­P-EQ_bio_.^44^ Globally, B­[*a*]­P-EQ_bio_ for the organic extracts from the sediment samples ranged over 3 orders of magnitude from ng_B[*a*]P_/g_sed,dw_ to μg_B[*a*]P_/g_sed,dw_.^45–47^ The B­[*a*]­P-EQ_bio_ levels in Elbe River sediments in this study were higher than those in the sediment extracts from many surface waters worldwide, such as Tai Lake^46^ and Three Gorges Reservoir^16^ in China and Ulsan Bay in South Korea.^18^

All Elbe sediments exceeded the effect-based trigger values (EBT) derived for sediments of 16.4 μg_B[*a*]P_/g_OC_ ^38^ by a factor of 3 (Lauenburg) to 60 (Prossen, Table S11). The average of bioassay-derived log *D*_sed/w_ was 4.02, close to the mean log *K*_oc_ for B­[*a*]P of 4.82,^38^ indicating that AhR agonists in Elbe sediments were primarily hydrophobic organic compounds. That the majority of AhR-activating compounds is bound to sediments is also consistent with none of the water samples of the Elbe River exceeding the EBT-B­[*a*]­P-EQ_bio_(water) value.^23^

### Screening AhR-Active Mass Spectrometry Features within a Reduced Chemical Space Using Chromatographic and Virtual Fractionation

#### High-Resolution Chromatographic Fractionation for Screening AhR-Active Fractions

Each sediment extract was fractionated by high-performance liquid chromatography into 80 fractions, and each fraction was tested in AhR CALUX bioassay at one constant REF per sample ranging from 1.28 × 10^–2^ to 3.08 × 10^–2^ g_sed,dw_/mL_bioassay medium_ for the different sediments. All fractions of two blank extraction samples exhibited no AhR activation effects (Figure 2A and Figure S5).

**Figure 2:**
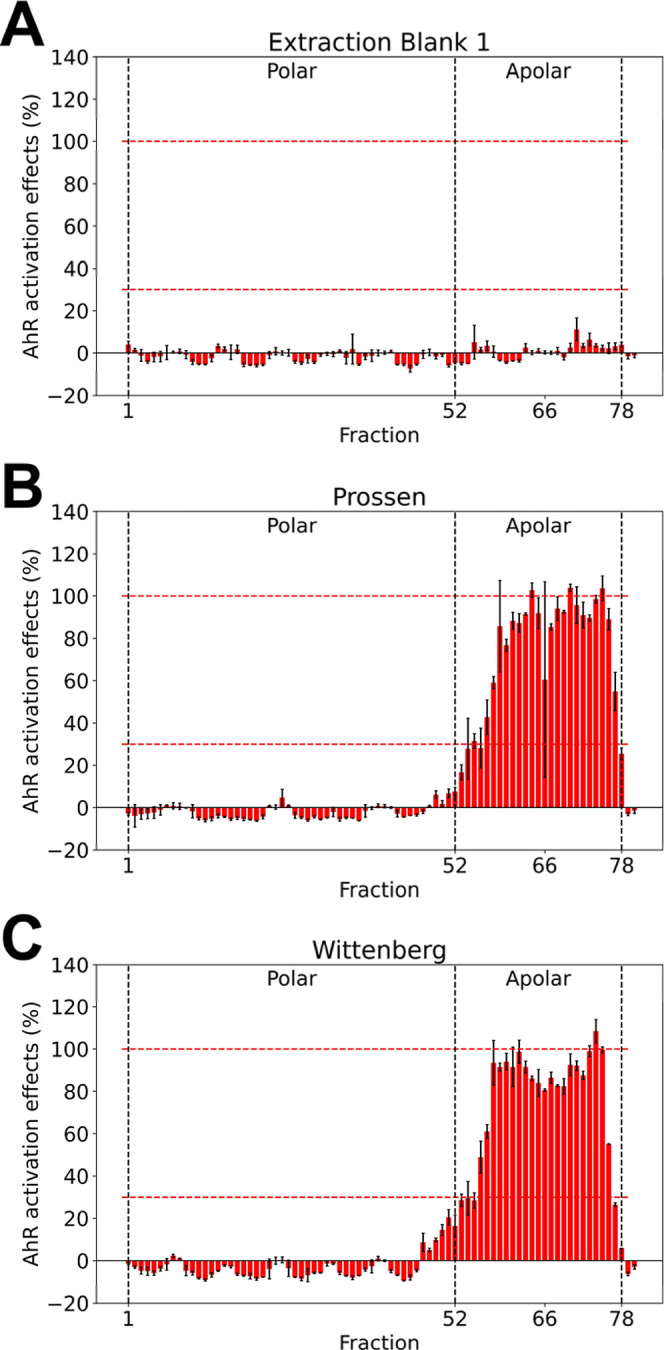
AhR activation in the sample fractions. A total of 80 fractions were produced by high-performance liquid chromatography using a reverse-phase C18 column and distributed in 96-well plates at an REF ranging from 1.28 × 10^–2^ to 3.08 × 10^–2^ g_sed,dw_/mL_bioassay medium_. Only 78 fractions were collected due to slight delay between spots. (A) Extraction blank sample extract; (B) Prossen sediment extract; (C) Wittenberg sediment extract. Each bar in the plot shows the mean value of two replicates with standard error. Results for other sediment extracts are provided in Figure S5.

Sediment extract fractions (Nos. 1–52) eluted by a mixture of water and methanol did not show any AhR activation. In contrast, sediment extract fractions (Nos. 53–80) eluted by pure solvents (methanol and acetonitrile) showed pronounced AhR activation (Figure 2B,C and Figure S5). The AhR activation in fractions 53–80 showed a near-normal distribution in the region where methanol transitioned to acetonitrile as the eluent. The decrease in AhR activation in the last several fractions indicated the mobile phase gradient used in the study effectively eluted most AhR agonists in the organic extracts, which was also confirmed when the recovery of AhR activation after fractionation and testing of recombined fractions ranged from 95% to 158% (Figure S6 and Table S12).

High-resolution fractionation combined with a high-throughput in vitro bioassay considerably narrowed the chemical space for screening AhR agonists in the sediment extracts. Nevertheless, AhR activity was detected in all apolar fractions, indicating that chromatographic fractionation could not reduce the mixture complexity in these fractions. The result differs from cases where a few compounds dominate effects, such as estrogenic or androgenic effects, in other matrices.^48–50^ As such apolar chemicals are more effectively ionized by electron ionization compared to electrospray ionization,^51^ GC-HRMS was used to identify AhR agonists in apolar fractions. Therefore, after chromatographic fractionation, a virtual fractionation of GC-HRMS features was complemented by using a machine learning classification model to separate active AhR from nonactive features.

#### Predicting AhR Activity from Molecular Structures

An ensemble decision tree-based quantitative structure–activity relationship (QSAR) model was developed to predict the AhR activity from molecular structures. The area under the receiver-operating characteristic curve (AUC-ROC) and precision recall curve (AUC-PRC) of the established binary classification model for the overall training data (*n* = 5778) was 98.6% and 96.6%, respectively (Figure 3). The AUC-ROC and AUC-PRC for the unseen test data (*n* = 60) from our in-house data set were 91.8% and 81.8%, respectively (Table S13). Specifically, in the training data set, there were 57 false-negatives and 75 false-positives, yielding 97.7% accuracy, 91.2% precision, and 93.1% recall. For the unseen test data, there was one false-negative and 9 false-positives, with 83.3% accuracy, 57.1% precision, and 92.3% recall (Table S13). The error of the classification model mainly stemmed from false-positives, which is acceptable for a screening purpose since the risk of false-negatives is higher than that of false-positives when 100% accuracy is unattainable.

**Figure 3:**
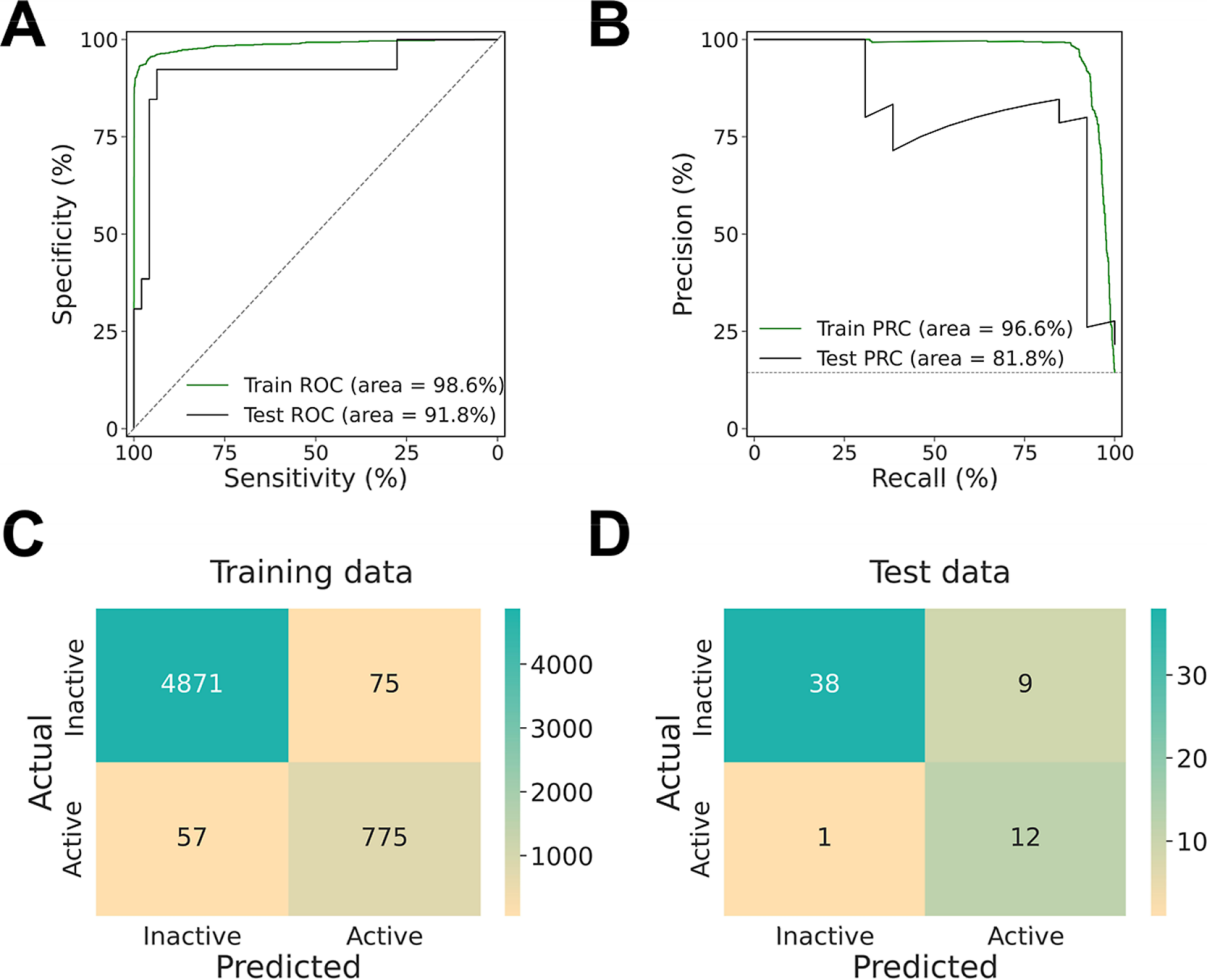
Model performance for the trained binary classification model based on molecular structures, represented by 529 numerical 2D and 3D molecular descriptors, to distinguish AhR agonists from inactive compounds. (A) Receiver-operating characteristic curve (AUC); (B) Precision recall curve (PRC); (C) Confusion matrix for training data; (D) Confusion matrix for test data. The training data sets are from the re-evaluated Tox21 data set plus a set of DLCs as well as 14 PAHs that were biotested in the present study. The test data are from the in-house data set collected from the literature.

DLCs, including chlorinated dibenzo-p-dioxins, chlorinated dibenzofurans, non-ortho-substituted PCBs, and mono-ortho-substituted PCBs, were accurately predicted as active (Table S8). The AhR activities of PAHs in the training data set were correctly predicted except for benzo­[e]­pyrene (B­[e]­P), anthracene, and phenanthrene. B­[e]P was active in the Tox21 data set and predicted active but inactive in our bioassay. Anthracene was predicted and tested active, but it had an SR of 1, indicating it activated AhR under cytotoxicity. Phenanthrene was inactive in Tox21 and was predicted to be inactive but active with low potency in our bioassay.

A good classification model is indispensable for accurate virtual fractionation. Growing interest exists in integrating machine learning into nontarget analysis with many machine learning model architectures, including deep learning models trained on diverse data sets.^52^ However, the lack of large-scale benchmark data sets limits the comparability and robustness of model predictions, as studies often use varying predefined thresholds to classify training data. For example, some chemicals might be considered inactive if a 30% efficacy threshold is applied.^53^ Several PAHs, including phenanthrene and anthracene, were considered AhR-inactive in previous studies.^54,55^

Among 529 2D and 3D molecular descriptors, the top-ranking 100 features suggest planar structure and geometry (three-dimensional spatial structure of a molecule) were both important for AhR binding (Figure S7). Overall, the established machine learning binary classification model can effectively classify AhR activation effects from molecular structures with a very high prediction accuracy within the application domain (Table S14).

#### ML-Based Virtual Fractionation for Screening AhR-Active Features Detected by GC-HRMS

A streamlined suspect screening workflow was established in the study (Figure 4A and details in Figure S8). Approximately 3000 GC-HRMS features (2801 to 3061) were obtained after deconvolution using MS-DIAL in the sediment extracts. After sequential filtering, as shown in Figure S9, approximately 259 to 393 of these features had matching hits in the NIST 23 spectral reference library based on the defined threshold: matching factor >700, reverse matching factor >700, and Kovats’ retention index tolerance <200 (Table S15). Matched HRMS features either had unique top candidates or multiple features mapped to the same candidates, suggesting they are isomers due to the same or similar deconvoluted mass spectra and close RT (for detailed results, see Zenodo^56^).

**Figure 4:**
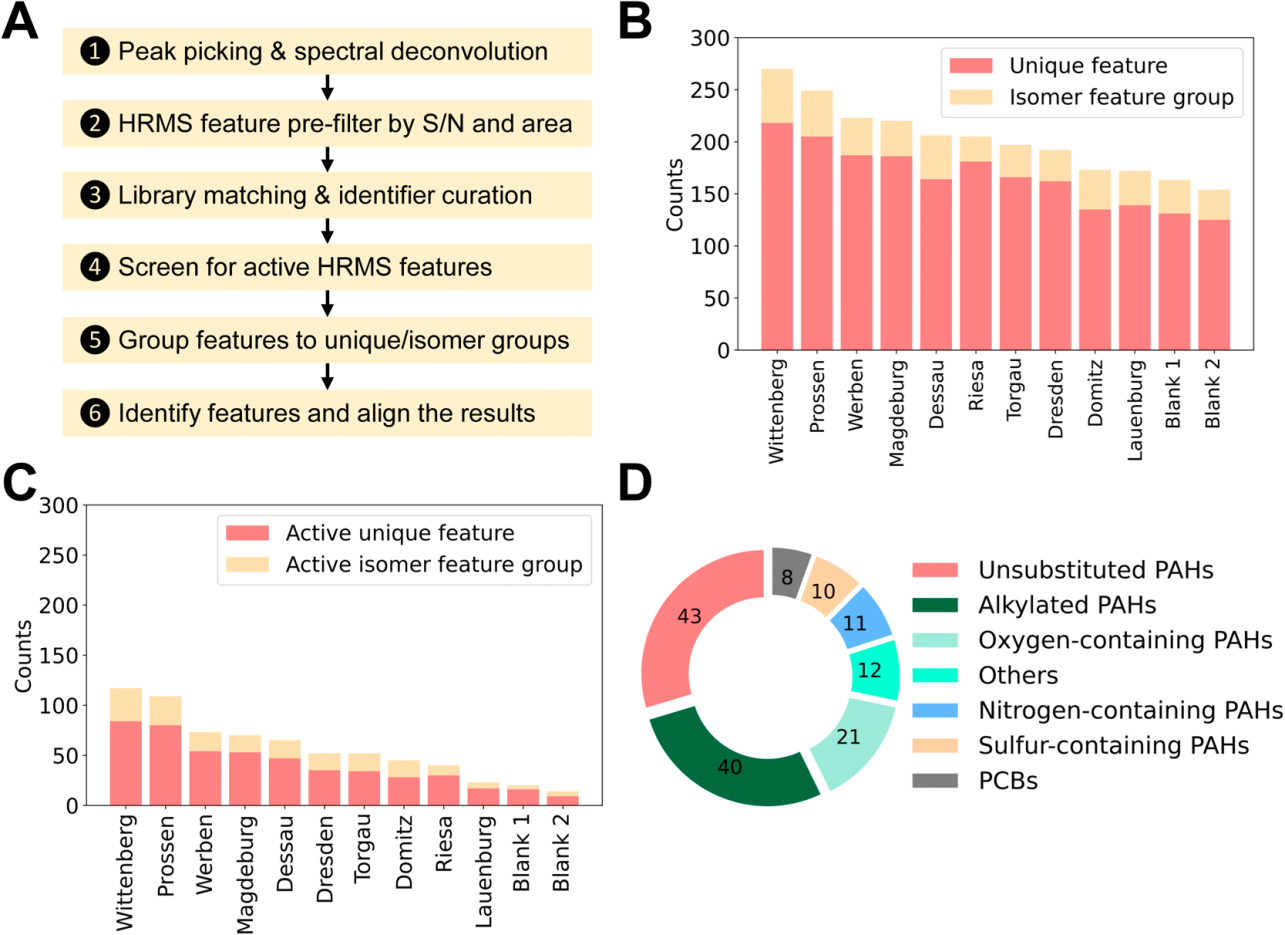
Suspect screening for AhR agonists in sediment organic extracts. Virtual fractionation is integrated to screen for bioactive deconvoluted GC-HRMS features to further reduce the complexity of mixtures after high-resolution fractionation. (A) Suspect screening analysis workflow for GC-HRMS features; (B) Deconvoluted GC-HRMS feature numbers with a definitive match against the NIST spectral reference library. Isomer feature group contains more than one HRMS feature. For feature counts following serial filter based on Figure 4A, see Figure S9; (C) Deconvoluted GC-HRMS feature after applying established binary classification model prediction on top-ranking candidates for each feature or feature group; (D) Tentatively identified AhR agonists and their distribution across chemical structure groups. PCBs, polychlorinated biphenyls.

Only 30% of prefiltered deconvoluted mass spectra matched the reference spectral libraries with reliable hits; the remaining HRMS features remain unknown. It is reasonable to assume that many other AhR agonists exist among these unknowns and should contribute to the activation observed in sample extracts. In addition, there are also many HRMS features with low abundance, which might lead to GC-EI spectra of poor quality after deconvolution.^57^ It is also challenging to elucidate the molecular structures of these features, and some of them might also be AhR-active.

To elucidate the molecular structures of matched HRMS features, they were divided into two groups: those with a single feature (referred to as a unique feature) and those with multiple features mapped to the same top-ranking candidates (referred to as isomer feature groups, Figure 4B). The established machine learning classification model was used to predict the AhR activity of top-ranking candidates, and features were filtered out if none of the top-ranking candidates were predicted active. More than 50% of the features were filtered out (Figure 4C). For example, HRMS feature groups in the Prossen sediment extract dropped from 249 in total to 109 groups that were predicted to be AhR-active. Among these groups, 80 of 205 unique features and 29 of 44 isomer feature groups were predicted to be AhR-active (Table S16).

Notably, the vast majority of top-ranking candidates were not in the training data set (Figure S10) but still fell within the applicability domain of the trained classification model, covering 95% of these candidates (Table S34). The Prossen and Wittenberg sediments contained the largest numbers of predicted AhR-active top-ranking candidates from the NIST library, and most of them were overlapped with other sample extracts (Figure S11). Therefore, the Prossen and Wittenberg sediments were used as quality control samples for the GC-HRMS feature identification (Table S17).

### Identification and Characterization of AhR Agonists in Sediment Extracts

A total of 145 AhR-activating chemicals were tentatively identified in sediment extracts by the suspect screening analysis workflow (Tables S18 and S19). Of those, we analytically confirmed 31 chemicals that were assigned a CL of 1 ^43^ based on available authentic standards by matching GC-EI mass spectra and RT (Figure 5A and Figure S12). Moreover, 105 were tentatively identified with CL at 2 with definitive reference spectral library matching (Figure 5B and Table S19). Additionally, eight PCBs were tentatively identified using a manual search for molecular ions (Table S20 and Figure S13). However, the low abundance of the ions caused MS-DIAL to fail the deconvolution, preventing the matching of deconvoluted mass spectra against either the NIST library or reference standards for analytical confirmation. Therefore, they were assigned a CL of 3. The identified AhR agonists were mainly PAHs, including the most abundant unsubstituted PAHs, alkylated PAHs, and heterocyclic PAHs containing oxygen, sulfur, and nitrogen (Figure 4D).

**Figure 5:**
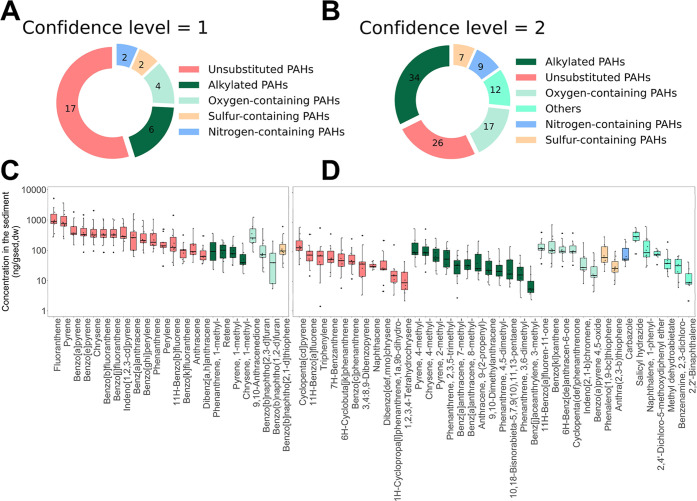
Identification and quantification of AhR agonists in the sediments. Distribution of identified chemical compounds with CL 1 (CL) (A) and tentatively identified chemical compounds with CL 2; (B) across different structural groups; (C) Quantification of CL 1 chemical compounds shown in box plots in decreasing orders based on median concentrations from sampling sites with detection frequency ≥90% in the sediment samples; (D) Semiquantification of CL 2 chemical compounds with detection frequency ≥90% in the sediment samples.

HRMS features within isomer groups often share similar retention times, complicating their separation on a GC column. Benzo­[*b*]­fluoranthene (B­[*b*]­F) and benzo­[*j*]­fluoranthene (B­[*j*]­F) exhibited nearly identical retention times at 1 μg/mL using a single reference standard, consistent with their similar retention indices (2704 and 2707, respectively). When mixed, they coelute into a single peak, as seen in the first chromatogram in Figure S12. Thus, the peak at the same RT in Prossen likely represents a mixture of both compounds. Benzo­[*b*]­naphtho­[2,1-*d*]­thiophene and benzo­[*b*]­naphtho­[2,3-*d*]­thiophene likely coeluted. Additionally, some groups have many top-ranking candidates mapped to fewer detected features, making it challenging to assign the correct candidate to each HRMS feature without the authentic reference standards.

To validate the AhR activation classification predictions of the identified chemicals, 26 of the 31 identified chemicals at a CL of 1 were tested in the AhR CALUX bioassay. Of these, 12 PAHs were all confirmed to be AhR-active in the bioassay, which further validated our ML classification model prediction accuracy (Figure S14 and Table S21). It is known that molecular geometry is crucial for activating the AhR ligand pocket. Most AhR agonists have planar structures. Typically, chlorine substitution at the ortho position of PCB congeners, especially di-ortho-PCBs, reduces their planar structure, thereby decreasing their AhR activation potency.^58^ PCB126 was significantly more potent than di-ortho-PCBs, PCB 138 and PCB 153 in the AhR CALUX, with PCB 153 being AhR-inactive at the tested concentrations. Alkylated PAHs were more potent than their corresponding unsubstituted PAHs. For example, 2-methylanthracene and 2-methylphenanthrene were more potent than anthracene and phenanthrene. Alkylated chrysene was more potent than chrysene, which is consistent with previous studies.^14,59,60^ The biotested PAHs and their derivatives in this study were confirmed to be AhR agonists, which generally aligns with previous studies using rat and fish cell lines despite of variations in the potency.^14,54,60^

Interestingly, an activity cliff was observed for PAH isomers with 3 to 6 rings. Round-fused PAHs were much less potent than chain-fused PAHs, which may explain the significant differences in the AhR activation potency among isomers. For example, pyrene and fluoranthene, which can be viewed as round-fused PAHs, are about 2 orders of magnitude less potent than chrysene and benz­[*a*]­anthracene (Table S21), which can be viewed as chain-fused PAHs, yet they are 4-ring PAHs. Moreover, benzo­[e]­pyrene (REP = 2.7 × 10^–4^B­[*a*]­P) and perylene (REP = 7.8 × 10^–4^B­[*a*]­P) are very weak AhR agonists. In contrast, their isomers, including B­[*b*]F (REP = 2.2B­[*a*]­P), B­[*j*]F (REP = 5.3B­[*a*]­P), and B­[k]F (REP = 5.2B­[*a*]­P) are very potent AhR agonists. The plausible explanation is that benzo­[e]­pyrene and perylene are more like round-fused PAHs compared to the other four isomers.

The results suggested the topology of fused rings within a planar structure, i.e., their fusion and arrangement also greatly influence their AhR binding affinity. Enhanced molecular representation capturing the topology and geometry of molecular structures is warranted to improve classifier accuracy and precision not only in different classes of compounds but also in the activity cliff among isomers.

We quantified 30 chemicals at a CL of 1 based on available authentic standards with internal standard calibration (Figure 5C and Table S22). Moreover, concentrations of the 16 US EPA-prioritized PAHs were also quantified and are separately listed in Table S23. One-hundred and five chemicals (CL2) were semiquantified based on structurally similar chemicals (Figure 5D and Tables S24–S33). Chemicals with CL of 3 were not quantified. Only 34 chemicals were detected in all 10 sediment extracts, suggesting a diverse distribution of identified AhR agonists among different sampling sites (Figure S15). Most AhR agonists were identified in Prossen and Wittenberg sediments, while the fewest were found in Lauenburg sediments. Measured concentrations ranged from below the MDL to 5.2 μg/g_sed,dw_. Fluoranthene and pyrene exhibited the highest concentrations, reaching 5.2 and 3.7 μg/g_sed,dw_, respectively, in Wittenberg sediment.

### Prioritization of Identified AhR Agonists in the Sediments

#### Predicting AhR Agonist Potency

To allow mixture effect modeling, an ensemble decision tree model-based regression model was developed to estimate EC_10_ values of AhR agonists from re-evaluated Tox21 data set and the confirmed AhR agonists in our bioassay (Table S10). The EC_10_ values of the top-ranking candidates for GC-HRMS features were predicted using the regression model (Table S34). The difference between observed and predicted EC_10_ values was generally within a factor of 3 (Figure 6A) for both the overall training data set (RMSE = 0.231, *n* = 253) and unseen test data set (RMSE = 0.712, *n* = 10, Table S13). The regression model overestimated EC_10_ values for several molecules with observed values below 1 μM in the training data set, thus underestimating their AhR activation potency. Nevertheless, the difference between observed and predicted EC_10_ values for these molecules was within a factor of 10.

**Figure 6:**
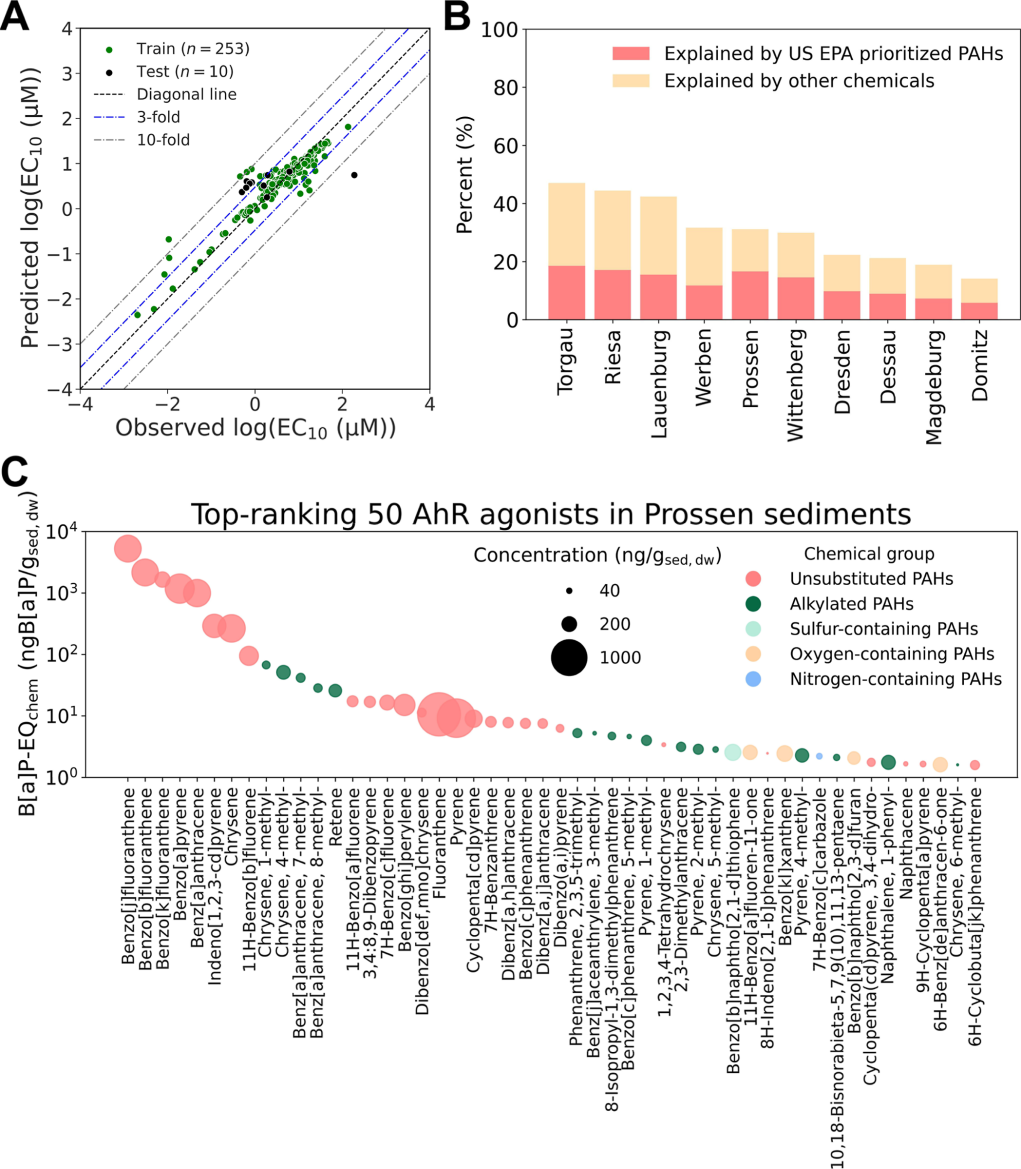
Characterization of bioanalytical equivalent concentrations for the identified AhR agonists in the sediments. (A) Machine learning regression model for predicting EC_10_ values from molecular structures, represented as 529 numerical 2D and 3D molecular descriptors; (B) Explained percentage of identified AhR agonists to the observed AhR activation in the sediment extracts; (C) Prioritized AhR agonists in the Prossen sediment. For other sampling sites, the plots are provided in Figure S16.

Little attention has been paid in the past to the quantitative prediction of effects using machine learning regression models. Lack of potency data hinders prioritizing identified AhR agonists and calculating their BEQ values. The frequently used training data set, such as Tox21 data set, carries a bias toward polar compounds, which are typically less potent AhR agonists. Most of the identified PAHs are not included in the Tox21 data set, but many of them are potent AhR agonists. There is thus a need for acquiring high-quality concentration–response data for a large structurally diverse set of chemical compounds with improved diagnosis for training both classification and regression models.

#### Iceberg Modeling

The predicted BEQ_chem_ of the quantified AhR agonists explained 14% to 47% of BEQ_bio_ of the sediments (Figure 6B, Table S24–S33, and Table S35), while only 6% to 19% of effects were explained by the US EPA priority PAHs (Table S36). For example, 119 identified chemicals from the Prossen sediment accounted for 31% of the effects, while the 12 US EPA priority PAHs explained 17%. Unsubstituted PAHs predominantly contributed to AhR activation in sediment extracts, followed by alkylated PAHs and heterocyclic PAHs (Figure 6C and Figure S16). Among the identified AhR agonists in each sample extract, the top 10 chemicals by BEQ_chem_ values accounted for approximately 97% of AhR activation effects, most of which were quantified by their authentic standards. B­[*j*]F ranked first across all 10 samples, explaining about 37% to 47% (Figure S17). Its isomers B­[*b*]­F, B­[k]­F, and B­[*a*]P made significant contributions, along with 4-ring PAHs chrysene and benz­[*a*]­anthracene and their alkylated isomers.

The AhR activation of heterocyclic PAHs made a limited contribution, resulting from both low occurrence and low potency. For example, benz­[*c*]­acridine (REP = 6.4 × 10^–3^ B­[*a*]­P) was much less potent than benz­[*a*]­anthracene (REP = 1 B­[*a*]­P), despite differing only by a nitrogen substitution in the second benzene ring from the top. Conversion of unsubstituted PAHs to nitro-PAHs is considered a detoxification process with regard to AhR activation for PAHs in diesel particulate.^61^ The detected PCBs also made a limited contribution, which was consistent with previous studies showing that DLCs played a minor role in sediments in Elbe River^43^ (Text S10 in the Supporting Information). PAHs are the most frequently identified AhR agonists in other EDA studies on AhR.^16,18,62^ They exhibited a concentration-additive effect on AhR activation in artificial mixtures in previous studies using designed mixtures,^63^ which agrees with our experimental findings on mixtures of extracts.

Notably, the top contributors, including B­[*j*]­F and B­[*b*]­F, showed both high concentration and strong AhR activation potency. Alkylated PAHs were more potent than their unsubstituted counterparts, with the same number of fused benzene rings. Their much lower concentrations resulted in a smaller contribution to BEQ_chem_ than from their corresponding unsubstituted PAHs. This finding differs from AhR-dependent toxicity in crude oil, where the alkylated PAHs are primary constitutes.^55,64,65^ Since B­[*j*]F and B­[*b*]F coeluted, we assumed equal concentrations. As B­[*j*]F is 2.4 times more potent than B­[*b*]­F (Table S21), changes in their relative ratios would lead to uncertainty in the BEQ_chem_ calculation. Additional uncertainty stems from semiquantification and EC_10_ value prediction for AhR agonists without available authentic reference standards.

### Implications

In routine environmental monitoring, lower concentrations of many diverse chemicals are subtle in causing bioactivity but can still lead to chronic impacts on aquatic organisms and humans. Thus, the early screening of bioactive chemicals in a rapid and comprehensive manner is essential. This study demonstrates that high-resolution fractionation combined with a high-throughput bioassay in a standardized manner helps narrow the chemical space for screening. Virtual fractionation using machine learning models can complement chromatographic fractionation by pushing the limit from screening bioactive fractions to bioactive HRMS features, therefore, efficiently reducing the complexity of the chemical mixtures.

The suspect screening workflow streamlined in this study allows library matching for all deconvoluted mass spectra, virtual fractionation of molecular candidates by a binary classification model, and isomer-level structure elucidation. This workflow greatly improves the accuracy and efficacy of AhR agonist identification in complex chemical mixtures. Machine learning-based regression model enables potency prediction for agonists, thus facilitating mixture effect modeling.

Nevertheless, several limitations still need to be addressed in future studies. More representative chemicals need to be evaluated in *in vitro* bioassays to determine their EC_10_ values, thereby increasing the training data set size and expanding the diversity of chemical categories for both classification and regression models. For identified chemicals that lacked reference standards, the necessity to predict the EC_10_ values and the mere semiquantification introduced inherent uncertainty into BEQ calculations. Although the estimated BEQ_chem_ can identify key chemical classes for prioritization, it should not be used in cumulative risk assessment, unless the potency estimates are experimentally confirmed. Additionally, integrating machine learning, fractionation, and in vitro bioassays is a scientifically sound approach but requires substantial resources. The nontarget analysis workflow developed in this study warrants application to larger geographic areas with more samples to better delineate bioactivity and map diverse bioactive chemical compositions.

This study demonstrated how AhR agonists can be enumerated across a chemical space using a spectral reference library by machine learning models and addressed the data gap by evaluating the currently “known unknowns” (known structures with unknown effects) and quantifying their mixture effects via BEQs. Chromatographic fractionation combined with virtual fractionation offers an effective tool for the early screening of bioactive chemical compounds. Our approach provides a blueprint for identifying bioactive chemicals with other modes of toxic action, facilitating large-scale routine monitoring of these chemicals not only in environmental monitoring of water and sediments but also in ecoexposome and potentially exposome studies.

## Supplementary Material





## Data Availability

GC-HRMS data in Thermo RAW format are available on MassIVE (MSV000100070) with passcode (UFZ) for review. The source data and code for all figures in the main text and Supporting Information are available in the Zenodo database (10.5281/zenodo.17791247). Python scripts for training machine learning models and R script for suspect screening analysis workflow are deposited in GitHub (https://github.com/Haotian2025/vEDA_AhR_Elbe_sediment/tree/main). The inputs for the scripts are available in the Zenodo database (10.5281/zenodo.17791247).

## Notes

Curated training dataset for machine learning classification model; bioassay results for organic extracts of sediment samples; machine learning model performance metrics; retention index for alkane mix standard; summary for identification with confidence level; target screening list for PCBs; quantification results for 16 US EPA-prioritized PAHs; and bioanalytical concentration for identified agonists (XLSX)
Sample extraction, chromatographic fractionation, AhR CALUX bioassay, concentration–response curve evaluation, machine learning model training, chemical analysis, and quantification (PDF)
