# Supplementary material for: Combination of Chromatographic and Machine Learning-Driven Virtual Fractionation Identifies Aryl Hydrocarbon Receptor Agonists in Sediments
Source: Environ Sci Technol. 2026 May 11;60(20):14408–21. doi: 10.1021/acs.est.6c02059 (PMC13217564; doi:10.1021/acs.est.6c02059)
Supplement: Supplementary file 2 [file es6c02059_si_002.pdf]

Supporting Information for

Combination of Chromatographic and Machine Learning-Driven Virtual Fractionation Identifies Aryl Hydrocarbon Receptor Agonists in Sediments

Haotian Wang,*[,1,2] Georg Braun,[1] Norbert Kamjunke,[3] Martin Krauss,[4] Guibin Jiang,[2] Beate I. Escher[1,5]

[1]Department of Cell Toxicology, Helmholtz Centre for Environmental Research–UFZ, Leipzig 04318, Germany

[2]State Key Laboratory of Environmental Chemistry and Toxicology, Research Center for Eco-Environmental Sciences, Chinese Academy of Sciences, Beijing 100085, China

[3]Department of River Ecology, Helmholtz Centre for Environmental Research–UFZ, Magdeburg 39114, Germany

[4]Department of Exposure Science, Helmholtz Centre for Environmental Research–UFZ, Leipzig 04318, Germany

[5]Environmental Toxicology, Department of Geosciences, Eberhard Karls University Tübingen, Tübingen 72076, Germany

*Corresponding author email: haotian.wang@ufz.de, htwang@rcees.ac.cn

This file contains 74 pages, 10 text notes, 7 tables and 17 figures.

There are another 29 tables, including Tables S8–S36 provided in an additional excel file (Supporting Dataset) as they are large tables. The caption of each table is provided in this file.

List of Texts

Text S1. Sampling, sample extraction and clean-up

Text S2. High-resolution chromatographic fractionation

Text S3. AhR CALUX bioassay

Text S4. Concentration-response curve evaluation

Text S5. Machine learning model training

Text S6. Chemical analysis

Text S7. Suspect screening analysis

Text S8. Method detection limits for quantification

Text S9. Additional information on the mixture experiments

Text S10. Additional information on PCB contribution to mixture effects

List of Figures

Figure S1. Tox21 dataset characterization after re-evaluation as the training dataset for machine learning-based binary classification model.

Figure S2. Concentration-response curves of the organic extracts from sediment samples and extraction blank samples in the AhR CALUX bioassay.

Figure S3. Concentration-response curves of the mixed sediment organic extracts in the AhR CALUX bioassay.

Figure S4. Bioassay results for designed mixtures.

Figure S5. AhR activation effects for sediment extract fractions from high-resolution fractionation.

Figure S6. Concentration-response curves of the pooled sediment extract fractions in the AhR CALUX bioassay.

Figure S7. Relative importance of top 100 ranking molecular descriptors in binary classification model.

Figure S8. Streamlined suspect screening workflow for GC-HRMS.

Figure S9. GC-HRMS feature counts in the sediment extracts after each filtering step during suspect screening analysis.

Figure S10. Overlap of chemicals between training data for binary classification and top-ranking candidates after definitive library matching.
Figure S11. Overlap of top-ranking candidates predicted to be AhR-active between Prossen and other sample extracts.
Figure S12. Overlay of extracted ion chromatographs for identified AhR agonists in sample extracts and reference standards, along with their head-to-tail GC-EI spectra.
Figure S13. Total ion chromatograms of PCBs in reference standard and sediment extracts.
Figure S14. Concentration-response curves of the reference standards in the AhR CALUX bioassay.
Figure S15. Intersection of detected AhR agonists in sediment samples.
Figure S16. Prioritization of the top 50 identified AhR agonists based on their bioanalytical equivalent concentrations relative to benzo[a]pyrene (B[a]P-EQ) in each sediment extract.
Figure S17. Relative contribution of the top 10 identified AhR agonists to the cumulative B[a]P-EQ in each sediment sample

List of Tables

Table S1. Sampling information
Table S2. Parameters for sample extraction
Table S3. Parameters for FractioMate collector
Table S4. Reference standards for bioanalytical and chemical confirmation
Table S5. Added dioxins and dioxin-like compounds in classification training dataset
Table S6. Parameters for GC-Orbitrap
Table S7. Parameters for MS-DIAL for HRMS peak picking and deconvolution

List of Tables in Supporting Dataset

Table S8. Curated training dataset for machine learning classification model

Table S9. Unseen test dataset for evaluating binary classification model performance

Table S10. Curated training dataset for machine learning regression model

Table S11. Bioassay results for organic extracts of sediment samples

Table S12. Bioassay results for combined fractions of sediment extracts after high resolution fractionation

Table S13. Machine learning model performance metrics

Table S14. Application domain for selected molecular descriptor

Table S15. Retention index for alkane mix standard

Table S16. GC-HRMS feature statistics during suspect screening analysis

Table S17. Suspect screening analysis results for Prossen and Wittenberg

Table S18. Alignment results for all sediment extracts

Table S19. Summary for identification with confidence level

Table S20. Target screening list for PCBs

Table S21. Estimated EC_{10} values for each tested reference compound

Table S22. Calibration curve and method detection limit for reference standards

Table S23. Quantification results for 16 US EPA prioritized PAHs

Table S24. Bioanalytical concentration for identified agonists in Prossen

Table S25. Bioanalytical concentration for identified agonists in Dresden

Table S26. Bioanalytical concentration for identified agonists in Riesa

Table S27. Bioanalytical concentration for identified agonists in Torgau

Table S28. Bioanalytical concentration for identified agonists in Wittenberg

Table S29. Bioanalytical concentration for identified agonists in Dessau

Table S30. Bioanalytical concentration for identified agonists in Magdeburg

Table S31. Bioanalytical concentration for identified agonists in Werben

Table S32. Bioanalytical concentration for identified agonists in Domitz

Table S33. Bioanalytical concentration for identified agonists in Lauenburg

Table S34. EC_{10} value prediction for top-ranking candidates

Table S35. Iceberg modeling summary

Table S36. Bioanalytical concentration for US EPA PAHs

Text S1. Sampling, sample extraction and clean-up

The sampling campaign on the research vessel Albis took place from July 3 to 11, 2023, covering the freshwater section of the Elbe River within Germany from Prossen near the Czech-German border to Geesthacht.[1]

Organic chemicals were extracted by pressurized liquid extraction (PLE) method using an automated solvent extraction system (EDGE, CEM Corporation). To avoid background contamination, the aluminum Q-Cup sample holder was rinsed with water, acetone and methanol in sequence twice. They were dried in a fume hood to evaporate solvents, and then put in an over at 105°C overnight.

Aliquots of the sediment samples containing 100 mg of TOC were extracted with a 50:50 (v/v) mixture of ethyl acetate and acetone after pre-heating for 5 minutes using two extraction cycles for 5 minutes each at 100 °C. Two procedural blank samples as extraction blank samples were prepared using two empty PLE cells, one extracted before and one after the samples. The extracts were evaporated to approximately 2-3 mL using a Barkey evaporator and transferred to a 20-mL amber glass vial. The collecting glass vial was rinsed twice with 1 mL of dichloromethane (DCM), which was finally added to the amber glass vial. The eluted solvents were evaporated to dryness and reconstituted with 0.5 mL of DCM using glass pipette with stainless steel needle.

For clean-up this extract was transferred onto a silica gel cartridge (CHROMABOND® Flash, RS 4 SiOH, 40-64μm, 4g) using a glass pipette. The glass vial and glass pipette were rinsed with 0.5 mL of DCM and loaded onto the cartridge. A glass syringe was used to gently push the top space of the cartridge to load the elution into the silica gel. The cartridge was eluted sequentially with 7.5 mL of DCM and 7.5 mL of methanol (MeOH) using an Agilent 1260 Infinity LC pump, and the eluates were collected separately in two 20-mL amber glass vials. The set-up parameters for EDGE and Agilent for clean-up are provided in Table S2.

50% by weight of the DCM fraction and 50% by weight of MeOH fraction were combined into a 20-mL amber glass vial and evaporated to dryness using the Xcelvap evaporator. The residue was reconstituted with 1 mL MeOH and transferred to 2-mL glass vial and then centrifuged at 4000 rpm for 10 min to sink the visible particles. Afterward, the MeOH extract was filtered through 0.2 μm filter (Whatman, PTFE filter) using a 1-mL disposable syringe. The organic extracts have an enrichment factor ranging from 0.513 to 1.23 $g_{sed,d.w.}/mL_{methanol}$, which are stored at 20-°C until further bioassay, fractionation, and chemical analysis.

Table S1. Sampling information

Sampling site	Latitude	Longitude	Sample name	Total organic carbon content (TOC, %)	Enrichment factor of extract ($g_{sed,dw}/mL_{methanol}$)
Hafen Prossen	50.925	14.127	Prossen	4.1	1.23
Hafen Dresden Neustadt	51.066	13.730	Dresden	5.6	0.714
Hafen Riesa	51.320	13.288	Riesa	4.3	1.16
Hafen Torgau	51.554	13.010	Torgau	5.5	0.907
Hafen Wittenberg	51.864	12.618	Wittenberg	7.8	0.639
Hafen Roßlau	51.856	12.224	Dessau	7.6	0.655
Magdeburg Winterhafen	52.131	11.650	Magdeburg	5.6	0.924
Schleusenkanal Havelberg	52.839	12.045	Werben	5.5	0.902
Hafen Dömitz	53.138	11.260	Domitz	9.8	0.513
Lauenburg rechts	53.369	10.548	Lauenburg	5.0	1.022
/	/	/	Extraction Blank 1	/	1
/	/	/	Extraction Blank 2	/	1

Table S2. Parameters for sample extraction

Automated Solvent Extraction System (EDGE) Used in Pressurized Liquid Extraction	
Pressure	1500 psi (103 bar)
Temprature	100°C
Preheat	5 min
Static time	5 min
Flush volume	60%
Purge time	60 s
Cycles	2
**Agilent 1200 Pump Used in Sample Clean-Up**	
Time	Flow rate
0 – 0.5 min	0 – 5 mL/min
0.5 – 2 min	5 mL/min
2 – 2.1 min	5 – 0 mL/min

Text S2. High-resolution chromatographic fractionation

The organic extract was fractionated by high-performance liquid chromatography (Vanquish, Thermo Scientific) using a reverse-phase C18 column (Waters Acquity UPLC BEH C18 1.7 μm, 2.1 × 100 mm). The chromatography column was pre-equilibrated at initial conditions for 30 min, and washed using 70/30 MeOH/water for 40 min after each batch of measurement. A 10 μL aliquot of the methanolic sediment extract was injected and eluted through a three-Eluent gradient: Eluent A (HPLC-grade water with 0.1% formic acid), Eluent B (methanol with 0.1% formic acid), and Eluent C (acetonitrile). Initially, 97% Eluent A and 3% Eluent B were held for 2 min, followed by a linear increase of Eluent B to 100% from 2 to 16 min, maintained for 2 min. Eluent C was then ramped to 100% over 2 min and held for 4 min. Fractionation lasted 24 min at 0.3 mL/min, after which the flow was diverted to waste. The column was re-equilibrated with 97% Eluent A and 3% Eluent B for 4.5 min at 0.35 mL/min.

Fractions No. 1–52 were eluted from eluent A and B. Fractions No. 53–59 were eluted by eluent B. Fractions No. 60–66 were eluted by phases B and C, while fractions No. 67–78 were eluted by phase C.

Table S3. Parameters for FractioMate collector

FractioMate (version 2.0.0.21, T4S-VU Amsterdam)	
Spotting on	96 HIGH profile at Rear Plate position on LEFT
Plate height	20 mm (including tray carrier)
Spotter needle	@ 24 mm above tray carrier
Repeat cycle	1 of 1
Interval time	18 seconds
Temperature	26.5 °C

Text S3. AhR CALUX Bioassay

Mixture experiment

To test the hypothesis that chemicals in sediment organic extracts cause AhR activation effects in a concentration addition manner. Sample extracts were mixed either by equal volume or varying volumes to induce the same AhR activation effect, hereafter termed as equal potency. Specifically, the equivolume mixture was prepared by mixing 100 µL or 50 µL aliquots from each sediment extract after reconstitution with cell medium (*n* = 10) with a REF of 0.0347 $g_{sed,dw}/mL_{bioassay\ medium}$. The equipotent mixture was prepared by mixing varying volumes of methanolic sediment extract, blown down to dryness under gently nitrogen flow, and reconstituted in 10 mL cell medium with an REF of 0.02 $g_{sed,dw}/mL_{bioassay\ medium}$. The volume from each organic extract was calculated based on the estimated effect concentration assuming concentration addition. The bioassay was performed for both mixtures as described above.

Bioassay testing of fractions

To qualitatively evaluate the AhR activation effect caused by the fractions, the bioassay was conducted at a constant REF ranging from 1.28 $\times10^{-2}$ to 3.08 $\times10^{-2}$ $g_{sed,dw}/mL_{bioassay\ medium}$. The 96-well plates coming from the FractioMate were placed at room temperature, and reconstituted with 100 µL cell medium. After shaking at 800 rpm for 1 h at room temperature, 10 µL from each well in 96-well plate was transferred to 384-well plate containing 30 µL cell medium per well that had been pre-incubated for 24 h for dosing. Moreover, bioassays were conducted on pooled fractions No. 1–52, No. 53–80, and No. 1–80 as described above. Specifically, 10 µL from each well in the 96-well plate were pooled after reconstitution in 100 µL of cell medium.

Bioassay of identified chemicals

A total of 30 detected chemicals were selected for bioanalytical confirmation based on their structure diversity (similarity group), abundance, and commercial availability. Details see Table S4. Stock solutions in methanol or ethyl acetate were prepared for each chemical in a 10-mL amber glass vial with aluminum cap containing PTFE septum. The vials were placed in a shaker at 700 rpm overnight to ensure the solid chemicals were fully dissolved. Aliquots of stock solution of reference standards were placed in 1.5 mL clear glass vials with inner cone, evaporated to completely dryness using a Barkey evaporator under gently nitrogen flow, and then reconstituted with cell medium to achieve around their solubility in cell medium.[2] Before dosing into cell plate, the vials containing cell medium constitution were shaken on a bioshaker at 1000 rpm and 37°C overnight for 24 h.

Table S4. Reference standards for bioanalytical and chemical confirmation

No.	Chemicals	CASRN	Molecular Formula	Molecular Weight (g/mol)	Monoisotopic Mass (g/mol)	Supplier	Purity	For bioanalytical confirmation	For analytical confirmation
1	Phenanthrene	85-01-8	C14H10	178.234	178.0783	Sigma Aldrich	98%	×	×
2	Anthracene	120-12-7	C14H10	178.234	178.0783	Merck	≥96% (GC)	×	×
3	Fluoranthene	206-44-0	C16H10	202.256	202.0783	Sigma-Aldrich	98%	×	×
4	Pyrene	129-00-0	C16H10	202.256	202.0783	abcr	98%	×	×
5	Chrysene	218-01-9	C18H12	228.294	228.0939	Sigma Aldrich	99.9% (GC)	×	×
6	Benz(a)anthracene	56-55-3	C18H12	228.294	228.0939	Sigma Aldrich	99%	×	×
7	Benzo(b)fluoranthene	205-99-2	C20H12	252.316	252.0939	Supelco	100.0%	×	×
8	Benzo[j]fluoranthene	205-82-3	C20H12	252.316	252.0939	JRC	99.5%	×	×
9	Benzo[k]fluoranthene	207-08-9	C20H12	252.316	252.0939	Supelco	99.5%	×	×
10	Benzo[a]pyrene	50-32-8	C20H12	252.316	252.0939	Sigma-Aldrich	≥96% (HPLC)	×	×
11	Benzo[e]pyrene	192-97-2	C20H12	252.316	252.0939	Aldrich	99%	×	×
12	Perylene	198-55-0	C20H12	252.316	252.0939	Merck	≥98.0% (HPLC)	×	×
13	Anthanthrene	191-26-4	C22H12	276.338	276.0939	JRC	99.5%	×	×
14	2-Methylphenanthrene	2531-84-2	C15H12	192.261	192.0939	LGC	>95% (HPLC)	×	×
15	2-Methylanthracene	613-12-7	C15H12	192.261	192.0939	Sigma Aldrich	97%	×	×
16	Retene	483-65-8	C18H18	234.342	234.1409	Targetmol	99%	×	×
17	1-Methylchrysene	3351-28-8	C19H14	242.321	242.1096	JRC	>99%	×	×
18	Benzo[b]naphtho[1,2-d]thiophene	205-43-6	C16H10S	234.32	234.0503	JRC	99.7%	×	×
19	Benzo[b]naphtho[2,1-d]thiophene	239-35-0	C16H10S	234.32	234.0503	Dr. Ehrenstorfer	99.6%	×	×
20	Benzo[b]naphtho[1,2-d]furan	205-39-0	C16H10O	218.255	218.0732	JRC	99.7%	×	×
21	Benzo[b]naphtho[2,3-d]furan	243-42-5	C16H10O	218.255	218.0732	Sigma-Aldrich	99.7%	×	×
22	9,10-Anthracenedione	84-65-1	C14H8O2	208.216	208.0524	Fluka	≥99% (HPLC)	×	×
23	9,10-Anthracenedione, 2-methyl-	84-54-8	C15H10O2	222.243	222.0681	TCI	>99% (GC)	×	×

24	7H-Benzo[c]carbazole	205-25-4	C16H11N	217.271	217.0891	BLD Pharma	97%	×	×
25	Benz[c]acridine	225-51-4	C17H11N	229.282	229.0891	GFT - Aromaten	nd	×	×
26	11H-Benzo[b]fluorene	243-17-4	C17H12	216.283	216.0939	GFT - Aromaten	nd	×	×
27	3,3',4,4',5-Pentachlorobiphenyl (PCB 126)	57465-28-8	C12H5Cl5	326.42	323.8834	Promochem	99.5%	×	×
28	2,2',3,4,4',5'-Hexachlorobiphenyl (PCB 138)	35065-28-2	C12H4Cl6	360.86	357.8444	Dr. Ehrenstorfer	99%	×	×
29	2,2',4,4',5,5'-Hexachlorobiphenyl (PCB 153)	35065-27-1	C12H4Cl6	360.86	357.8444	Promochem	>99%	×	×
30	Decachlorobiphenyl (PCB 209)	2051-24-3	C12Cl10	498.63	493.6885	Dr. Ehrenstorfer	96%	×	×
31	Benzenamine, 3,4-dichloro-	95-76-1	C6H5Cl2N	162.01	160.9799	Sigma-Aldrich	98%		×
32	1-methylphenanthrene	832-69-9	C15H12	192.261	192.0939	Sigma-Aldrich	98%	×	×
33	1-methylanthracene	610-48-0	C15H12	192.261	192.0939	Sigma-Aldrich	98%	×	×
34	1-methylpyrene	2381-21-7	C17H12	216.283	216.0939	Sigma-Aldrich	98%		×

Text S4. Concentration-response curve evaluation

The 4-parameter log-logistic equation was first used to estimate the maximum luciferase activity (relative light units (RLU (max)) for the reference compound TCDD. The RLU (min) was the RLU of the unexposed cells. The AhR activation was calculated from the RLU of each well using the equation (1).

$$\text{AhR activation} = (\text{RLU-RLU(min)})/(\text{RLU(max)-RLU(min)}) \quad (1)$$

where RLU represent the detected luciferase activity in a plate well; RLU (min) represents the detected luciferase activity for unexposed cells; and RLU (max) represents the estimated TOP value in the 4-parameter hill equation for the reference standard.

The cytotoxicity caused by the exposure to chemicals or sediment extracts was evaluated by the growth inhibition after 24 h exposure using the equation (2).

$$\text{Cytotoxicity} = \frac{\text{Confluency (sample)}}{\text{Confluency (average unexposed cell)}} \quad (2)$$

The nominal concentration in the 384-cell plate at which cytotoxicity reaches absolute 10% (IC_{10}) is estimated using the equation (3) by linear regression between dosing concentration and cytotoxicity.

$$IC_{10}=\frac{10}{\text{slope}} \quad (3)$$

EC_{10}, the nominal concentration at which the chemical exerts absolute 10% of the maximum effect, was used to quantify the AhR-activation of chemicals or mixtures in this study. The concentration response curves (CRC) are typically linear up to 30% of the maximum effect and only concentrations below IC_{10} were used for the derivation of the EC_{10}.[3] EC_{10} was then calculated using equation (4) by the linear regression between concentration in the well and AhR activation.

$$EC_{10}=\frac{10}{\text{slope}} \quad (4)$$

The specificity of AhR activation effects was evaluated by calculating the specificity ratio ($SR_{cytotoxicity}$) value with equation (5). AhR activation effects caused by chemicals with a $SR_{cytotoxicity}$ higher than 10 is considered to be a specific effect, between 1 and 10 moderately specific, SR <1 is an artifact caused by cytotoxicity burst and not reported as active. For extracts containing complex mixtures of chemicals, specific and nonspecific effects overlap, and we consider $SR_{cytotoxicity}$ > 1 as specific, and do not report if specific effects were masked by cytotoxicity, which would equate to SR < 1.

$$SR_{cytotoxicity}=\frac{IC_{10}}{EC_{10}} \quad (5)$$

Text S5. Machine learning model training

The concentration-response curves (CRCs) for 8116 chemical substances from the Tox21 dataset (TOX21_AhR_LUC_Agonist) were re-evaluated to reclassify activity (the threshold was set at efficacy > 10% of reference compound and a good linear fit) and derive EC_{10} values. Confidence values ranging from 0 to 1 were assigned to each chemical as described in https://git.ufz.de/braung/automatedbioassayscreening.

Briefly, four models, including constant model, gain-loss model, and hill-model for log-logistic fits as well as linear regression were fit to the concentration-response data for each chemical compound in the Tox21 dataset. Akaike information criterion was used as reference for best fit with a minimum absolute difference of 3 to the constant model. Coefficient of determination for linear regression and Cox-Snell pseudo R2 were used as a value of goodness of fit. The distance of individual data points from the fit was used to identify and remove outliers via the Rosner test and the fit was applicable when applicable when at least five independent values define the curve. A confidence value of the fit was calculated based on the standard error of the activity concentration (EC_{10} or IC_{10}), a confidence factor, and the coefficient of determination the regression model as described in equation (6).

$$\text{confidence}_{\text{CRC}}=\left(1-\left|\frac{SE_{\frac{EC_{10}}{IC_{10}}}}{\frac{EC_{10}}{IC_{10}}}\right|\right)*\text{factor}*R^2 \tag{6}$$

The confidence factor was set to 0.5 if the number of concentration-response pairs below or above the activity threshold of 10% was below 2, otherwise the factor was set to 1. Chemical substance was classified as an AhR agonist (active) with a confidence value of ≥ 0.2, inconclusive with a value between 0 and 0.2, and inactive if the value was null. The EC_{10} value (µM) was estimated from their CRC if the confidence level was ≥ 0.4.

After removing duplicates in the re-evaluated Tox 21 dataset, 6,925 chemicals were curated. Limiting the accurate mass to 50-800 and logK_{ow} (octanol-water partition constant) values from -2 to 8 resulted in 6,766 molecular structures for descriptor calculation, of which 4,946 inactive, 1,025 inconclusive, and 795 actives. Molecular structures classified as inactive and active were kept for the training dataset for binary classification model. Molecular structures classified as active with confidence value ≥ 0.7 were kept for the training dataset for regression model.

The eXtreme gradient boosting, XGBoost, was used in this study to train both the binary classification model (XGBClassifier, version 3.0.2) and regression model (XGBregressor, version 2.1.4). XGBoost is a decision tree-based model architecture and uses ensemble learning techniques to provide consensus prediction. The dataset was split 80/20 into training and test pools using stratified sampling. Each molecule was represented by a 529-dimensional vector of molecular descriptors, with outputs as

probabilities between 0 and 1. To address class imbalance, positive samples were assigned higher weights proportional to the negative-to-positive ratio. Early stopping was applied after 8 training iterations to prevent overfitting. The hyperparameters were tuned to optimize the classification prediction via 5-fold cross-validation. Briefly, after several round of manual tuning, finally we chose max depth, min child weight, subsample, and num parallel tree for hyperparameter tuning to enhance the classification model performance. The hyperparameters were tuned using grid search method within a defined range to search the best parameter combination using the evaluation metric of 'neg_log_loss'. The model tuning was operated using the same training dataset with 5-fold cross validation.

Model performance for the binary classification was evaluated by precision, recall, and F1 score, calculated by equations (7)–(9) on both training and test data pools.

$$\text{Precision}=\frac{TP}{TP+FP} \tag{7}$$

$$\text{Recall}=\frac{TP}{TP+FN} \tag{8}$$

$$\text{F1 score}=\frac{2\times\text{Precision}\times\text{Recall}}{\text{Precision}+\text{Recall}} \tag{9}$$

Precision measures the proportion of true positives (TP) among all predicted positives, assessing prediction validity. Recall measures the proportion of true positives among all actual positives, evaluating prediction completeness. The F1 score harmonizes precision and recall. The area under the receiver operating characteristic curve and the area under the precision-recall curve were calculated for evaluation. The precision-recall curve was used to select a threshold that balances precision and recall, especially for imbalanced training data. The classification threshold was set at 0.446; values above this were labeled positive (AhR active = 1), and those below negative (AhR inactive = 0).

The EC_{10} values for chemical compounds in the Tox21 dataset were included in training the regression model if their confidence level was ≥ 0.7. Most values exceeded 1 μM, indicating low potency as AhR agonists. Therefore, we included all PAHs confirmed by the AhR CALUX bioassay and used their estimated EC_{10} values to expand the regression model's application domain. The dataset was split 80/20 into training and test pools. Each molecule was represented by a 529-dimensional vector of molecular descriptors. Early stopping was applied after 8 training iterations without loss improvement to prevent overfitting. The regularization was also applied to prevent overfitting. The model performance for the regression was evaluated by the root mean square error (RMSE). The evaluation metrics were calculated using the packages from the scikit-learn python library.

Table S5. Added dioxins and dioxin-like compounds in classification training dataset

Chemicals	Preferred name	CASRN	InChIKey	CIDs	AhR activity	Chemical group
2,3,7,8-Tetrachlorodibenzo-1,4-dioxin	2,3,7,8-TCDD	1746-01-6	HGUFODBRKLSHSI-UHFFFAOYSA-N	15625	Active	Chlorinated dibenzo-p-dioxins
1,2,3,7,8-Pentachlorodibenzo-p-dioxin	1,2,3,7,8-PeCDD	40321-76-4	FSPZPQQWDODWAU-UHFFFAOYSA-N	38439	Active	Chlorinated dibenzo-p-dioxins
1,2,3,4,7,8-Hexachlorodibenzodioxin	1,2,3,4,7,8-HxCDD	39227-28-6	WCYYQNSQJHPVMG-UHFFFAOYSA-N	38251	Active	Chlorinated dibenzo-p-dioxins
1,2,3,6,7,8-Hexachlorodibenzo-p-dioxin	1,2,3,6,7,8-HxCDD	57653-85-7	YCLUIPQDHHPDJJ-UHFFFAOYSA-N	42540	Active	Chlorinated dibenzo-p-dioxins
1,2,3,7,8,9-Hexachlorodibenzo-p-dioxin	1,2,3,7,8,9-HxCDD	19408-74-3	LGIRBUBHIWTVCK-UHFFFAOYSA-N	29575	Active	Chlorinated dibenzo-p-dioxins
1,2,3,4,6,7,8-Heptachlorodibenzodioxin	1,2,3,4,6,7,8-HpCDD	35822-46-9	WCLNVRQZUKYVAI-UHFFFAOYSA-N	37270	Active	Chlorinated dibenzo-p-dioxins
Octachlorodibenzo-p-dioxin	OCDD	3268-87-9	FOIBFBMSLDGNHL-UHFFFAOYSA-N	18636	Active	Chlorinated dibenzo-p-dioxins
2,3,7,8-Tetrachlorodibenzofuran	2,3,7,8-TCDF	51207-31-9	KSMVNVHUTQZITP-UHFFFAOYSA-N	39929	Active	Chlorinated dibenzofurans
1,2,3,7,8-Pentachlorodibenzofuran	1,2,3,7,8-PeCDF	57117-41-6	SBMIVUVRFPGOEB-UHFFFAOYSA-N	42138	Active	Chlorinated dibenzofurans
2,3,4,7,8-Pentachlorodibenzofuran	2,3,4,7,8-PeCDF	57117-31-4	OGBQILNBLMPPDP-UHFFFAOYSA-N	42128	Active	Chlorinated dibenzofurans
1,2,3,4,7,8-Hexachlorodibenzofuran	1,2,3,4,7,8-HxCDF	70648-26-9	LVYBAQIVPKCOEE-UHFFFAOYSA-N	51130	Active	Chlorinated dibenzofurans
1,2,3,6,7,8-Hexachlorodibenzofuran	1,2,3,6,7,8-HxCDF	57117-44-9	JEYJJJXOFWNEHN-UHFFFAOYSA-N	42140	Active	Chlorinated dibenzofurans
1,2,3,7,8,9-Hexachlorodibenzofuran	1,2,3,7,8,9-HxCDF	72918-21-9	PYUSJFJVDVSXIU-UHFFFAOYSA-N	51720	Active	Chlorinated dibenzofurans
2,3,4,6,7,8-Hexachlorodibenzofuran	2,3,4,6,7,8-HxCDF	60851-34-5	XTAHLACQOVXINQ-UHFFFAOYSA-N	43495	Active	Chlorinated dibenzofurans
1,2,3,4,6,7,8-Heptachlorodibenzo[b,d]furan	1,2,3,4,6,7,8-HpCDF	67562-39-4	WDMKCPIVJOGHBF-UHFFFAOYSA-N	38199	Active	Chlorinated dibenzofurans
1,2,3,4,7,8,9-Heptachlorodibenzofuran	1,2,3,4,7,8,9-HpCDF	55673-89-7	VEZCTZWLJYWARH-UHFFFAOYSA-N	41510	Active	Chlorinated dibenzofurans
Octachlorodibenzofuran	OCDF	39001-02-0	RHIROFAGUQOFLU-UHFFFAOYSA-N	38200	Active	Chlorinated dibenzofurans
3,3',4,4'-Tetrachlorobiphenyl	PCB77	32598-13-3	UQMGJOKDKOLIDP-UHFFFAOYSA-N	36187	Active	Non-ortho–substituted PCBs
3,4,4',5-Tetrachlorobiphenyl	PCB81	70362-50-4	BHWVLZJTVIYLIV-UHFFFAOYSA-N	51043	Active	Non-ortho–substituted PCBs
3,3',4,4',5-Pentachlorobiphenyl	PCB126	57465-28-8	REHONNLQRWTIFF-UHFFFAOYSA-N	63090	Active	Non-ortho–substituted PCBs

3,3',4,4',5,5'-Hexachlorobiphenyl	PCB169	32774-16-6	ZHLICBPIXDOFFG-UHFFFAOYSA-N	36231	Active	Non-ortho–substituted PCBs
2,3,3',4,4'-Pentachlorobiphenyl	PCB105	32598-14-4	WIDHRBRBACOVOY-UHFFFAOYSA-N	36188	Active	Mono-ortho–substituted PCBs
2,3,4,4',5-Pentachlorobiphenyl	PCB114	74472-37-0	SXZSFWHOSHAKMN-UHFFFAOYSA-N	53036	Active	Mono-ortho–substituted PCBs
2,3',4,4',5-Pentachlorobiphenyl	PCB118	31508-00-6	IUTPYMGCWINGEY-UHFFFAOYSA-N	35823	Active	Mono-ortho–substituted PCBs
2,3',4,4',5'-Pentachlorobiphenyl	PCB123	65510-44-3	YAHNWSSFXMVPOU-UHFFFAOYSA-N	47650	Active	Mono-ortho–substituted PCBs
2,3,3',4,4',5-Hexachlorobiphenyl	PCB156	38380-08-4	LCXMEXLGMKFLQO-UHFFFAOYSA-N	38019	Active	Mono-ortho–substituted PCBs
2,3,3',4,4',5'-Hexachlorobiphenyl	PCB157	69782-90-7	YTWXDQVNPCIEOX-UHFFFAOYSA-N	50891	Active	Mono-ortho–substituted PCBs
2,3',4,4',5,5'-Hexachlorobiphenyl	PCB167	52663-72-6	AZXHAWRMEPZSSV-UHFFFAOYSA-N	40479	Active	Mono-ortho–substituted PCBs
2,3,3',4,4',5,5'-Heptachlorobiphenyl	PCB189	39635-31-9	XUAWBXBYHDRROL-UHFFFAOYSA-N	38306	Active	Mono-ortho–substituted PCBs

The above data are from literature https://doi.org:10.1093/toxsci/kfl055

Text S6. Chemical analysis

A 5 µL aliquot from sediment extract was injected in splitless mode into thermodesorption tubes with glass inserts, treated as single-use liners. The TDU3 was held at 80°C for 4 minutes, then ramped at 720°C/min to 300°C to transfer analytes into the GC's cold injection system at 10°C. After 5 minutes, the GC injector was heated at 12°C/s to 300°C and held for 10 minutes. A DB-5MS capillary column (30 m × 250 µm 0,25 µm; Agilent) was used for chromatographic separation. Helium was used as carrier gas at a constant flow rate of 1.2 mL/min. The oven was kept at 60°C for 1 min, heated at 12.6°C/min to 300°C from 1 min to 20 min and held for 10 min. The temperature of transfer line was 250°C. Analytes were ionized by electron ionization at 70 eV at a source temperature of 200°C. Data were acquired in full scan mode at m/z (m/z 60–810) at a resolving power of 60,000 (referenced to m/z 200). To maintain the mass spectrometry at its best performance, the ion source was thoroughly cleaned and the orbitrap was tuned and mass calibrated before each sequence. The mass error was kept within 5 ppm, respectively.

Table S6. Parameters for GC-Orbitrap

Full scan MS method used in the QExactive GC-Orbitrap	
Method duration	30 min
Filament on delay	3 min
MS transfer line temperature	250°C
Ion source temperature	200°C
Ionization mode	EI
Polarity	positive
Resolution	60000
AGC target	1.00E+06
Maximum IT	auto
Scan range	60 to 810 m/z
Spectrum data type	Centroid

Text S7. Suspect screening analysis

A suspect screening analysis workflow was established (Figure S8). MS-DIAL software (ver. 4.9.221218, https://zenodo.org/records/12589462) with a GUI was used for peak picking, deconvolution, alignment, gap filling, and retention index calculation from Thermo Fisher Xcalibur (RAW) centroid data of raw extracts. The parameters are provided in Table S7. Briefly, the retention time was set from 0 to 30 min, with accurate mass ranging from 50 to 800 for peak detection. Peak was detected using the linear Weighted Moving Average method, and extracted ion chromatograph was constructed from total ion chromatograph using the model ion (either the molecular ion or base ion). The retention index (RI) for each detected feature was calculated by the MS-DIAL software based on Kovats method (equation 10). This was done by uploading a txt file containing manually identified n-alkanes (base peak or quant mass at m/z = 71.0856) with carbon numbers from 10 to 36, derived from a 1 ppm alkane mix standard (Sigma-Aldrich 49452-U).

$$I_x=100\times n+100\times \frac{\log(t_x)-\log(t_n)}{\log(t_{n+1})-\log(t_n)} \quad (10)$$

where I_x is the Kovats retention index of a compound; t_x is the retention time for a compound; n is the carbon number of n-alkane hydrocarbon that just elutes before the compound. t_n and t_{n+1} are retention times of the reference n-alkane hydrocarbons eluting immediately before and after chemical compound.

Features with a peak height fold-change (sample average divided by extraction blank average) greater than 5 were removed. The peak list for each sample as well as extraction blank samples was exported in csv and msp data format. Detected GC-HRMS features were pre-filtered by signal-to-noise ≥ 10 and deconvoluted ion area ≥ 10^5. The low abundance features normally have odd-peak shape and messy deconvoluted mass spectra.

The filtered GC-HRMS features in msp data format were identified by matching the mass spectra against the NIST 23 library using NIST MS Search Program (3.0). Briefly, there are a total of 394,054 EI mass spectra for 347,100 chemical compounds in the NIST 23 library (https://www.nist.gov/srd/nist-standard-reference-database-1a). Each HRMS feature was identified by matching the measured mass spectra (two-dimensional matrix containing m/z and intensity) with the library. The similarity was evaluated (scored) by the

build-in full spectrum search algorithm. The top ranking 10 candidates were retrieved for each HRMS feature. Next, the top-ranking candidates were filtered out if the RI tolerance, calculated as the absolute value of RI for candidate from the library minus calculated RI for the features, > 200. The remaining features were filtered by either the Match Factor (mf) > 700 or the mf < 700 but the Reverse Match Factor (rmf) >700.[4] The identifier for each candidate was then curated by retrieving InChIKey from the NIST library and CIDs from the PubChem database.

The established machine learning models were applied to predict the AhR activation of each candidate and its EC_{10} value if they were predicted to be active.

To elucidate the molecular structures of feature with definitive library match, the top-ranking candidates with probability values < 4% were filtered out and then features were grouped based on the molecular weight of candidates and quant mass (either molecular ion or base peak in the MS spectra). Two groups were derived: one with a single feature mapped to filtered top-ranking candidates, termed as unique feature; another with multiple features mapped to top-ranking candidate, suggesting they are isomers, termed as isomer group. Groups without candidates predicted to be AhR active were filtered out. The unique features were tentatively identified as the first ranking candidate. The features in the isomer groups were tentatively identified by assigning candidate to each feature according to elution order based on RI.

The Prossen and Wittenberg sediment extracts were used as quality control samples as these two exhibited the highest TCDD-EQ after organic carbon normalization. The features tentatively identified in Prossen and Wittenberg served as a target list for alignment across other samples by a mass error within 0.003 Da (10 ppm referenced to m/z 300) and retention time difference within 0.03 min. Finally, the tentatively identified features were grouped according to their structure similarity. Specifically, the molecular formula was used to distinguish unsubstituted and alkylated PAHs from heterocyclic PAHs, which contain N, S, or O. Subsequently, unsubstituted and alkylated PAHs were separated based on their molecular structures.

Table S7. Parameters for MS-DIAL for HRMS peak picking and deconvolution

MS-DIAL ver. 4.9.221218	
#Data type	
Data type	Centroid
Ion mode	Positive
Accuracy type	IsAccurate
#Data collection parameters	
Retention time begin	0
Retention time end	30
Mass range begin	50
Mass range end	800
#Data processing	
Number of threads	8
#Peak detection parameters	
Smoothing method	LinearWeightedMovingAverage
Smoothing level	3
Average peak width	20
Minimum peak height	5000
Mass slice width	0.05
Mass accuracy	0.025
#MS1Dec parameters	
Sigma window value	0.5
Amplitude cut off	10
#identification	
MSP file	
RI index file	see last
Retention type	RI
RI compound	Alkanes
Retention time tolerance	0.5
Retention index tolerance	20
EI similarity library tolerance	70
Identification score cut off	70
Use retention information for scoring	TRUE
Use retention information for filtering	FALSE
Use quant masses defined in MSP format file	FALSE
#Alignment parameters setting	
Retention time	RI
Retention index tolerance	20

Retention time tolerance	0.075
EI similarity tolerance	70
Retention time factor	0.5
EI similarity factor	0.5
Identification after alignment	FALSE
Gap filling by compulsion	TRUE
Basepeak mz selected as the representative quant mass	FALSE
#Filtering setting	
Peak count filter	0
N% detected in at least one group	0
Remove feature based on peak height fold-change	TRUE
Sample max / blank average	5
Sample average / blank average	5
Keep identified and annotated metabolites	TRUE
Keep removable features and assign the tag for checking	TRUE

Text S8. Method detection limits for quantification

The instrumental limit of detection (LOD) was determined as the lowest concentration with a signal-to-noise ratio (S/N) ≥ 3 in a series of standards used for building external calibration curves in the range of 0.1 – 950 ng/mL acetonitrile. The method detection limit (MDL) was determined according to a previously published US EPA document:[5] a) if none of the method blanks (i.e., field blank) gave numerical results for an individual analyte, MDL was used as the initial point of the calibration curve with a S/N ≥ 3; b) if some (but not all) of the method blanks for an individual analyte gave numerical results, the highest method blank result was used as the MDL; c) if all of the method blanks for an individual analyte gave numerical results, then MDL of the analyte was calculated using equation (11) as follow:

$$MDL = M + t_{(n-1,\ 1-\alpha\ =\ 0.99)} \times S \qquad (11)$$

where *M* is the mean of the method blank values. Negative values were replaced with zero if the mean is negative. $t(n-1, 1-\alpha = 0.99)$ is the Student's *t*-value for the single-tailed 99th percentile *t* statistic. In this study, *t* was set at 31.82 according to the number of a total of 2 extraction blank samples. *S* is the sample standard deviation of the replicate method blank values.

Text S9. Additional information on the mixture experiments

To confirm that AhR agonists in complex mixtures act in a concentration-additive manner and the BEQ calculations are valid, the ten sediment extracts were mixed in two ways and measured in AhR bioassay including, equal volume or varying volumes to achieve equal AhR activation effects, hereafter termed as equipotent. The two mixed sample extracts both showed AhR activation with effects reaching up to 100% relative to the positive control TCDD (Figure S3). There was no statistically significant difference between the measured B[a]P-EQ_{bio} of the designed mixtures and their B[a]P-EQ_{CA} predicted with concentration addition (CA) (Figure S4), which confirms that the AhR agonists in the organic extracts from sediment samples act concentration-additive and likely their individual components do, too.

Text S10. Additional information on PCB contribution to mixture effects

Several PCBs were identified with a CL of 3. PCB 153 and PCB 138 were present in sediment extracts, and they are major components among technical PCB mixtures.[6] The two PCB congeners were tested in the present study with PCB 153 being inactive and PCB 138 active. Their concentrations in sediments were less than 20 ng/$g_{sed,dw}$ based on total ion chromatograms for estimation, which are consistent with previous studies showing PCBs occur at ng/$g_{sed,dw}$ levels in Elbe sediments.[7] The low concentrations of PCBs resulted in low expected contributions to AhR activation effects in sediment extracts. Concerns could arise that potent AhR agonists like TCDD and PCB 126 may contribute to bioactivity despite being present below the detection limit. PCBs had very low method detection limits (MDL = 0.1 ng/mL, Figure S13). Even if PCB 126 was present in a worst case at levels slightly below the MDL, it would contribute to the total effect ranging from 0.7% to 14.1% but does not dominate (Table S35).

References

(1) Kamjunke, N.; Sanders, T. Complete exhaustion of dissolved nutrients in a large lowland river. *Environmental Monitoring and Assessment* **2024**, *196* (7).
(2) Fischer, F. C.; Henneberger, L.; Schlichting, R.; Escher, B. I. How To Improve the Dosing of Chemicals in High-Throughput in Vitro Mammalian Cell Assays. *Chemical Research in Toxicology* **2019**, *32* (8), 1462-1468.
(3) Escher, B.; Braun, G.; Zarfl, C. Exploring the Concepts of Concentration Addition and Independent Action Using a Linear Low-Effect Mixture Model. *Environ Toxicol Chem* **2020**, *39* (12), 2552-2559.
(4) Stein, S. Mass Spectral Reference Libraries: An Ever-Expanding Resource for Chemical Identification. *Analytical Chemistry* **2012**, *84* (17), 7274-7282.
(5) US EPA, in: Definition and procedure for the determination of the method detection limit, revision 2. 2016, 821-R-16-006. https://www.epa.gov/cwa-methods/procedures-detection-and-quantitation-documents.
(6) Safe, S.; Bandiera, S.; Sawyer, T.; Robertson, L.; Safe, L.; Parkinson, A.; Thomas, P. E.; Ryan, D. E.; Reik, L. M.; Levin, W.; et al. PCBs: structure-function relationships and mechanism of action. *Environ Health Perspect* **1985**, *60*, 47-56.
(7) Pham, D. N.; El Toum, S.; Martineau, R.; Heise, S.; Sokolova, I. M. Sediment contamination in two German estuaries: A biomarker-based toxicity test with the ragworm Hediste diversicolor under intermittent oxygenation. *Environmental Research* **2025**, *265*, 120451.

Figures

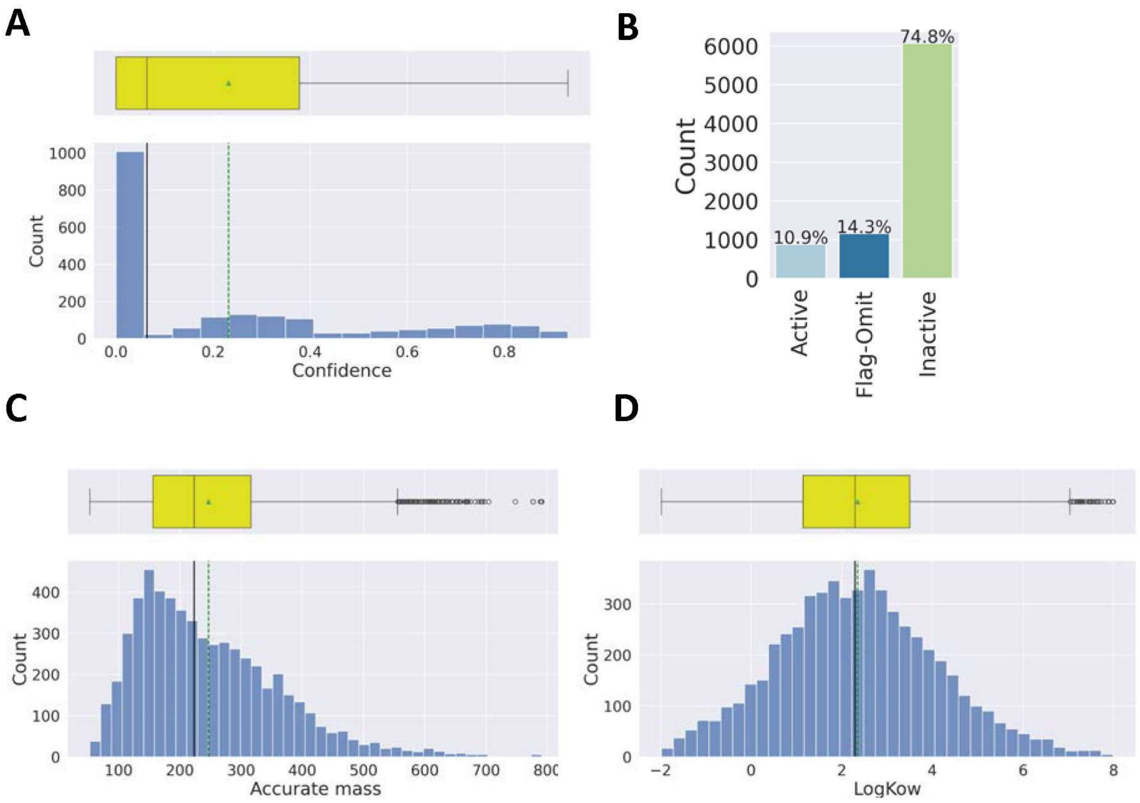

Figure S1. Tox21 dataset characterization after re-evaluation as the training dataset for machine learning-based binary classification model. (A) Confidence value (ranging from 0 to 1, with 1 indicating the highest reliability) assigned to each chemical compound in the Tox21 dataset after evaluating their concentration-response curves; (B) Chemical labels were assigned based on confidence values. Values ranging from 0.2 to 1 indicating AhR-active, with null values indicating AhR-inactive, with values between 0 and 0.2, along with 25 special cases with null values indicating AhR-inconclusive (Flag-Omit); (C) and (D) Distribution of accurate mass and logK_{ow} (octanol-water partition constant).

Prossen

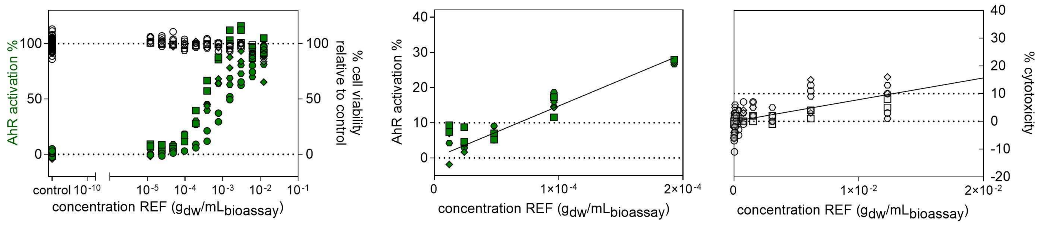

Dresden

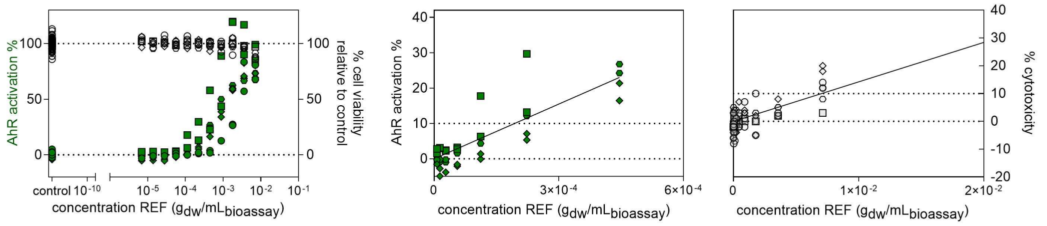

Riesa

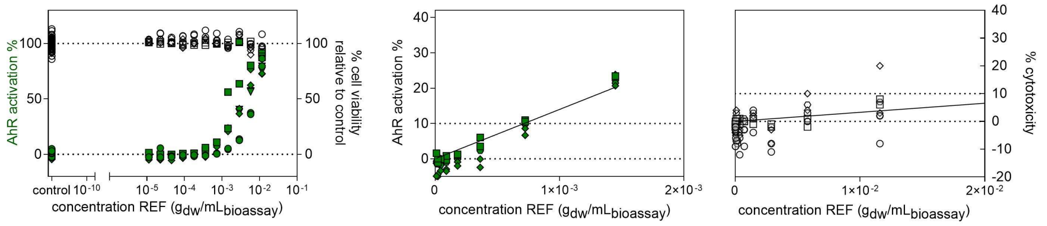

Torgau

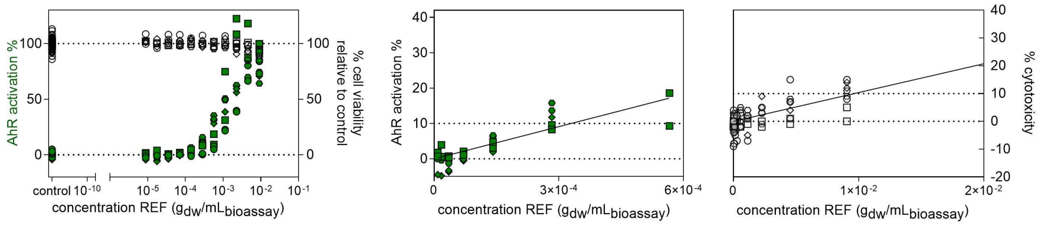

Wittenberg

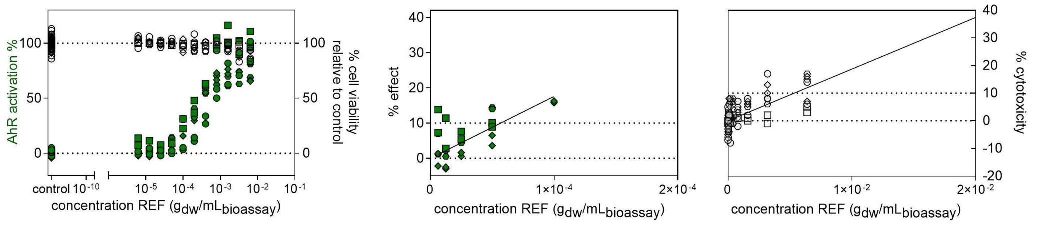

Continue

Dessau

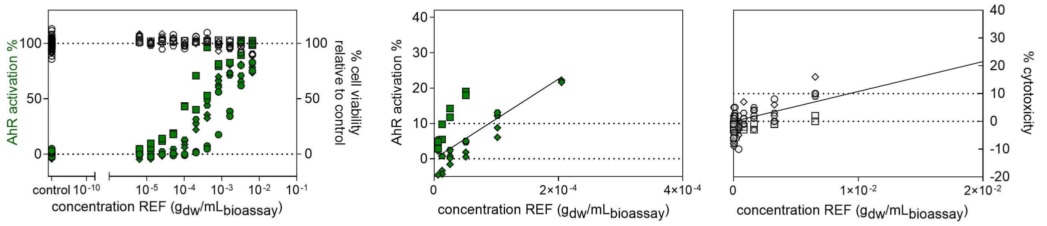

Magdeburg

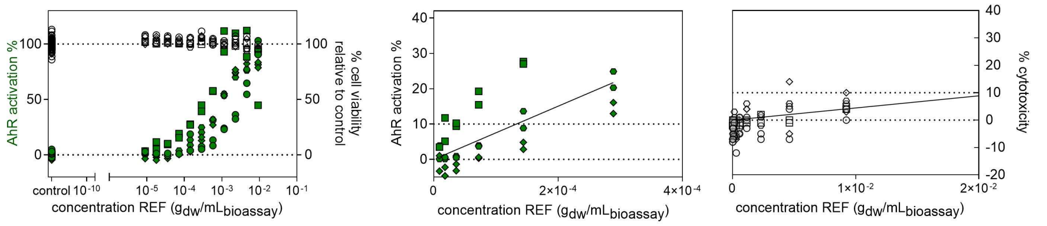

Werben

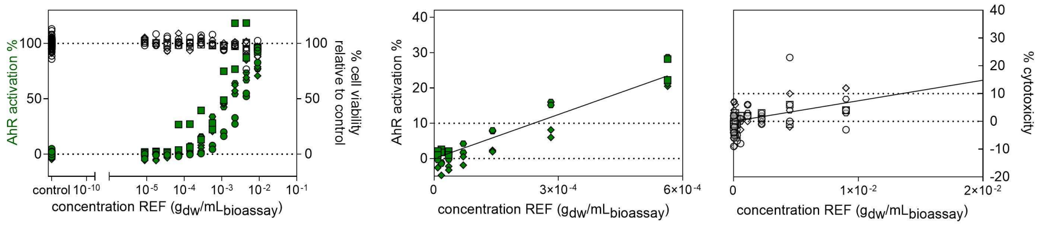

Domitz

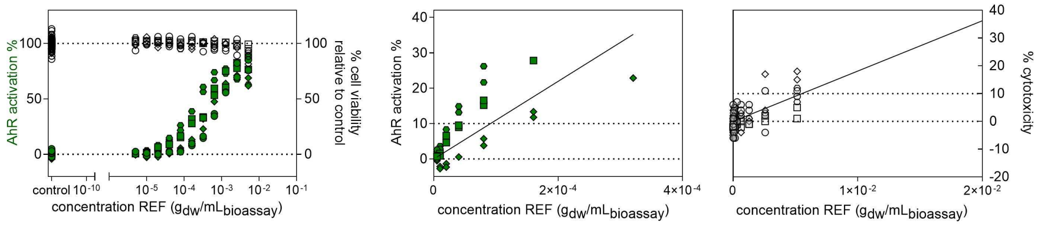

Lauenburg

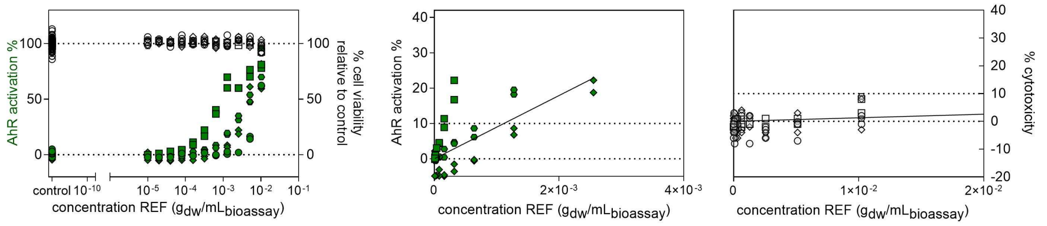

Continue

Extraction Blank1

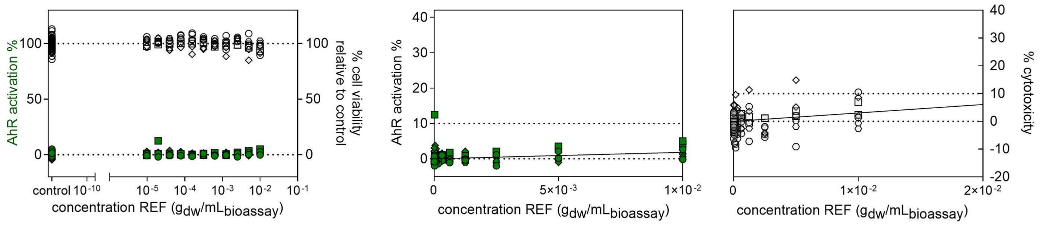

Extraction Blank2

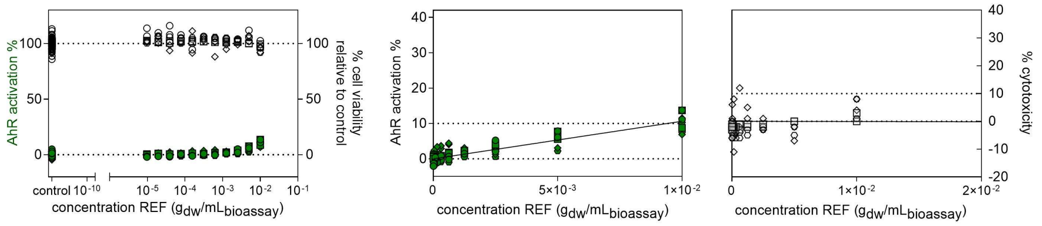

Figure S2. Concentration-response curves of the organic extracts from sediment samples and extraction blank samples in the AhR CALUX bioassay.

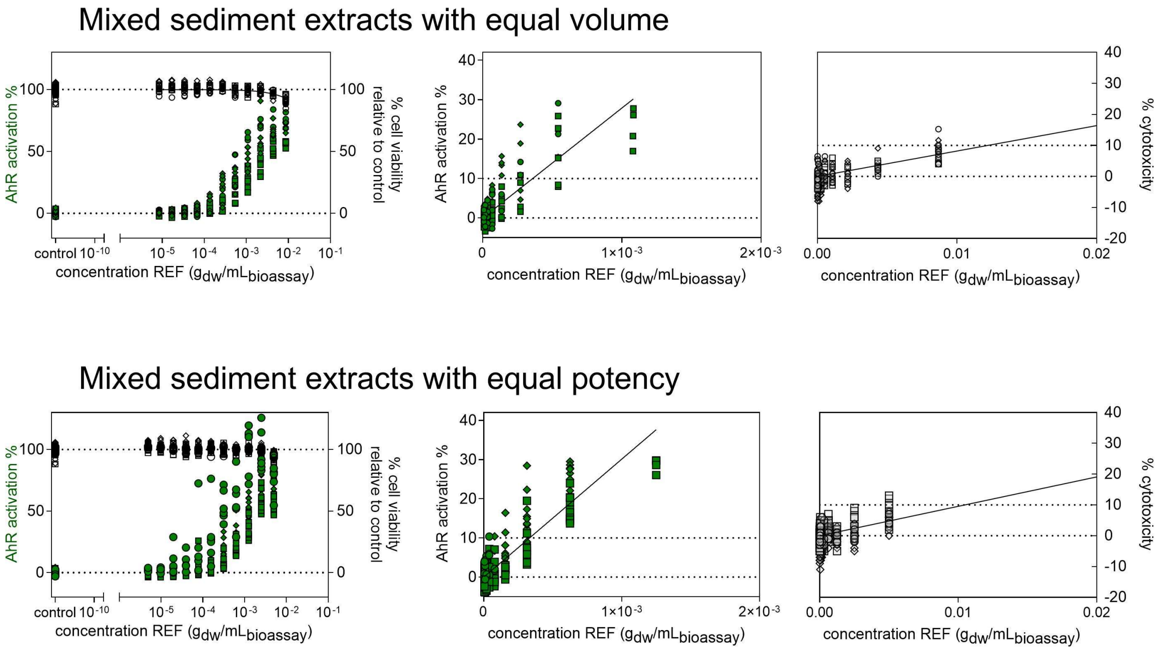

Figure S3. Concentration-response curves of the mixed sediment organic extracts in the AhR CALUX bioassay.

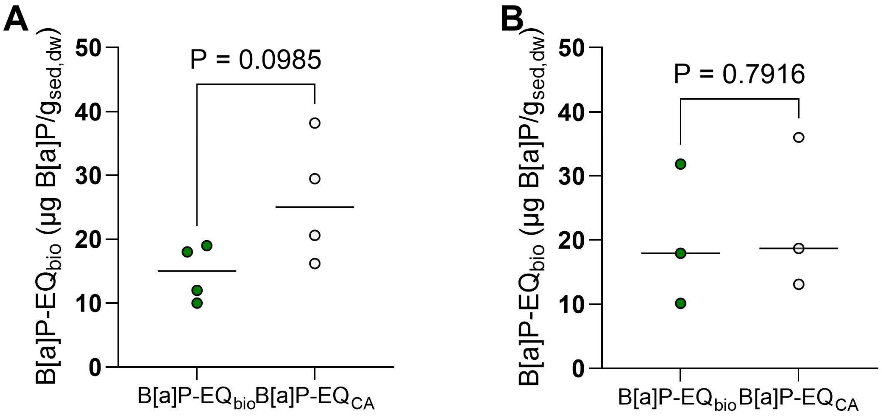

Figure S4. Bioassay results for designed mixtures. (A) B[a]P-EQ_{bio} of mixtures of 10 sample extracts at equal volume ratios ($F = 4.876$, $t = 2.119$, $n = 4$); and (B) B[a]P-EQ_{bio} of mixtures of 10 sample extracts mixed at equipotent concentration ratios ($F = 1.182$, $t = 0.2826$, $n = 3$) compared with the B[a]P-EQ_{CA} predicted with concentration addition (CA). Two-tailed P values were from unpaired Welch's t-tests, with significance at 0.05.

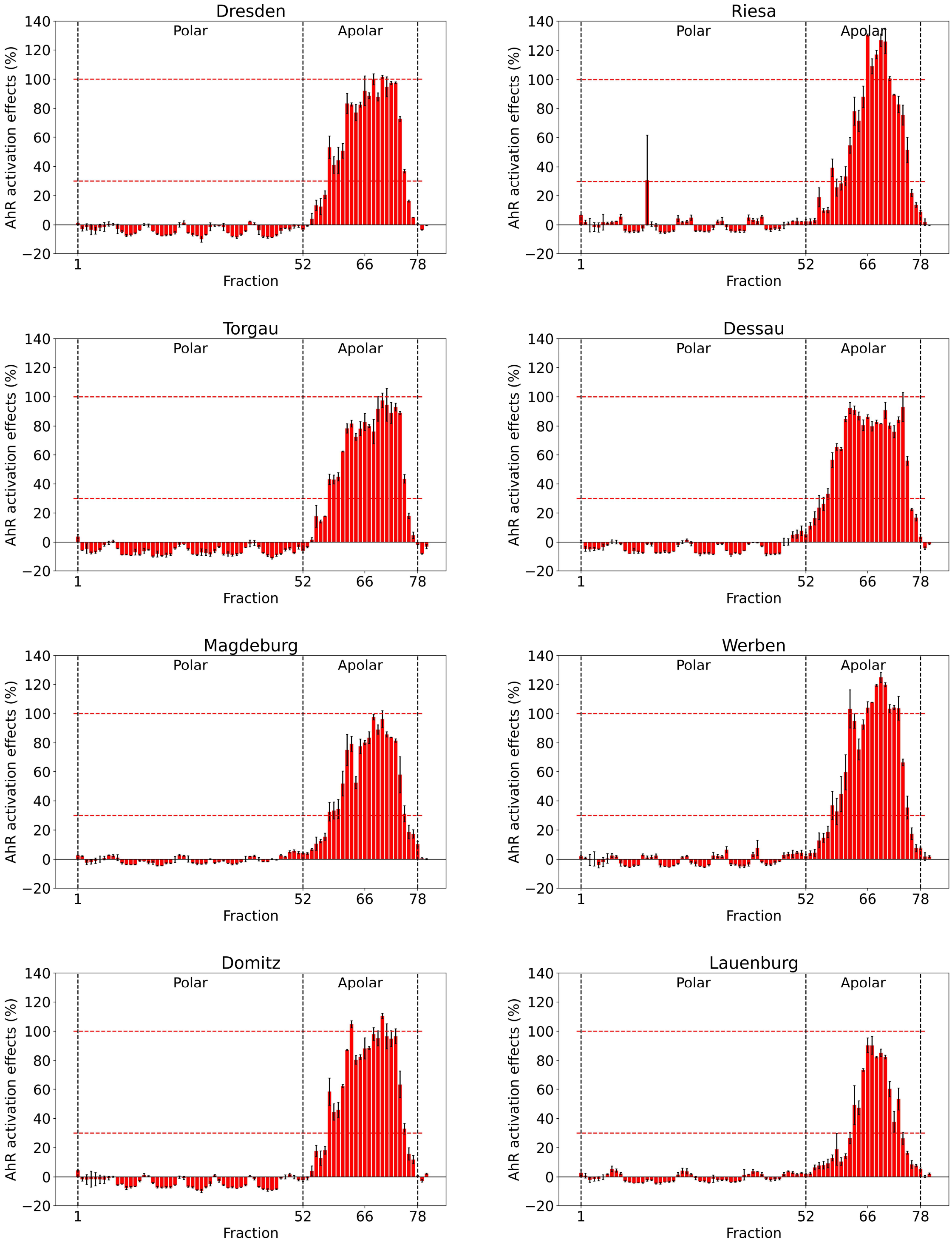
Dresden
Riesa
Torgau
Dessau
Magdeburg
Werben
Domitz
Lauenburg
Polar
Apolar
AhR activation effects (%)
Fraction
140
120
100
80
60
40
20
0
−20
1
52
66
78

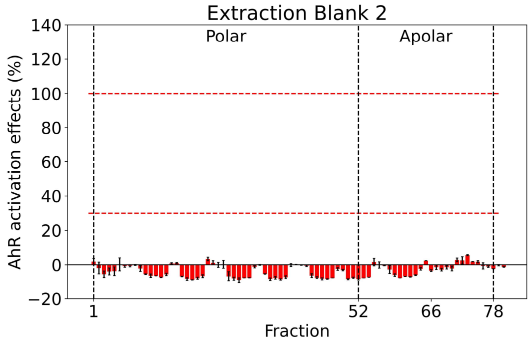

Figure S5. AhR activation effects for sediment extract fractions from high-resolution fractionation. The effect was relative to TCDD and each fraction was at constant relative enrichment factors (REF) per sample varying from 1.28×10^{-2} to 3.08×10^{-2} $g_{sed,dw}/mL_{bioassay\ medium}$ for different sediment samples.

Prossen pooled fractions No.1-80

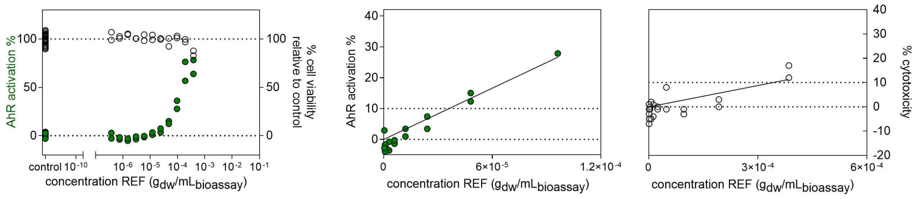

Prossen pooled fractions No.1-52

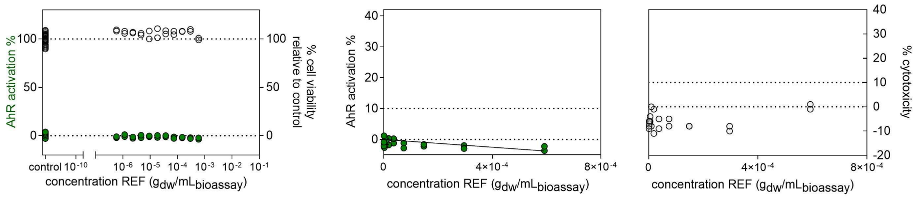

Prossen pooled fractions No.53-80

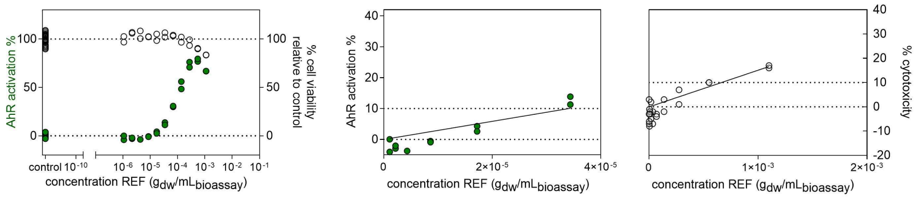

Dresden pooled fractions No.1-80

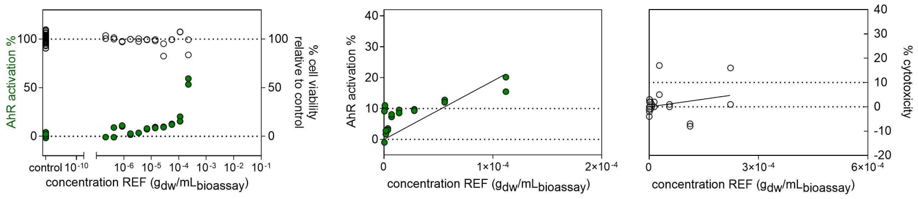

Dresden pooled fractions No.1-52

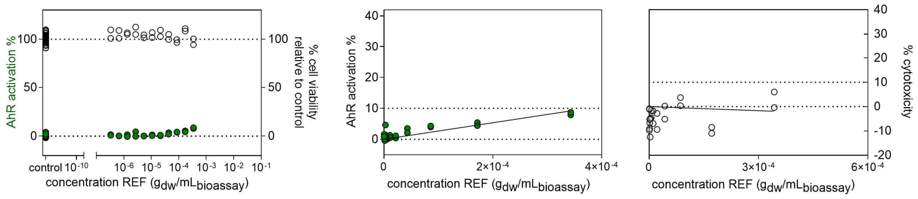

Dresden pooled fractions No.53-80

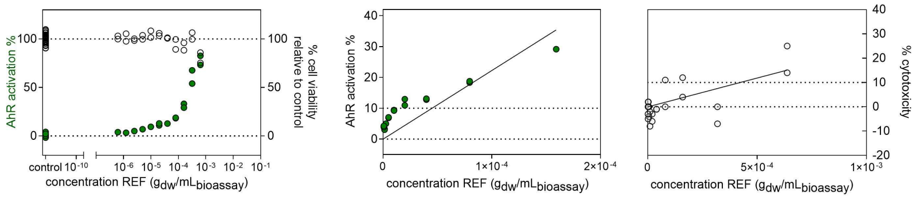

Continue

Riesa pooled fractions No.1-80

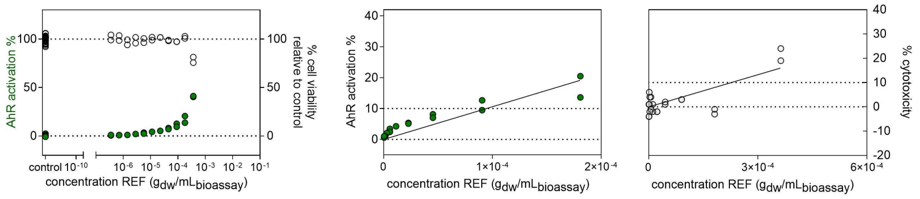

Riesa pooled fractions No.1-52

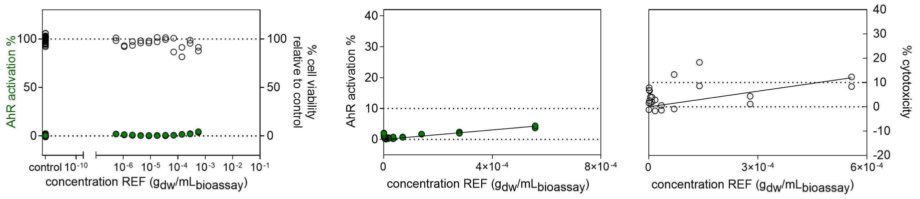

Riesa pooled fractions No.53-80

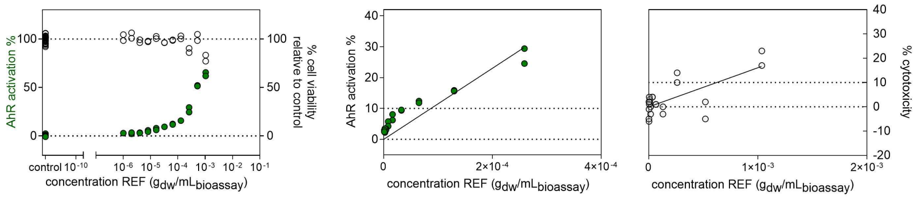

Torgau pooled fractions No.1-80

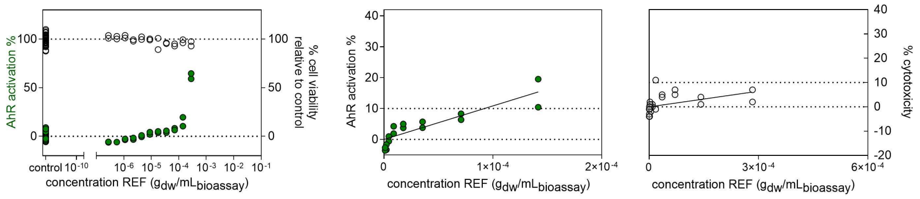

Torgau pooled fractions No.1-52

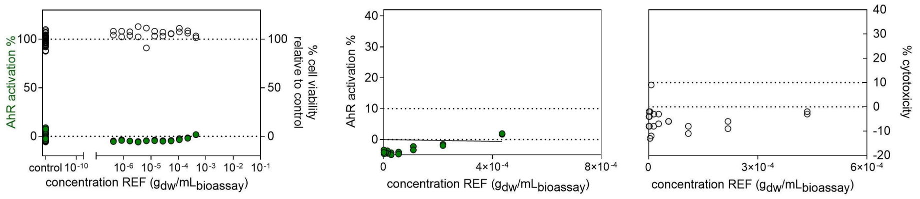

Torgau pooled fractions No.53-80

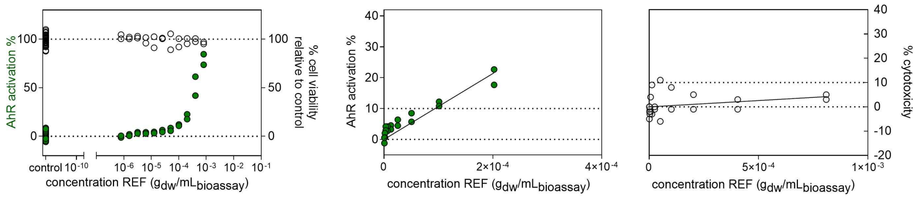

Continue

Wittenberg pooled fractions No.1-80

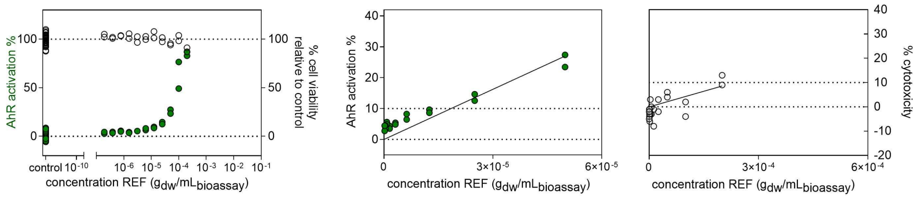

Wittenberg pooled fractions No.1-52

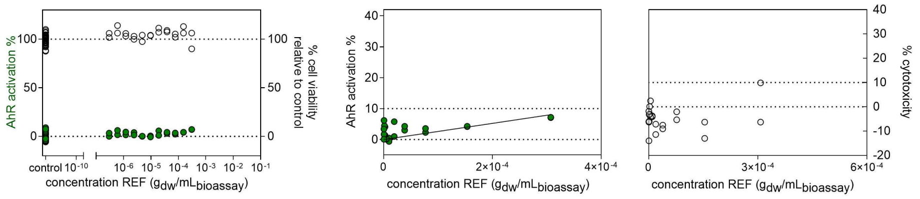

Wittenberg pooled fractions No.53-80

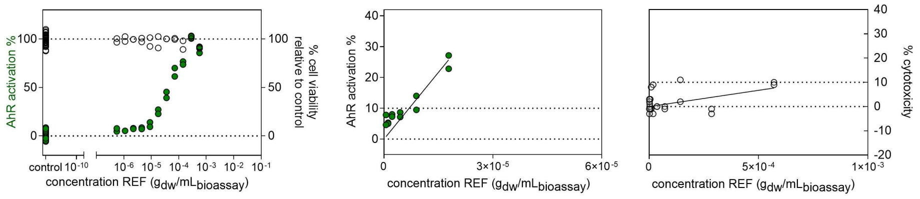

Dessau pooled fractions No.1-80

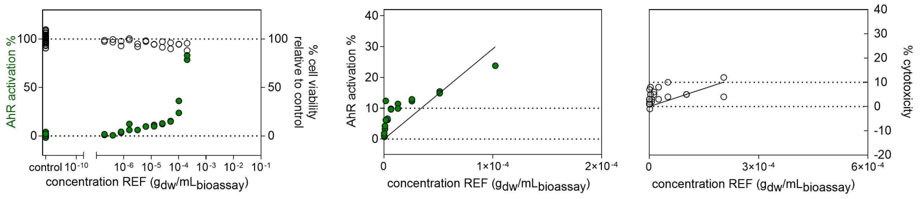

Dessau pooled fractions No.1-52

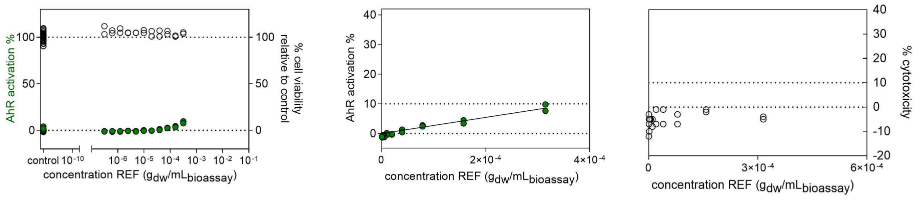

Dessau pooled fractions No.53-80

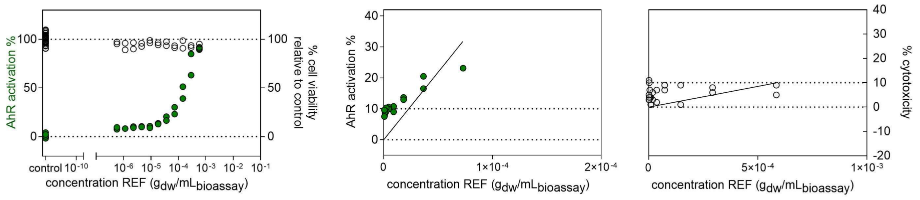

Continue

Magdeburg pooled fractions No.1-80

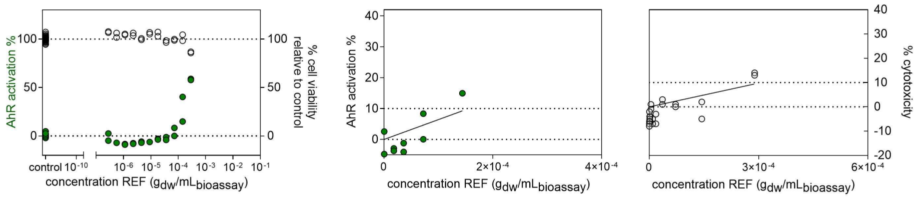

Magdeburg pooled fractions No.1-52

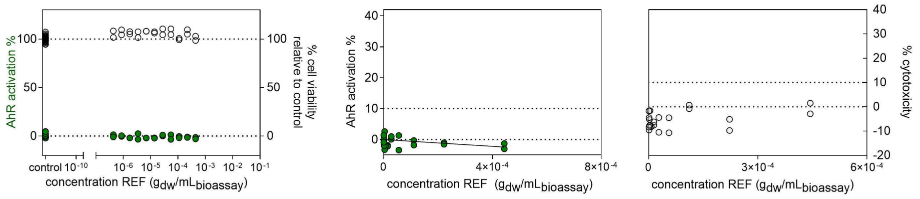

Magdeburg pooled fractions No.53-80

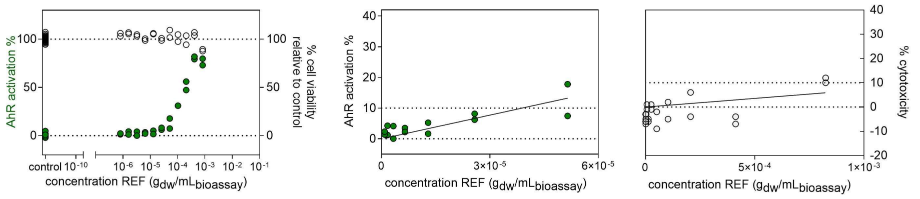

Werben pooled fractions No.1-80

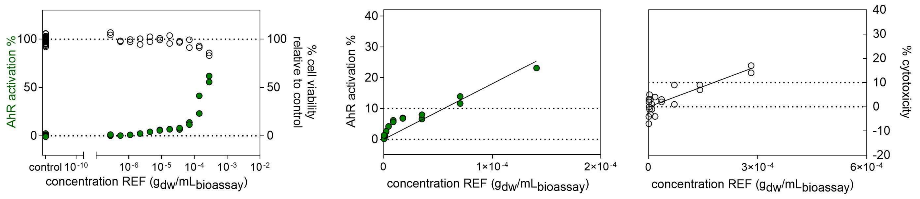

Werben pooled fractions No.1-52

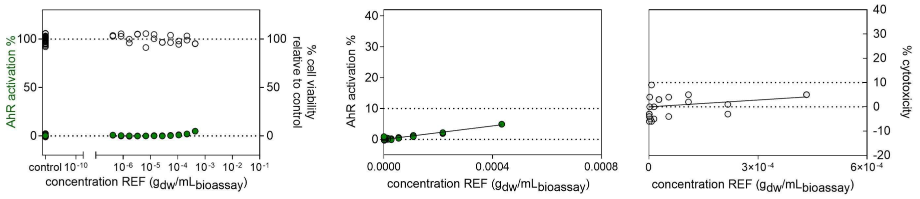

Werben pooled fractions No.53-80

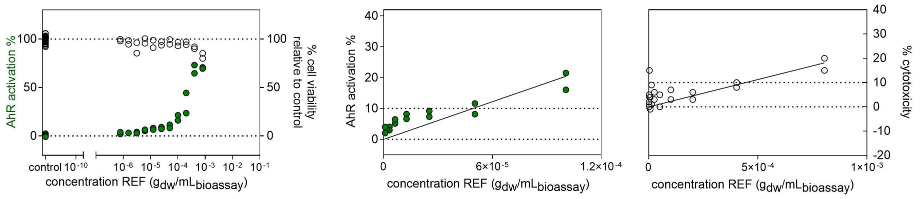

Continue

Domitz pooled fractions No.1-80

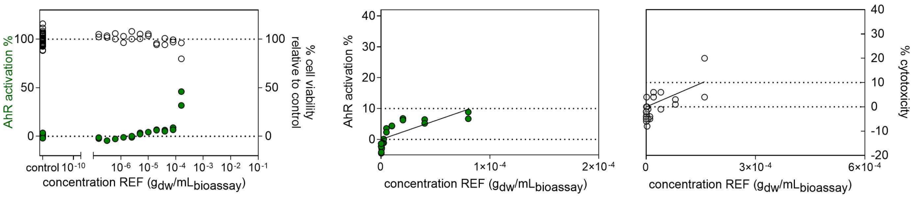

Domitz pooled fractions No.1-52

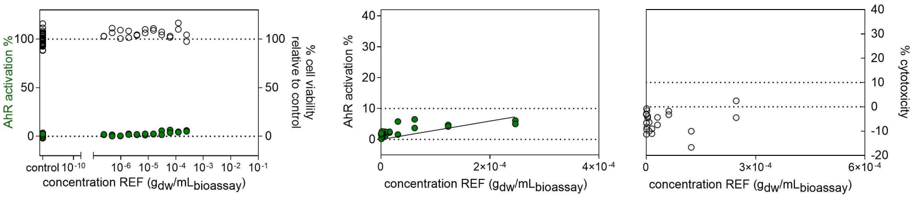

Domitz pooled fractions No.53-80

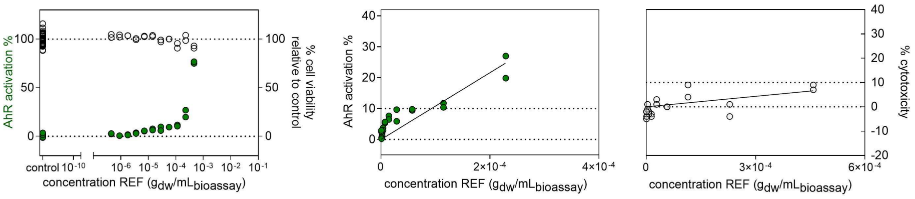

Lauenburg pooled fractions No.1-80

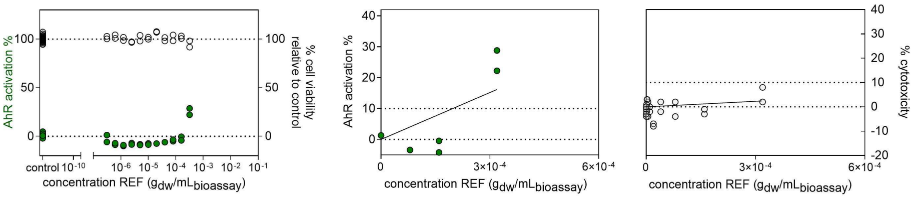

Lauenburg pooled fractions No.1-52

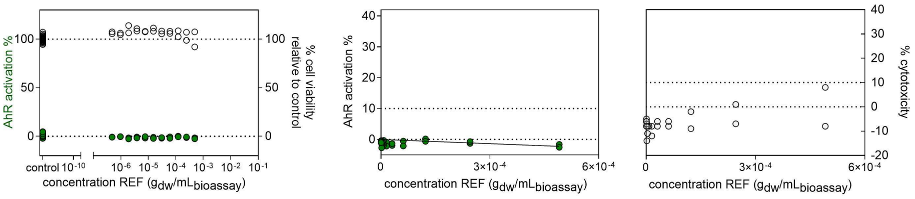

Lauenburg pooled fractions No.53-80

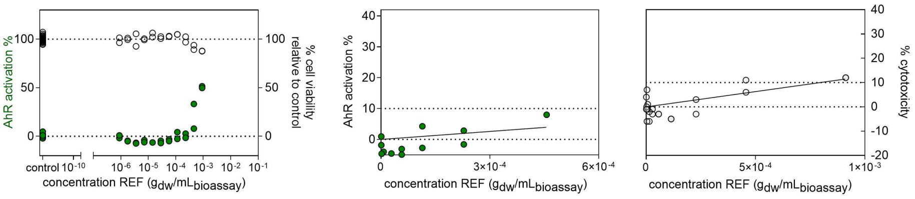

Figure S6. Concentration-response curves of the pooled sediment extract fractions in the AhR CALUX bioassay.

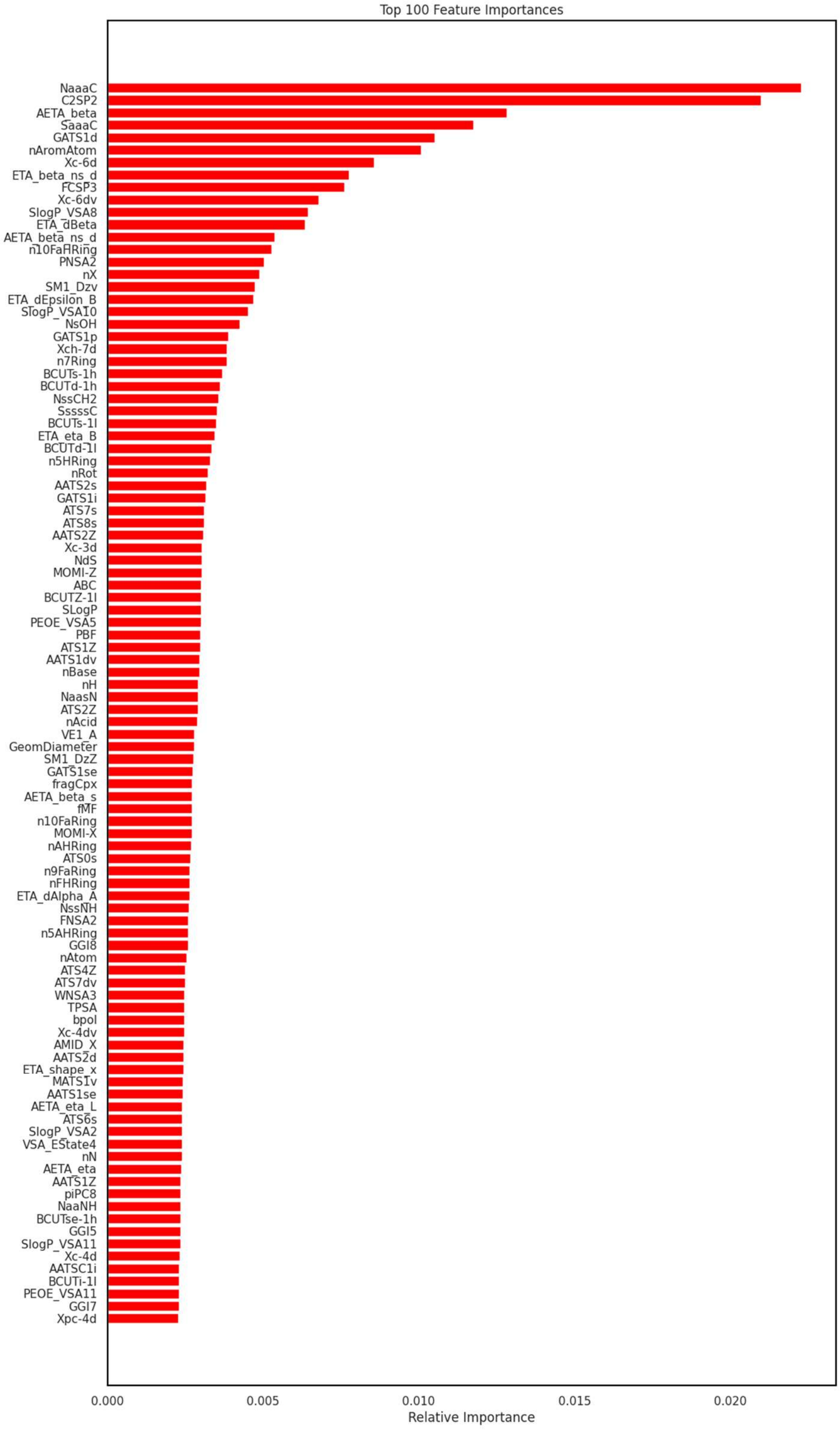

Figure S7. Relative importance of top 100 ranking molecular descriptors in binary classification model. The annotation for each molecular descriptor sees https://mordred-descriptor.github.io/documentation/master/descriptors.html.

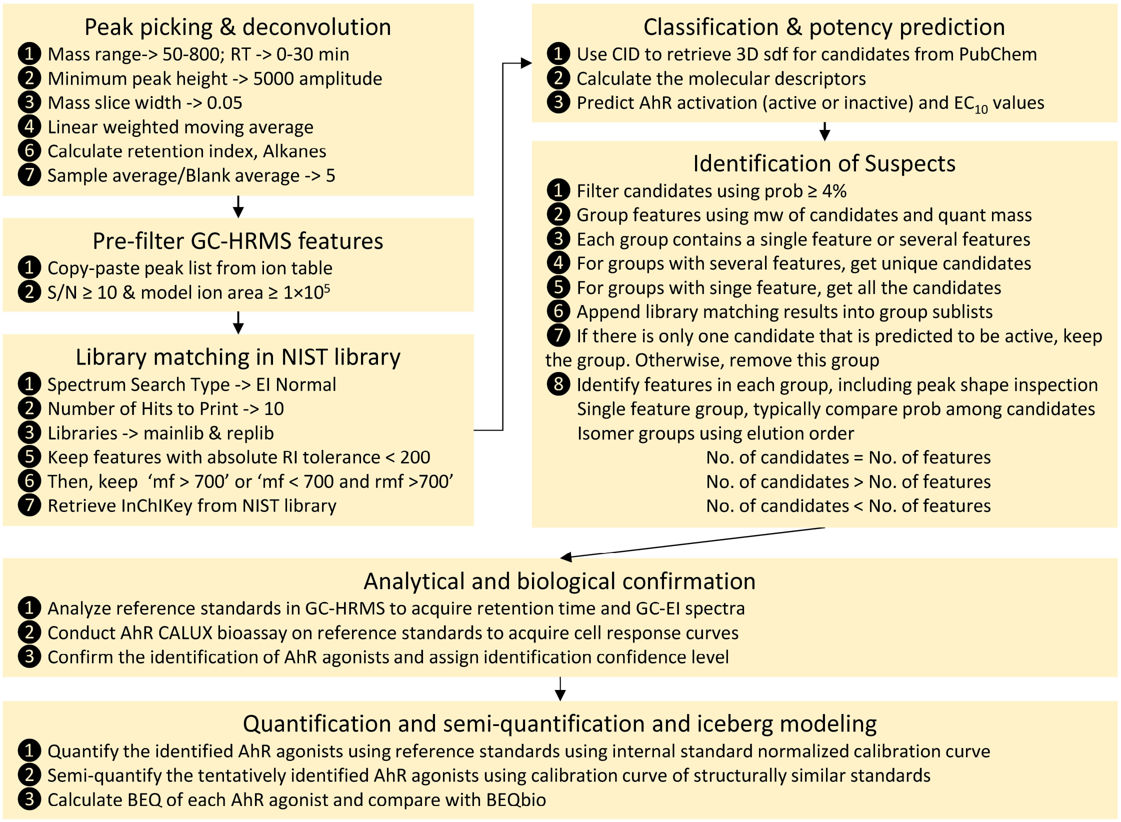

Figure S8. Streamlined suspect screening workflow for GC-HRMS.

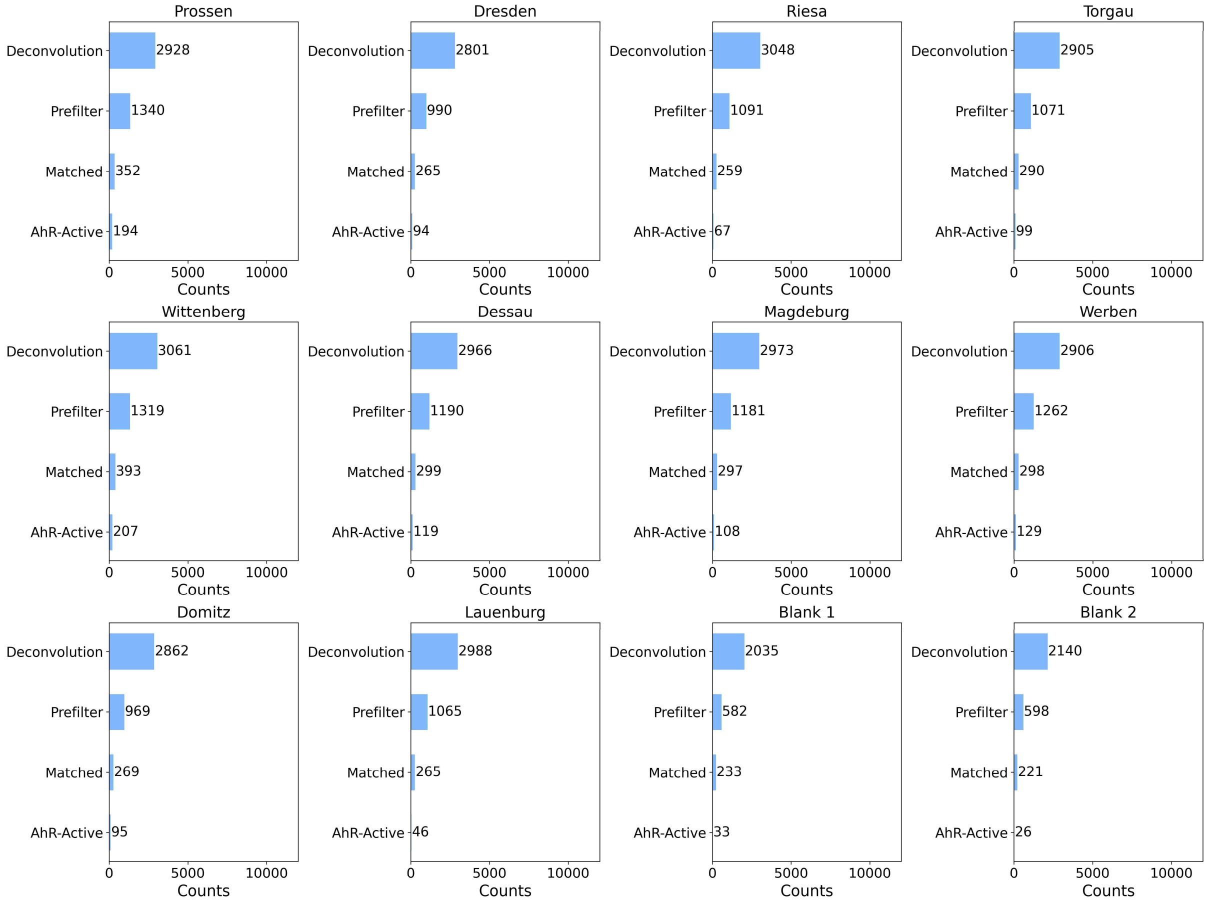

Figure S9. GC-HRMS feature counts in the sediment extracts after each filtering step during suspect screening analysis. Deconvolution: extracted ion chromatograph with GC-EI spectra were constructed from Thermo Fisher Xcalibur (RAW) centroid data of raw extracts using MS-DIAL software; Prefilter: Deconvoluted GC-HRMS features were filtered based on predefined thresholds for signal-to-noise ratio and model ion area; Matched: Definitive match against NIST spectral reference library based on predefined thresholds for match factor, reverse match factor and retention index tolerance; AhR-Active: Predicted AhR-active GC-HRMS features by the established binary classification model.

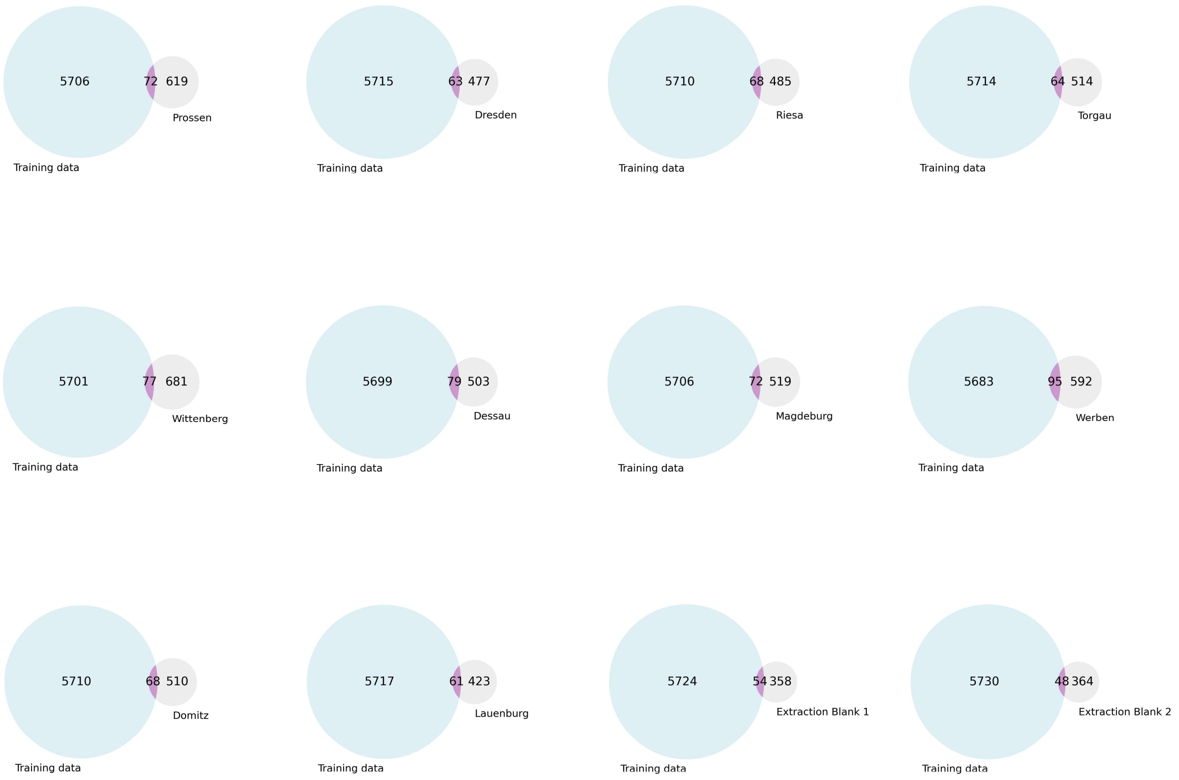

Figure S10. Overlap of chemicals between training data for binary classification and top-ranking candidates after definitive library matching.

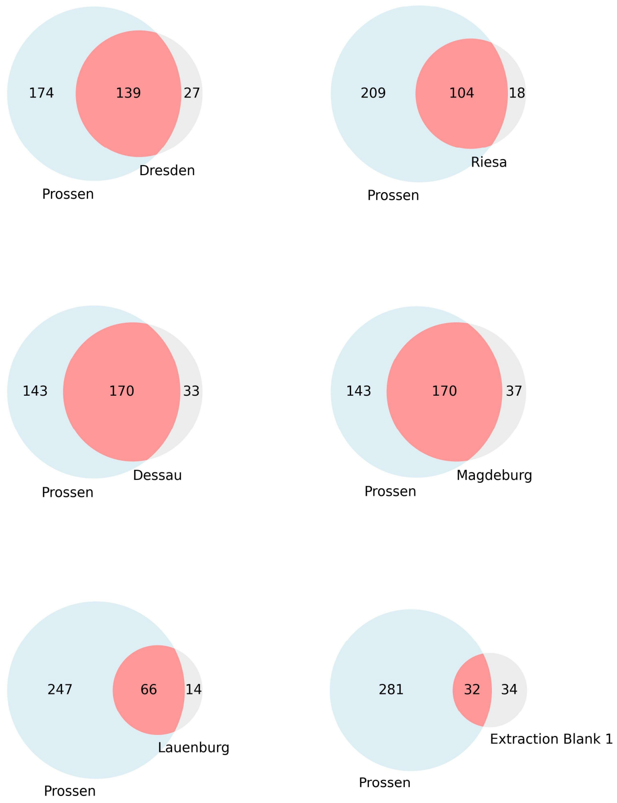

Figure S11. Overlap of top-ranking candidates predicted to be AhR-active between Prossen and other sample extracts.

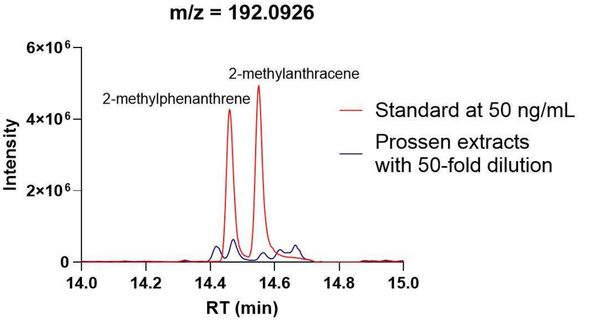
m/z = 192.0926
2-methylphenanthrene
2-methylanthracene
Standard at 50 ng/mL
Prossen extracts
with 50-fold dilution
Intensity
RT (min)

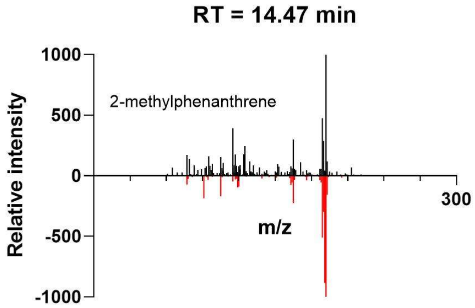
RT = 14.47 min
2-methylphenanthrene
Relative intensity
m/z

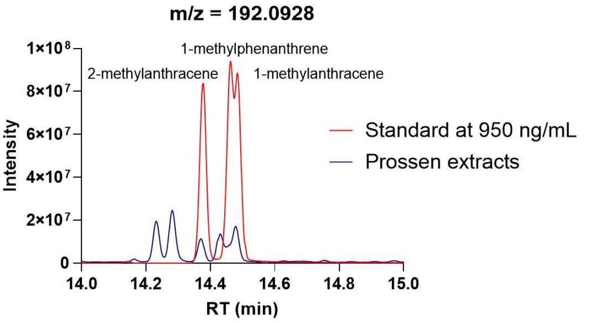
m/z = 192.0928
2-methylanthracene
1-methylphenanthrene
1-methylanthracene
Standard at 950 ng/mL
Prossen extracts
Intensity
RT (min)

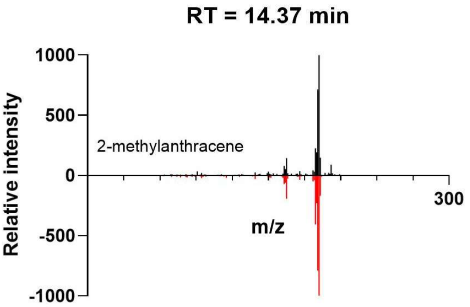
RT = 14.37 min
2-methylanthracene
Relative intensity
m/z

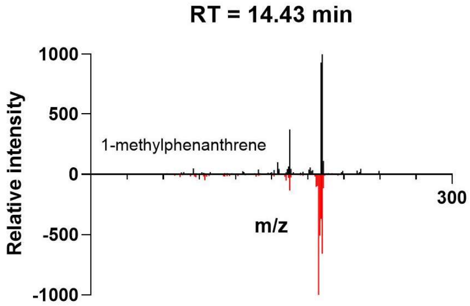
RT = 14.43 min
1-methylphenanthrene
Relative intensity
m/z

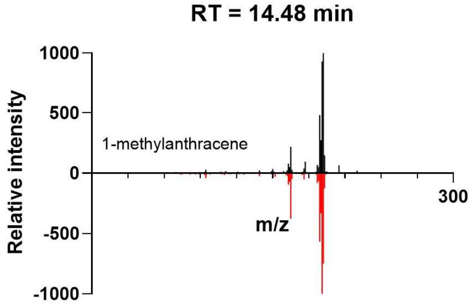
RT = 14.48 min
1-methylanthracene
Relative intensity
m/z

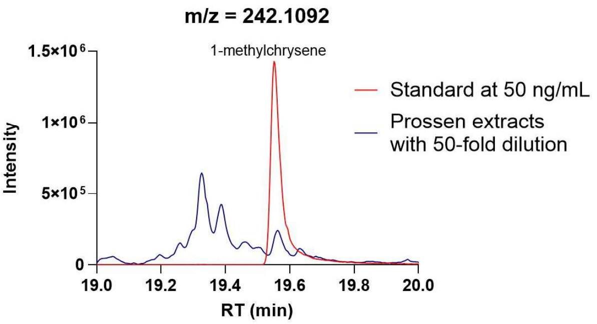
m/z = 242.1092
1-methylchrysene
Standard at 50 ng/mL
Prossen extracts
with 50-fold dilution
Intensity
RT (min)

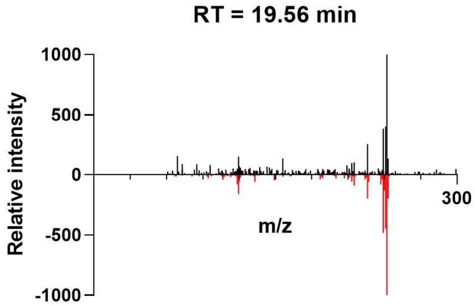
RT = 19.56 min
Relative intensity
m/z

Continue

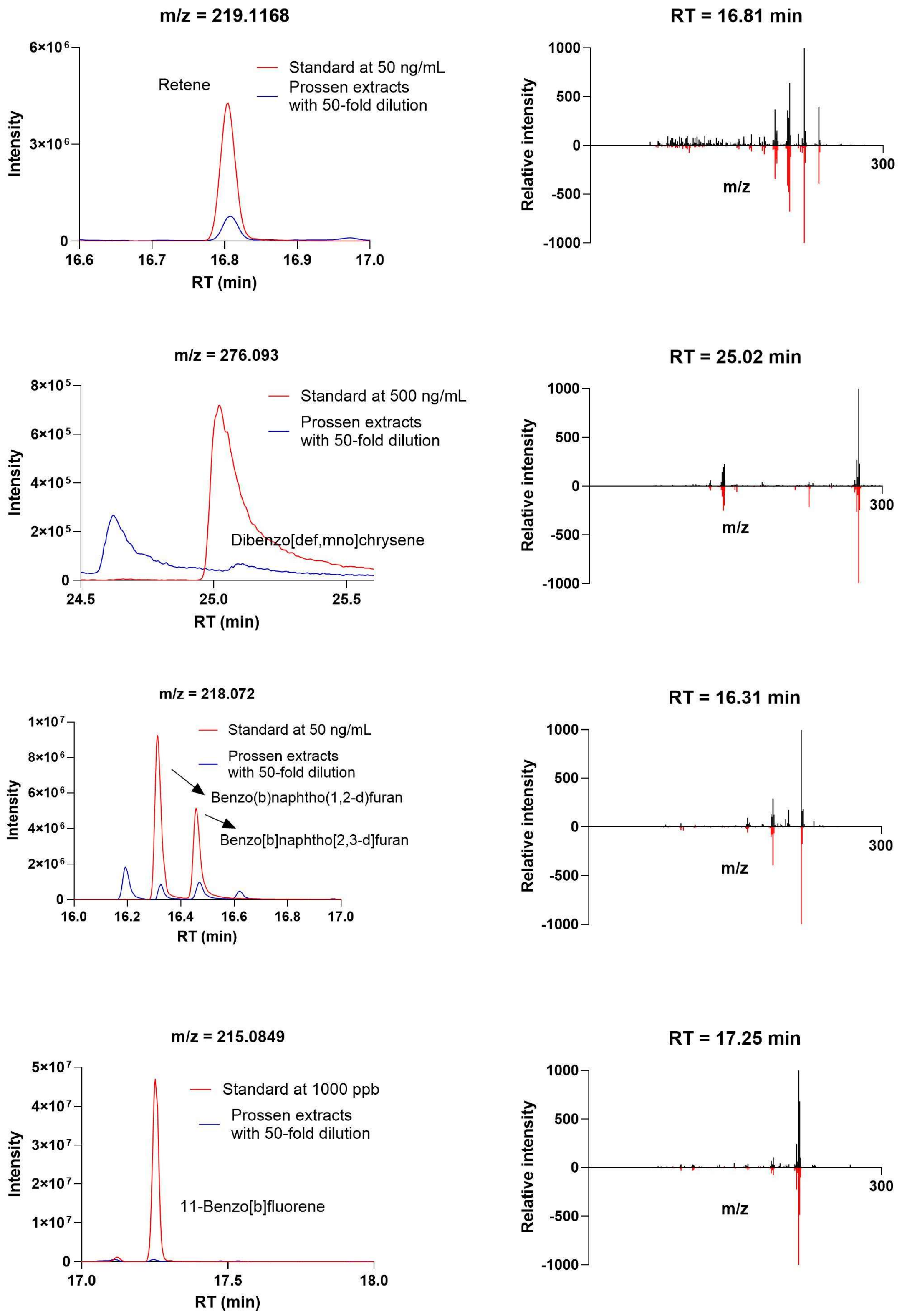
m/z = 219.1168
Retene
Standard at 50 ng/mL
Prossen extracts
with 50-fold dilution
Intensity
RT (min)
RT = 16.81 min
Relative intensity
m/z
m/z = 276.093
Standard at 500 ng/mL
Prossen extracts
with 50-fold dilution
Dibenzo[def,mno]chrysene
Intensity
RT (min)
RT = 25.02 min
Relative intensity
m/z
m/z = 218.072
Standard at 50 ng/mL
Prossen extracts
with 50-fold dilution
Benzo(b)naphtho(1,2-d)furan
Benzo[b]naphtho[2,3-d]furan
Intensity
RT (min)
RT = 16.31 min
Relative intensity
m/z
m/z = 215.0849
Standard at 1000 ppb
Prossen extracts
with 50-fold dilution
11-Benzo[b]fluorene
Intensity
RT (min)
RT = 17.25 min
Relative intensity
m/z

Continue

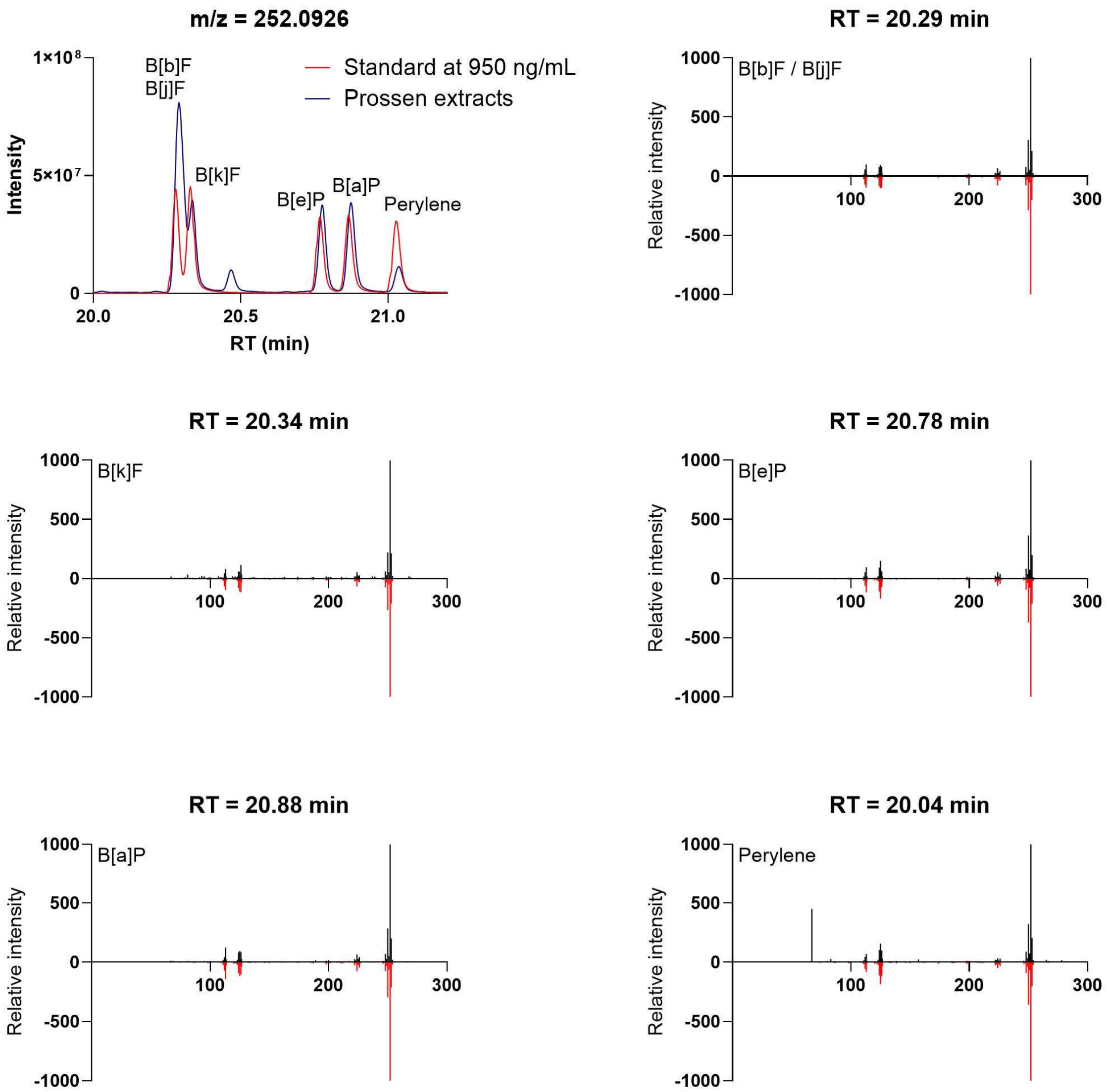
m/z = 252.0926
Standard at 950 ng/mL
Prossen extracts
B[b]F
B[j]F
B[k]F
B[e]P
B[a]P
Perylene
Intensity
RT (min)
RT = 20.29 min
B[b]F / B[j]F
RT = 20.34 min
B[k]F
RT = 20.78 min
B[e]P
RT = 20.88 min
B[a]P
RT = 20.04 min
Perylene
Relative intensity

Continue

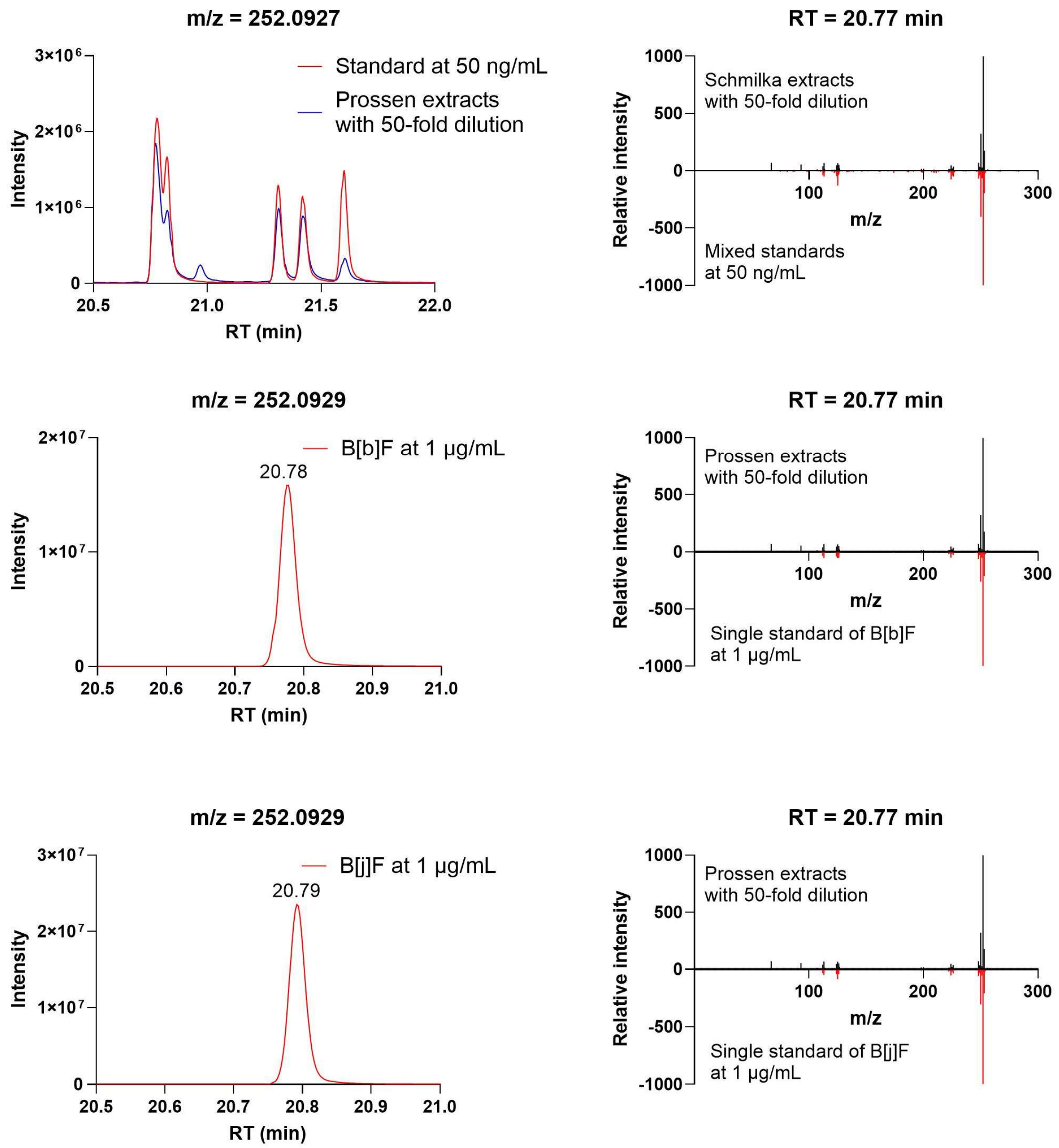
m/z = 252.0927
Standard at 50 ng/mL
Prossen extracts with 50-fold dilution
Intensity
RT (min)
RT = 20.77 min
Schmilka extracts with 50-fold dilution
Mixed standards at 50 ng/mL
Relative intensity
m/z
m/z = 252.0929
B[b]F at 1 μg/mL
20.78
Intensity
RT (min)
RT = 20.77 min
Prossen extracts with 50-fold dilution
Single standard of B[b]F at 1 μg/mL
Relative intensity
m/z
m/z = 252.0929
B[j]F at 1 μg/mL
20.79
Intensity
RT (min)
RT = 20.77 min
Prossen extracts with 50-fold dilution
Single standard of B[j]F at 1 μg/mL
Relative intensity
m/z

Continue

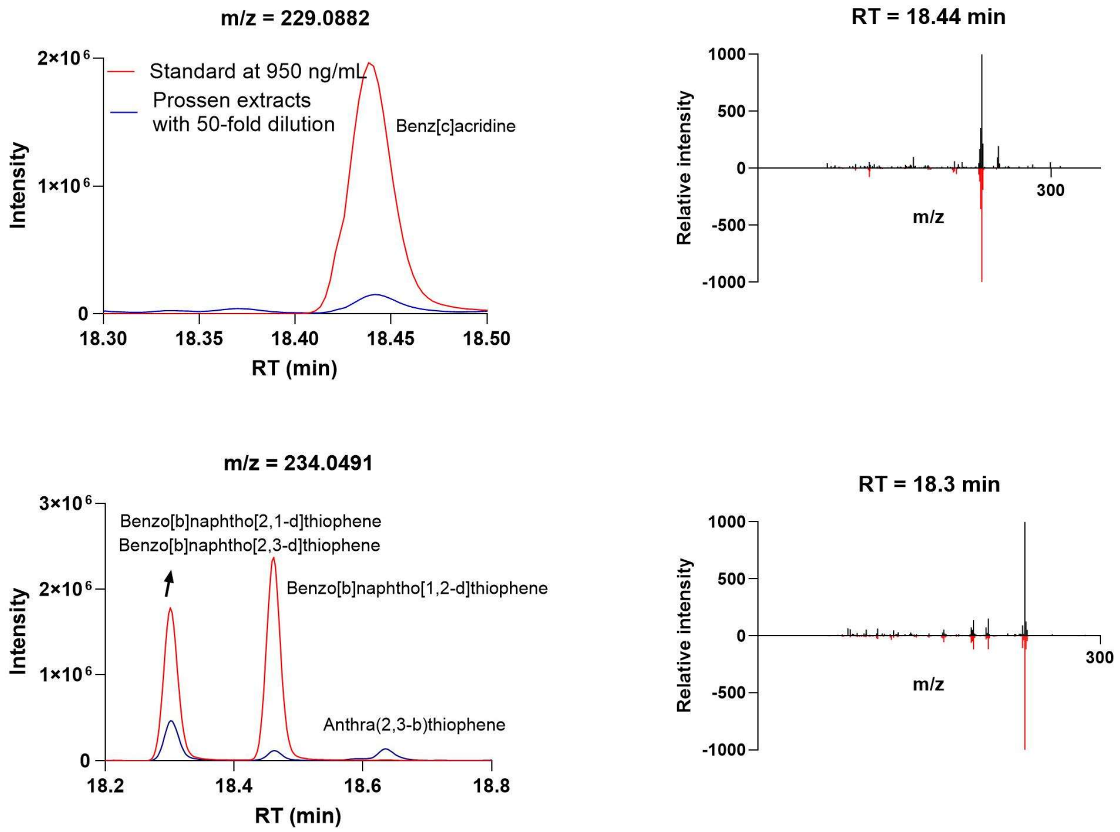

Figure S12. Overlay of extracted ion chromatographs for identified AhR agonists in sample extracts and reference standards, along with their head-to-tail GC-EI spectra. Prossen sediment extracts and Prossen sediment extracts with 50-fold dilution were analyzed using GC-HRMS in two separate batches. Consequently, the retention time of the same chemical compound may vary slightly due to the cut of GC column. The retention time from the first batch was reported in tables.

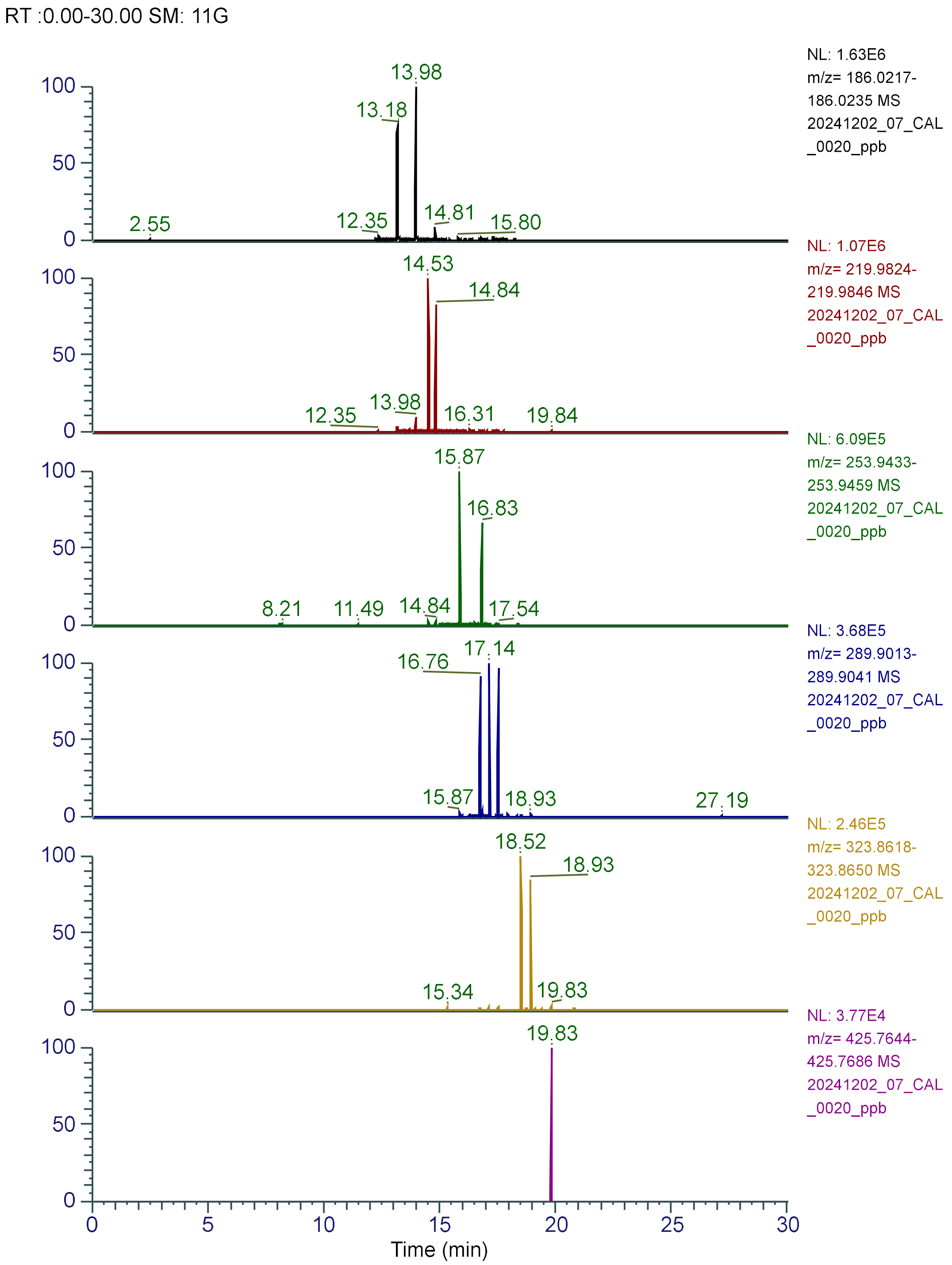

Total ion chromatograms for PCB standards at 20 ng/mL acetonitrile. From the top to last panel and from left to right is PCB 18, PCB 28 (panel 1); PCB 52, PCB 44 (panel 2); PCB 101, PCB 118 (panel 3); PCB 149, PCB 153, and PCB 138 (panel 4); PCB 180, PCB 170 (panel 5); PCB 194 (panel 6).

Continue

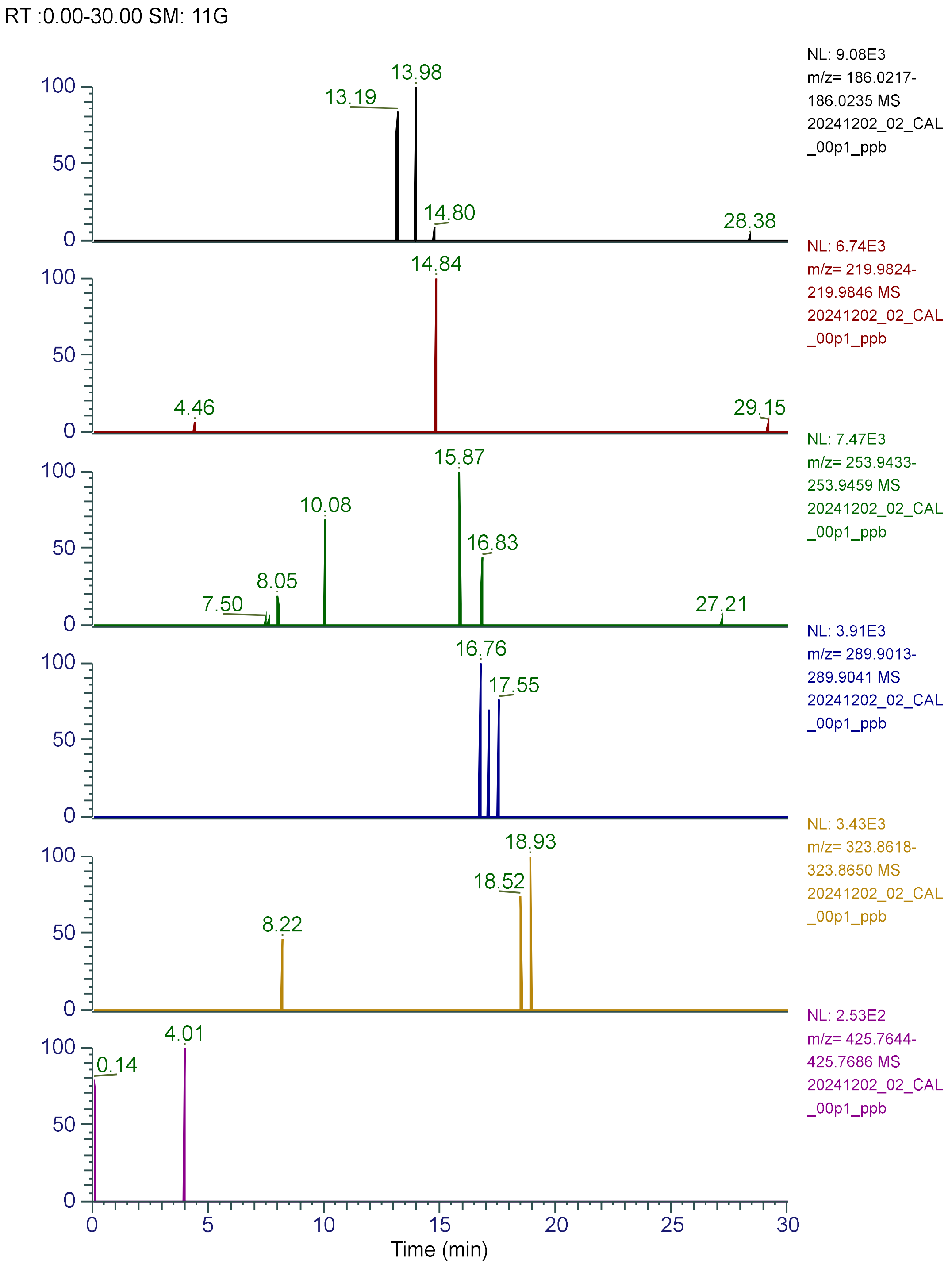

Total ion chromatograms for PCB standards at 0.1 ng/ mL acetonitrile.

Continue

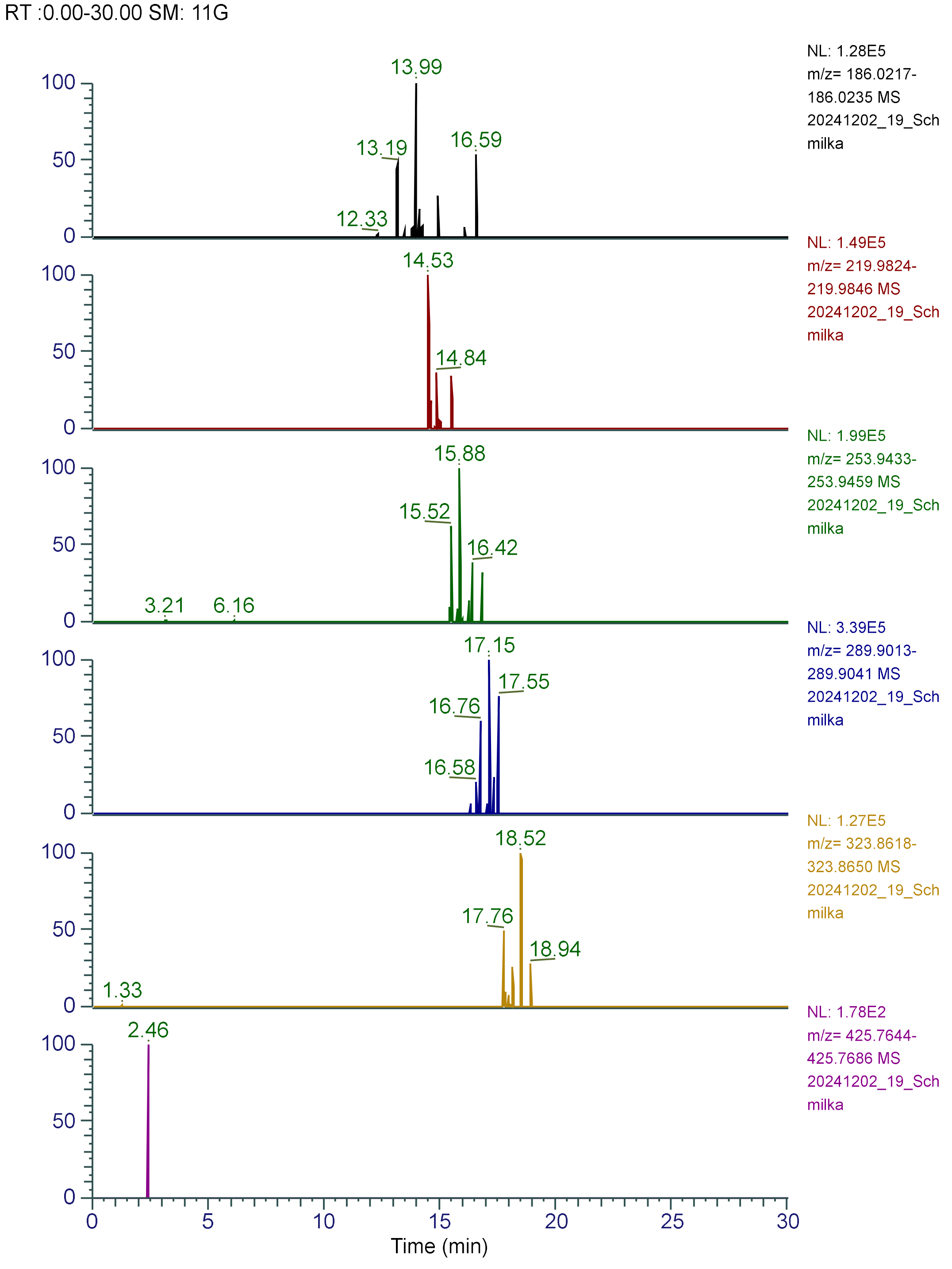

Total ion chromatograms showing molecular ions within 5 ppm of the PCB standards in Prossen sediment extracts.

Continue

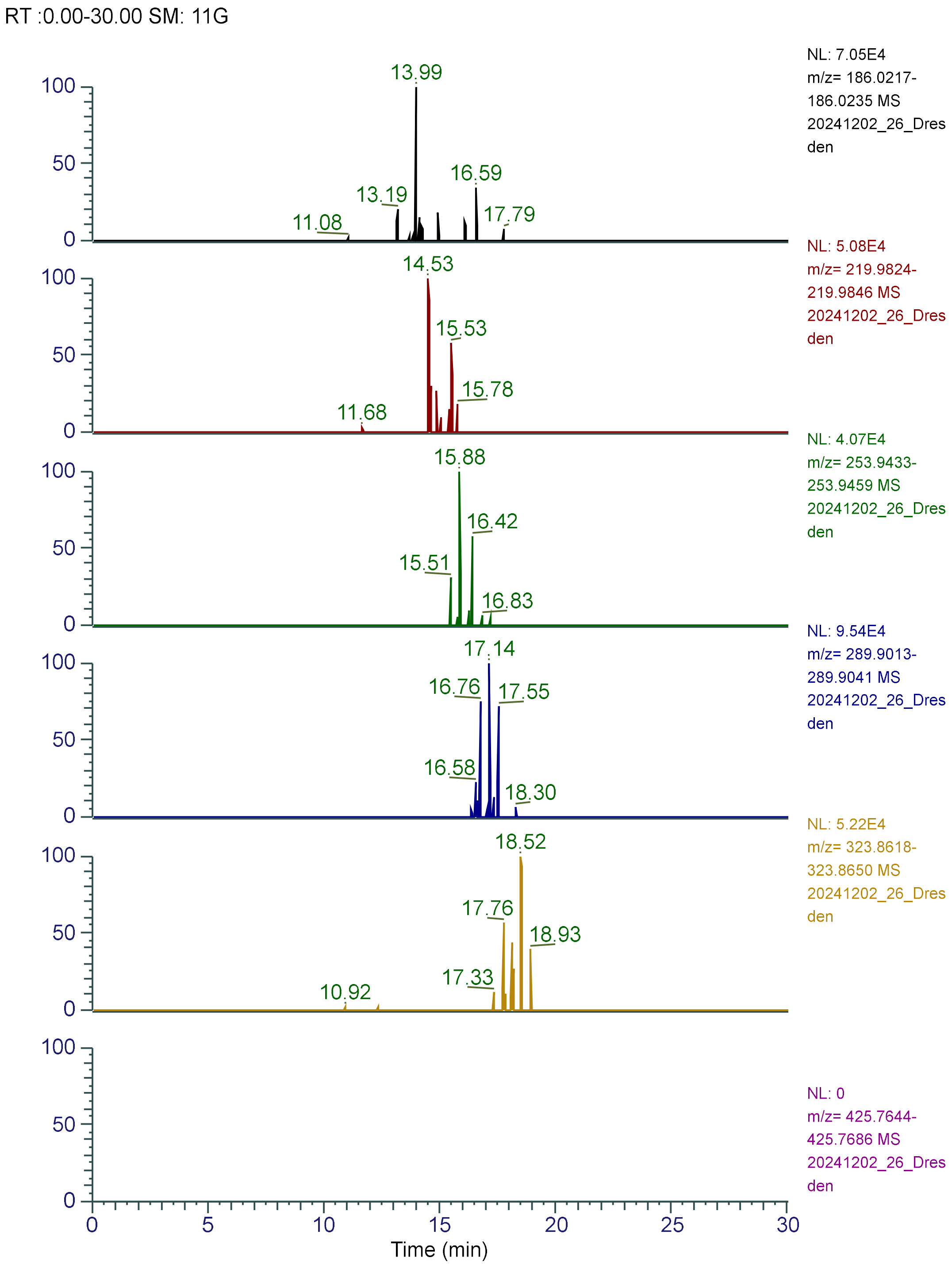

Total ion chromatograms showing molecular ions within 5 ppm of the PCB standards in Dresden sediment extracts.

Continue

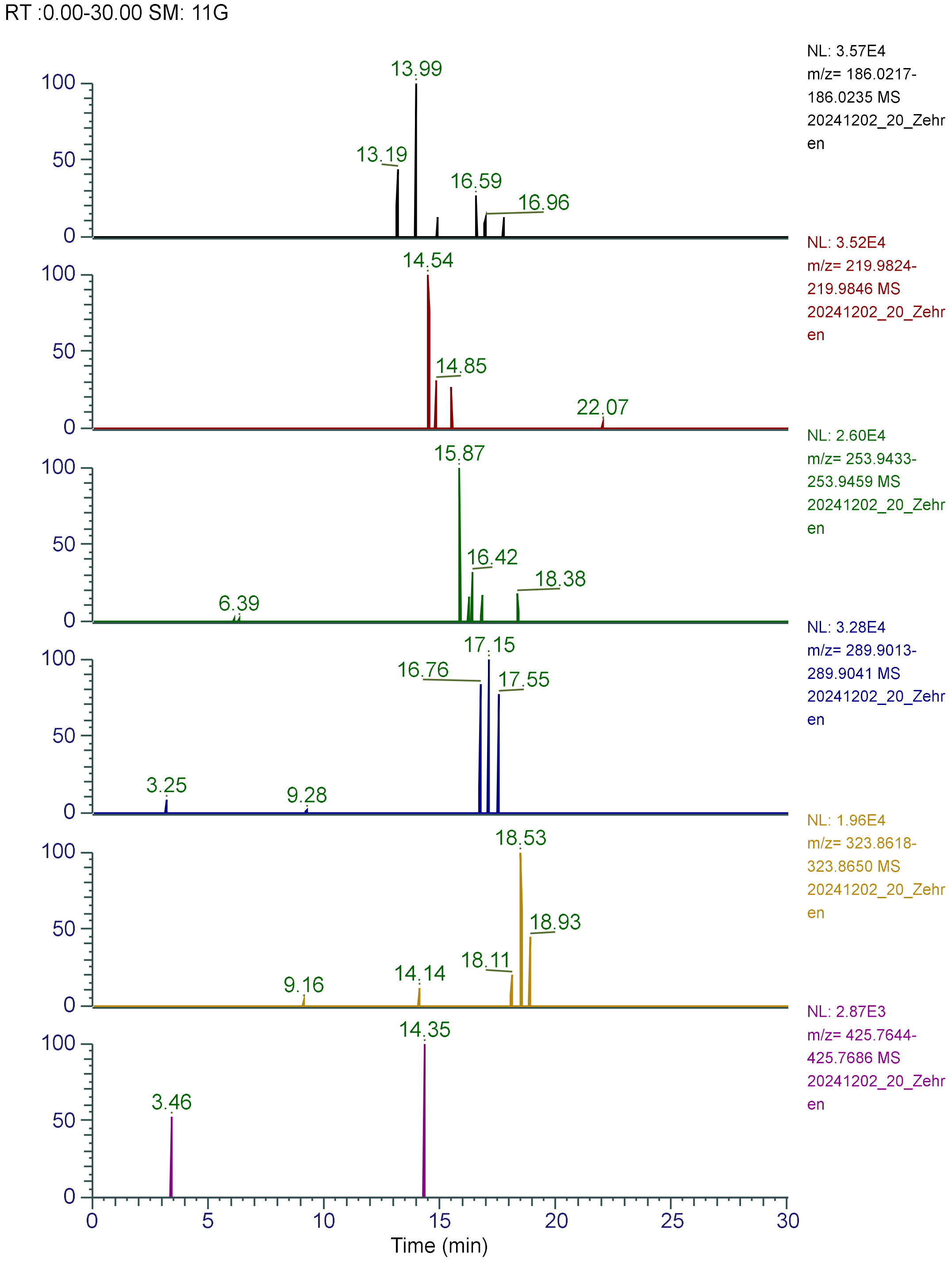

Total ion chromatograms showing molecular ions within 5 ppm of the PCB standards in Riesa sediment extracts.

Continue

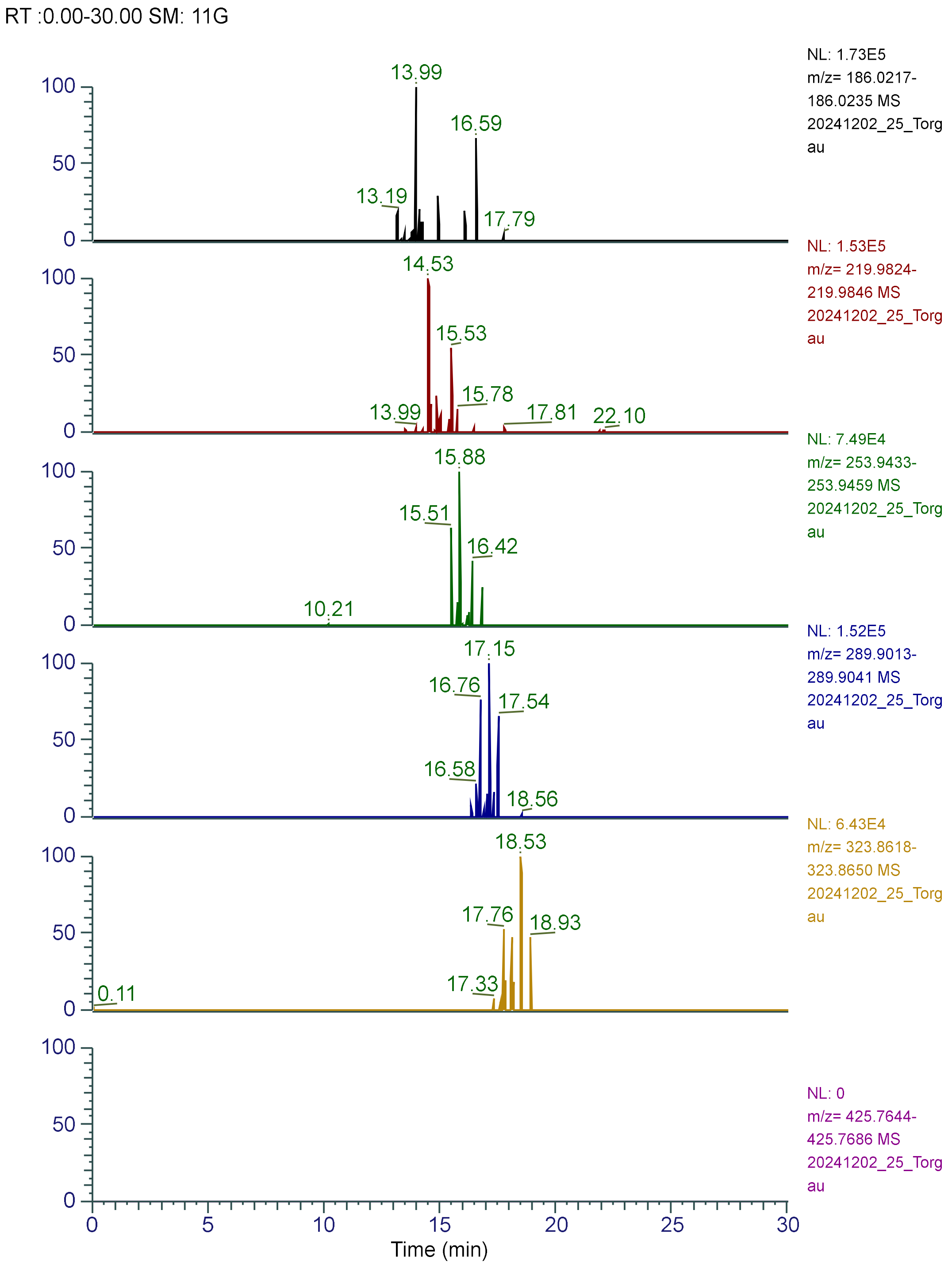

Total ion chromatograms showing molecular ions within 5 ppm of the PCB standards in Torgau sediment extracts.

Continue

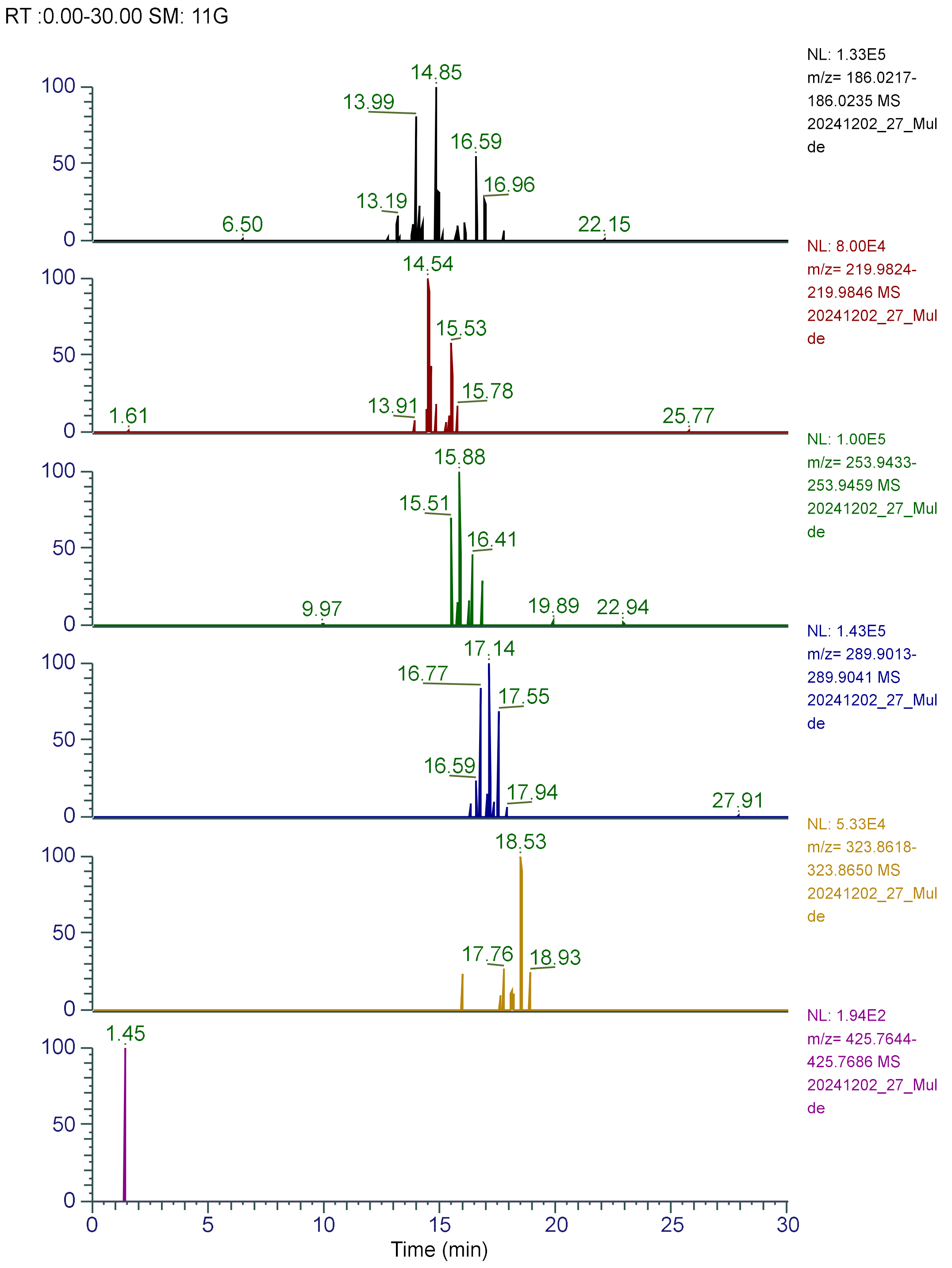

Total ion chromatograms showing molecular ions within 5 ppm of the PCB standards in Dessau sediment extracts.

Continue

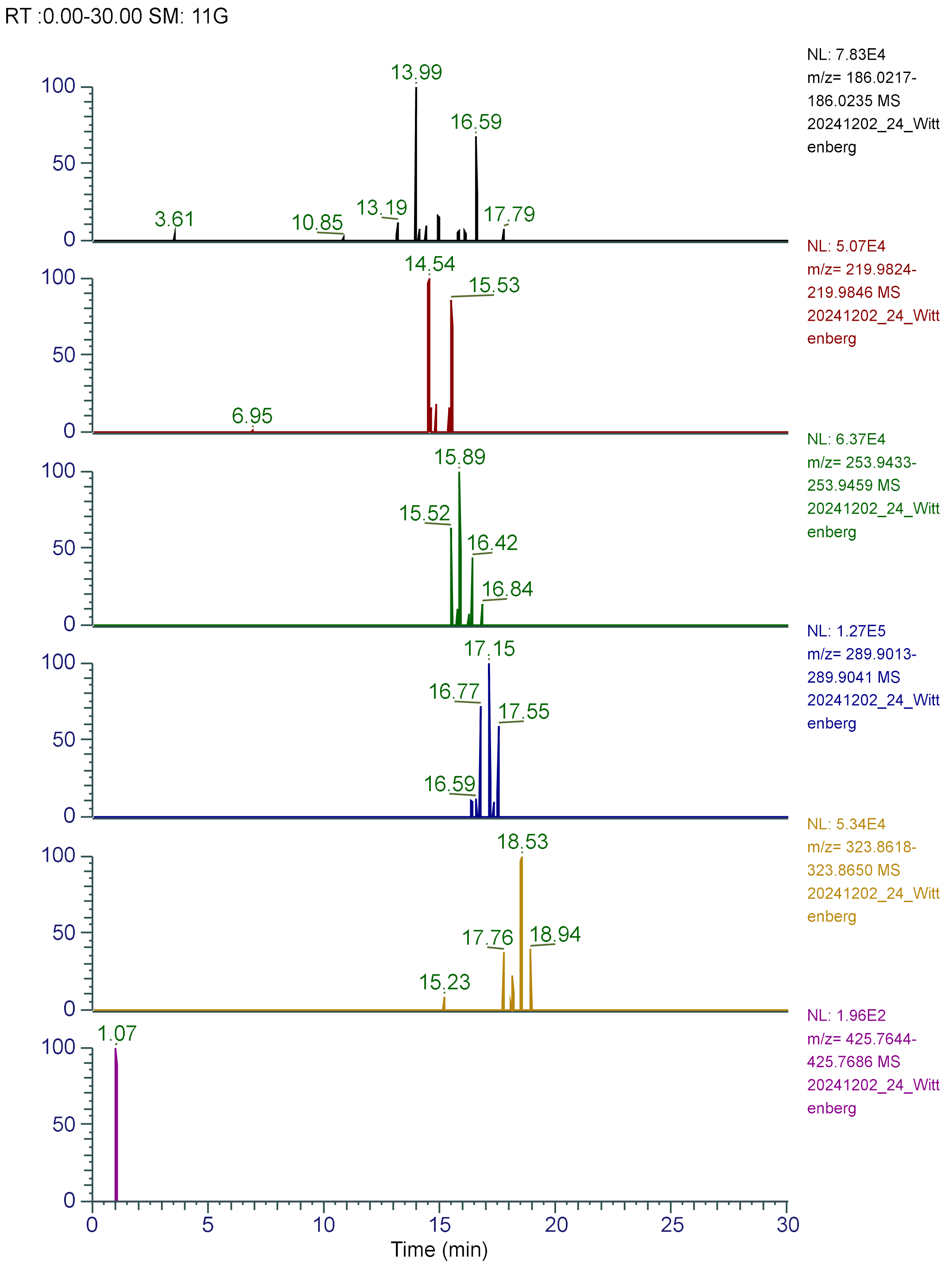

Total ion chromatograms showing molecular ions within 5 ppm of the PCB standards in Wittenberg sediment extracts.

Continue

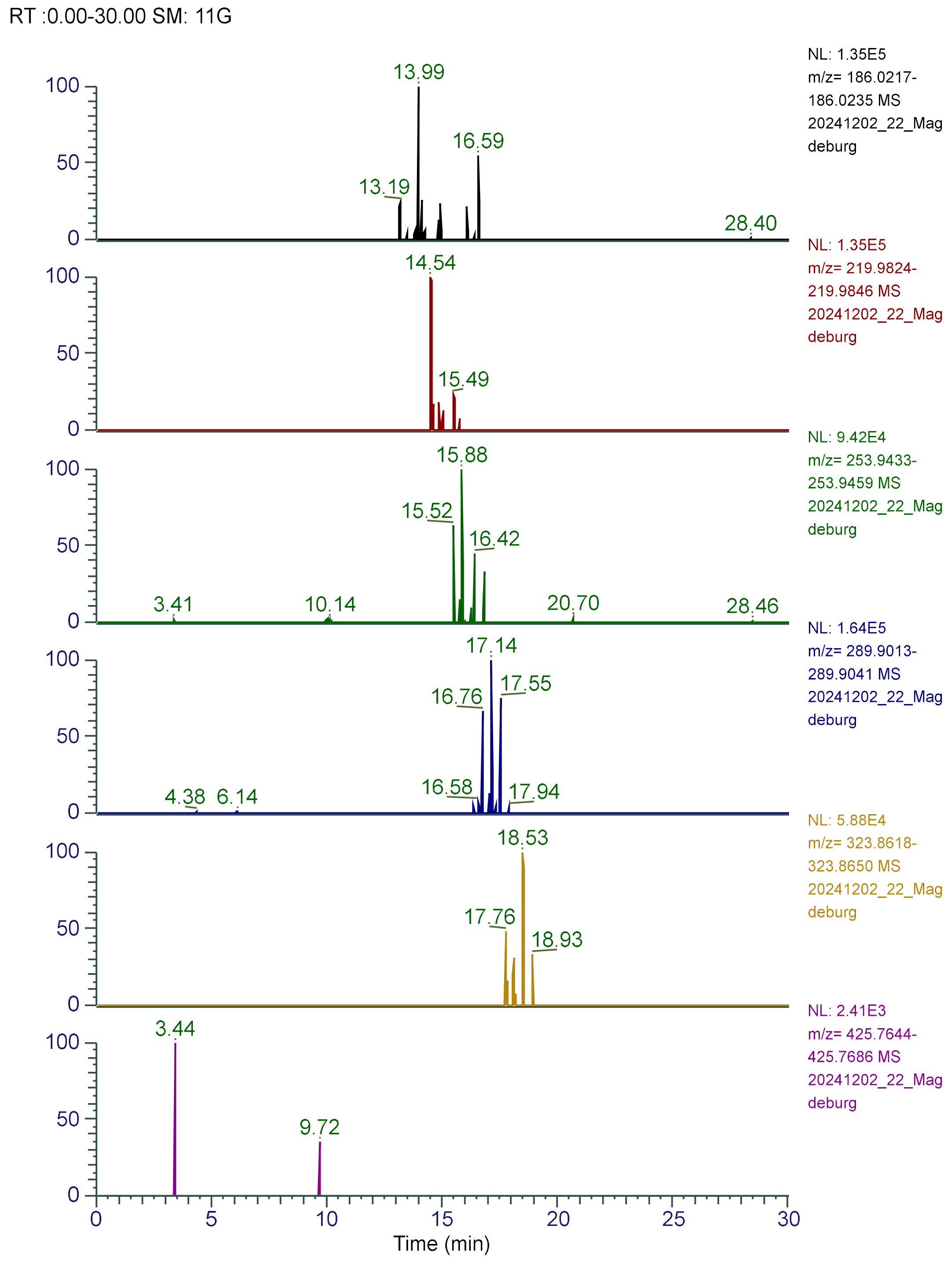

Total ion chromatograms showing molecular ions within 5 ppm of the PCB standards in Magdeburg sediment extracts.

Continue

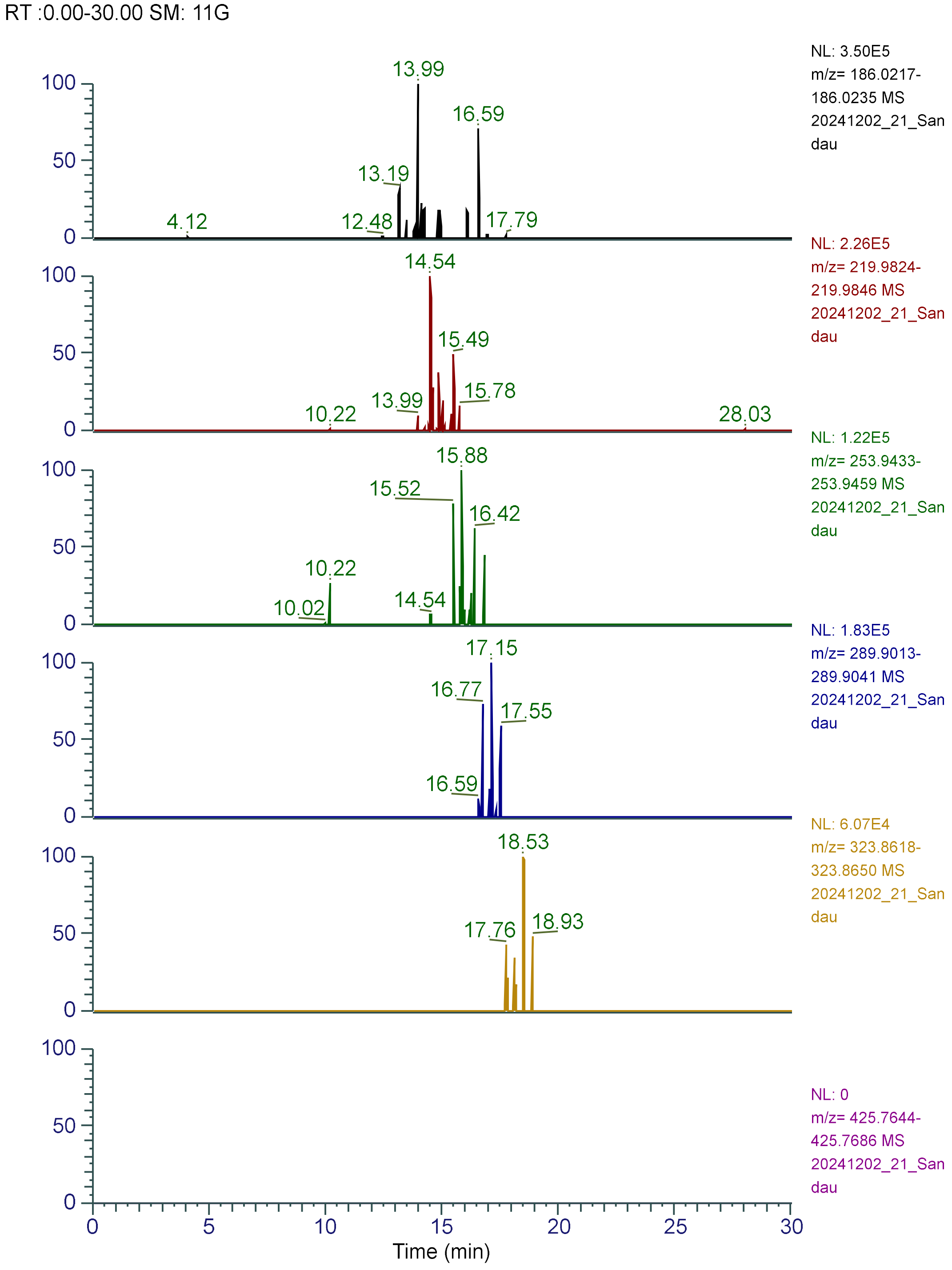

Total ion chromatograms showing molecular ions within 5 ppm of the PCB standards in Werben sediment extracts.

Continue

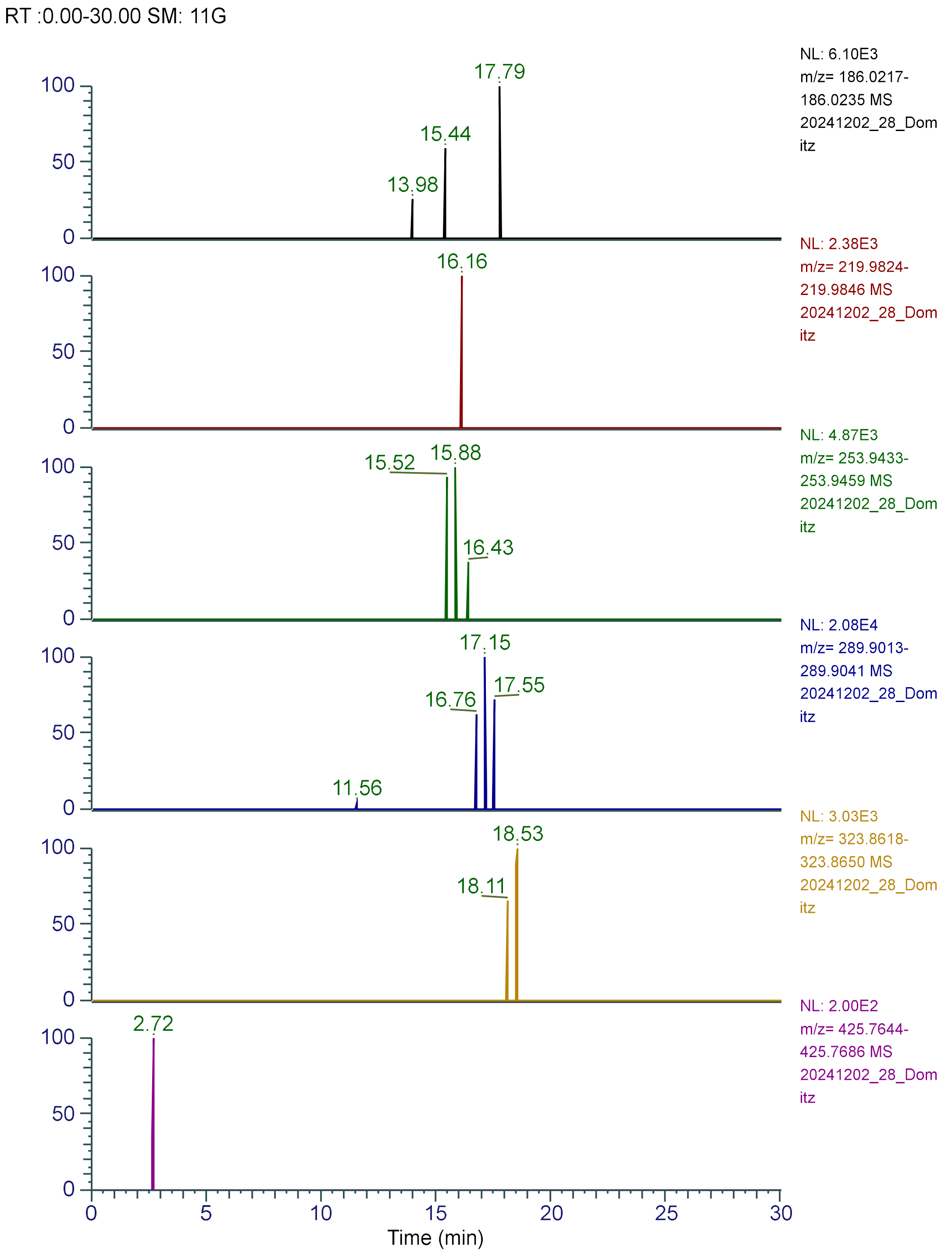

Total ion chromatograms showing molecular ions within 5 ppm of the PCB standards in Domitz sediment extracts.

Continue

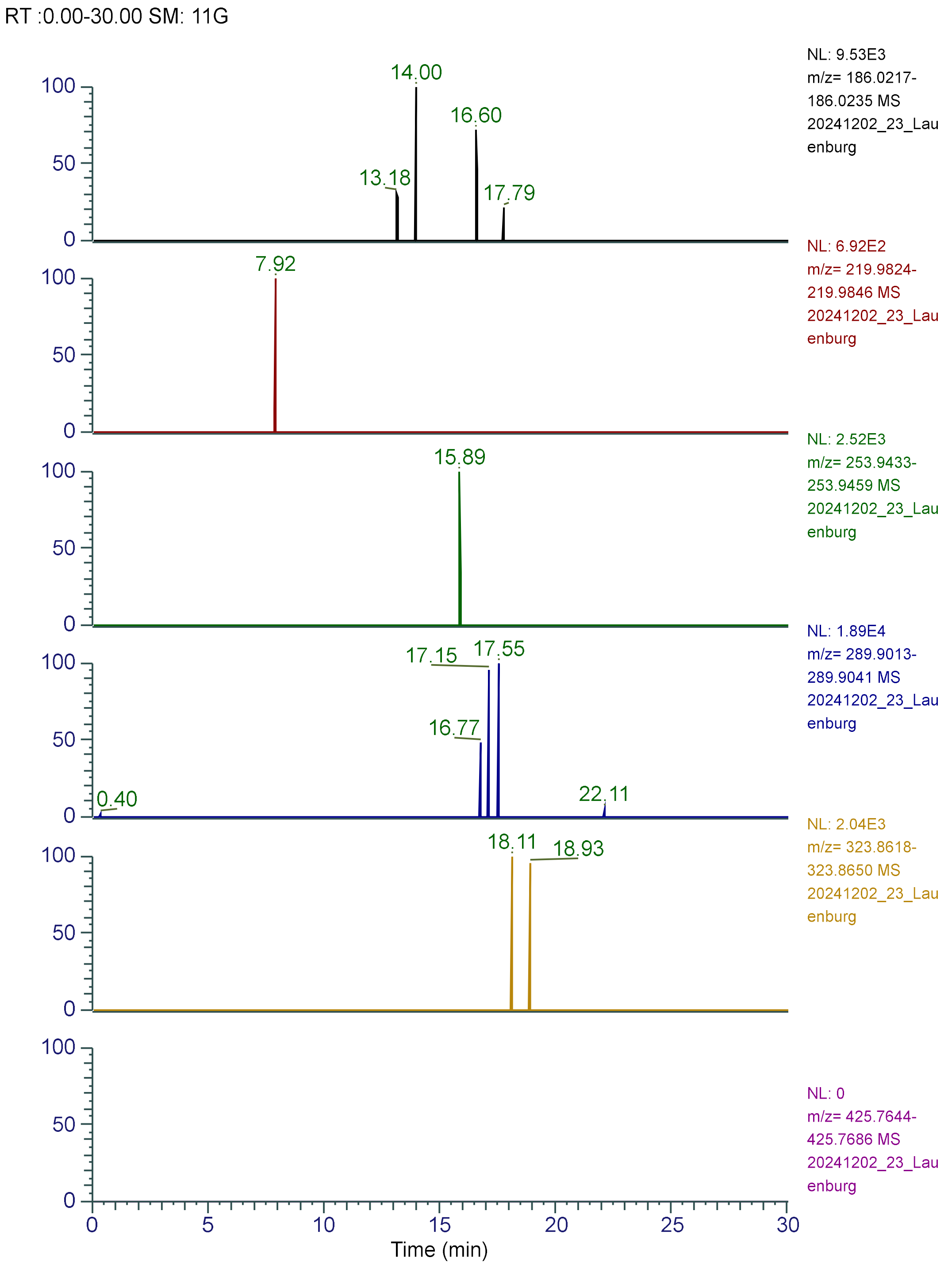

Total ion chromatograms showing molecular ions within 5 ppm of the PCB standards in Lauenburg sediment extracts.

Continue

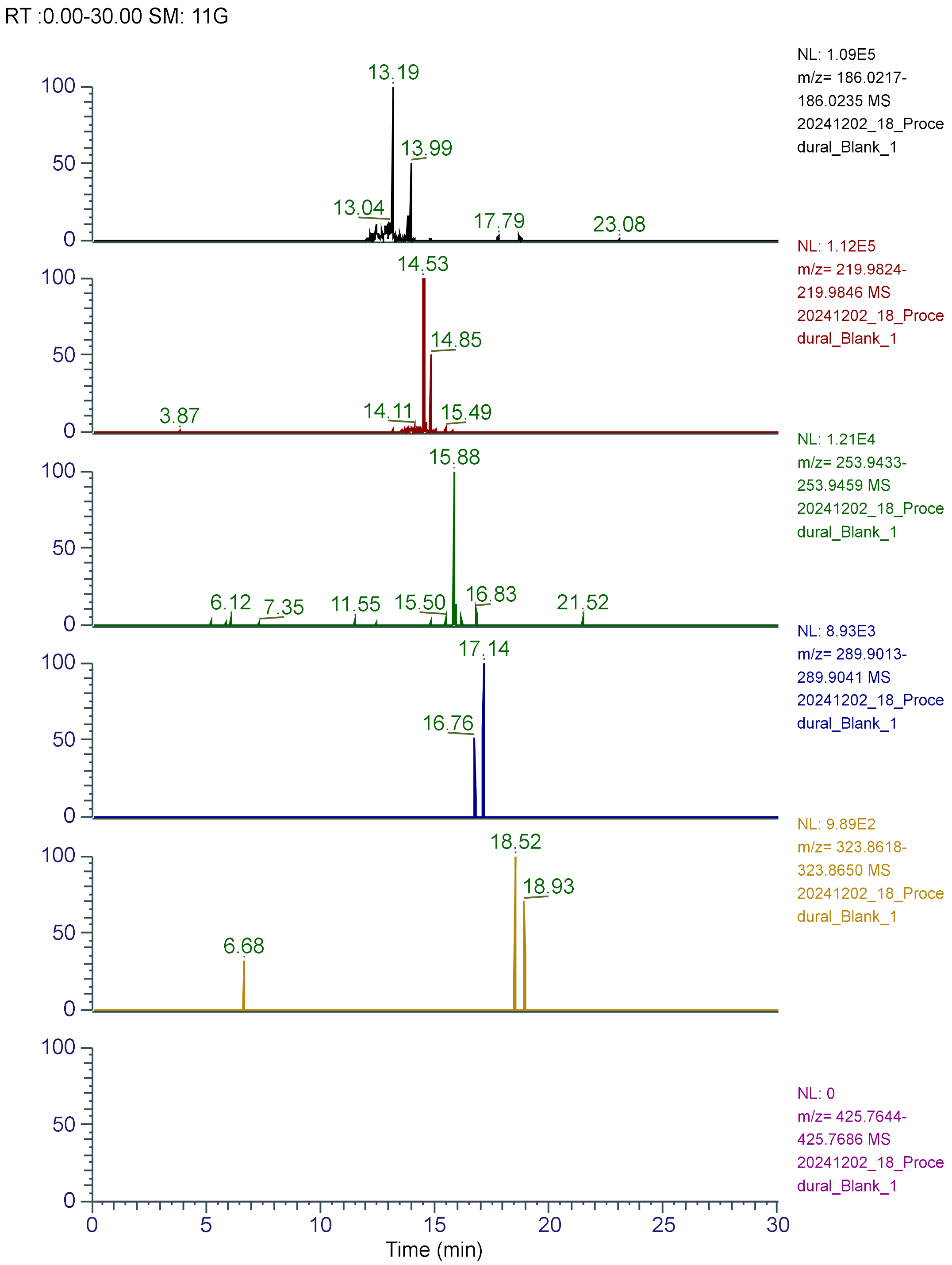

Total ion chromatograms showing molecular ions within 5 ppm of the PCB standards in extraction blank 1 extracts.

Continue

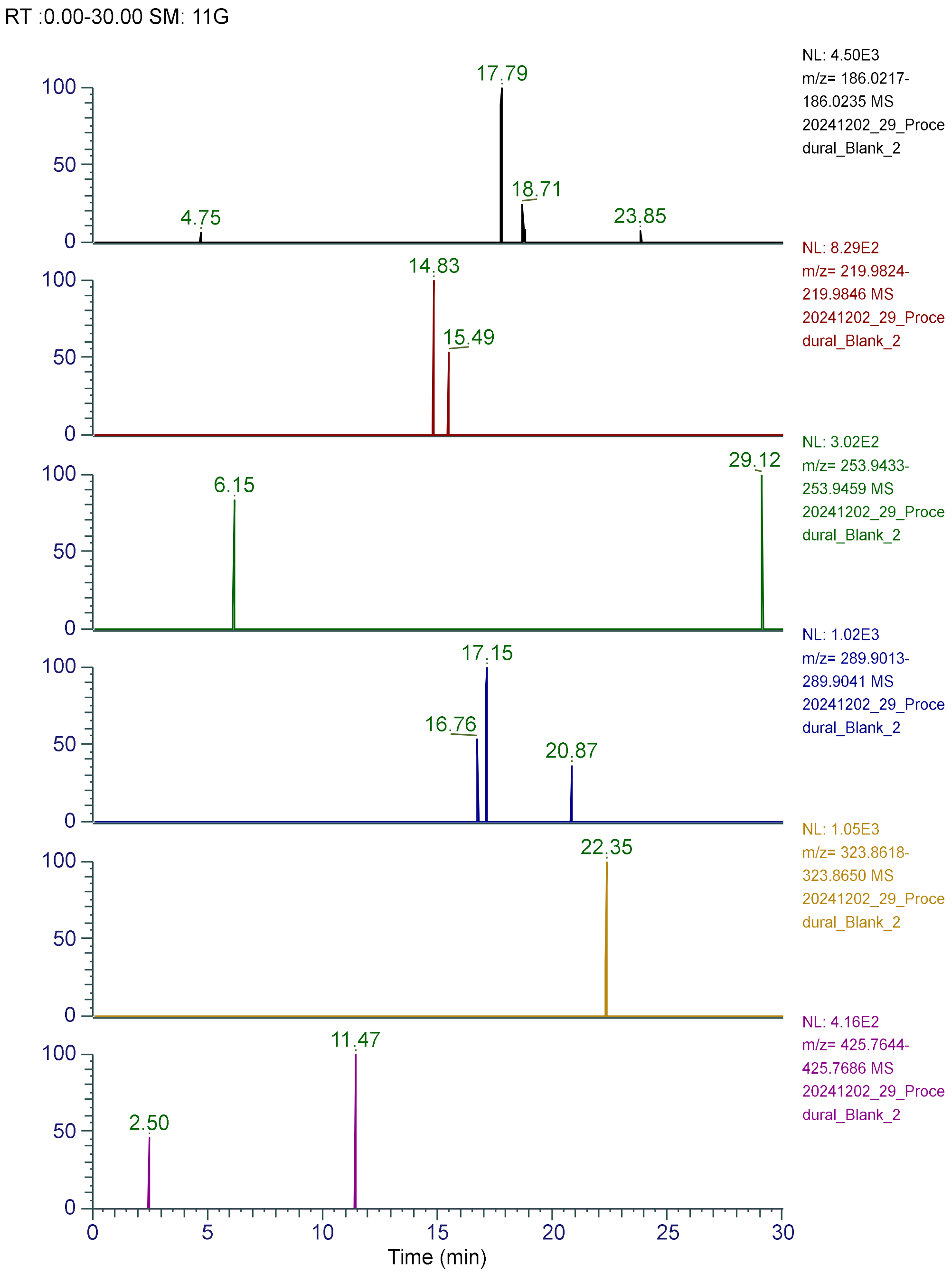

Total ion chromatograms showing molecular ions within 5 ppm of the PCB standards in extraction blank 2 extracts.

Figure S13. Total ion chromatograms of PCBs in reference standard and sediment extracts.

Phenanthrene

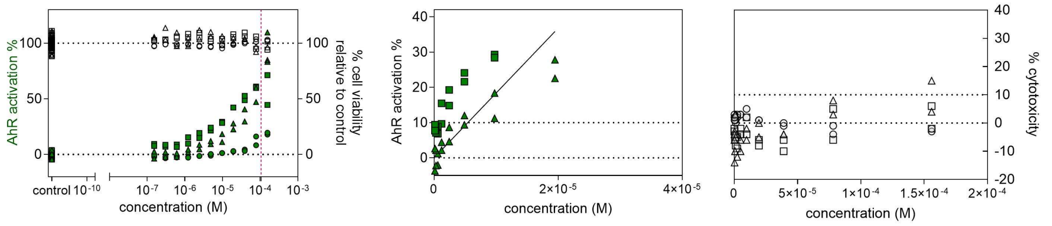

Anthracene

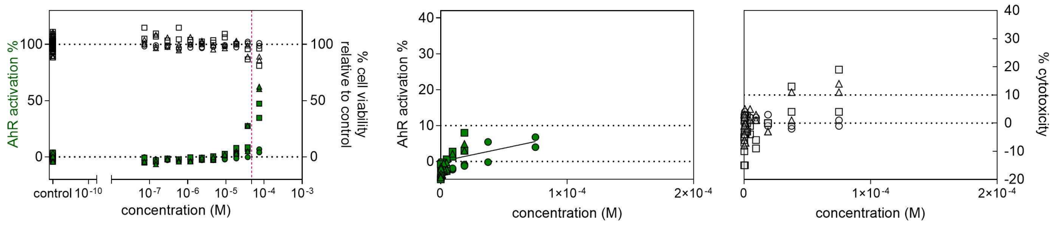

2-Methylphenanthrene

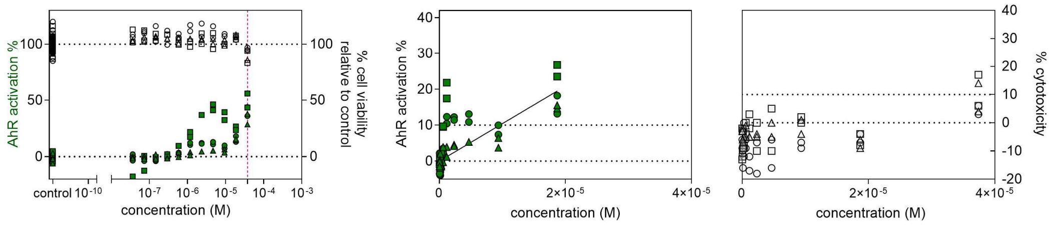

2-Methylanthracene

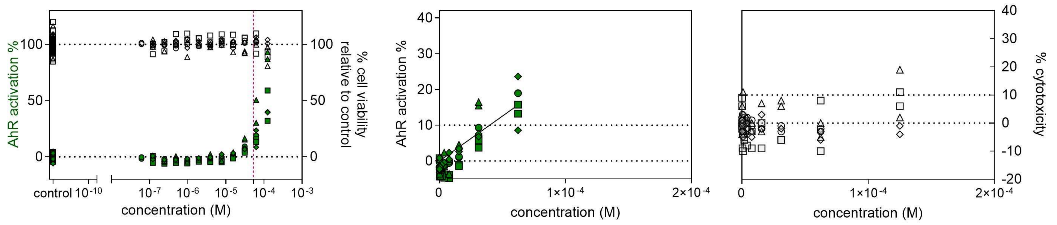

Retene

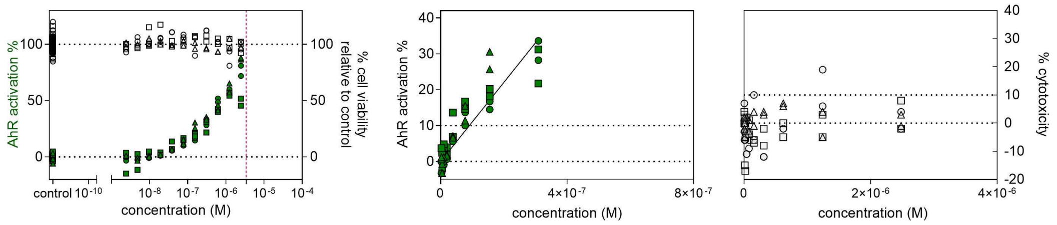

Continue

Fluoranthene

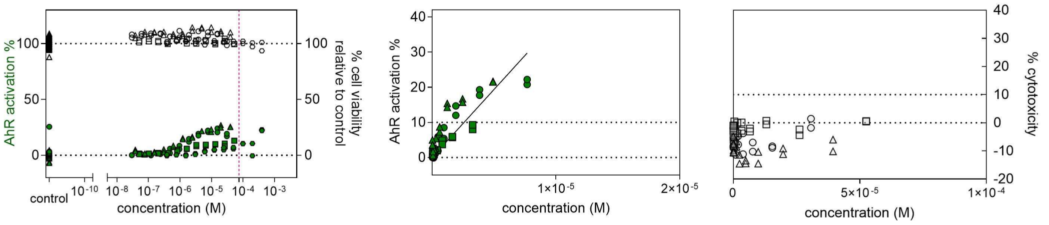

Pyrene

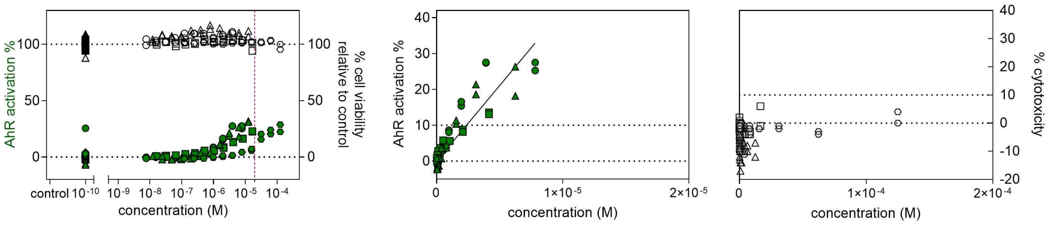

Benz(a)anthracene

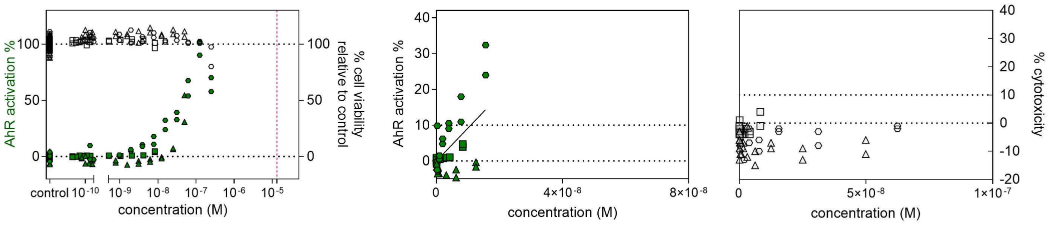

Chrysene

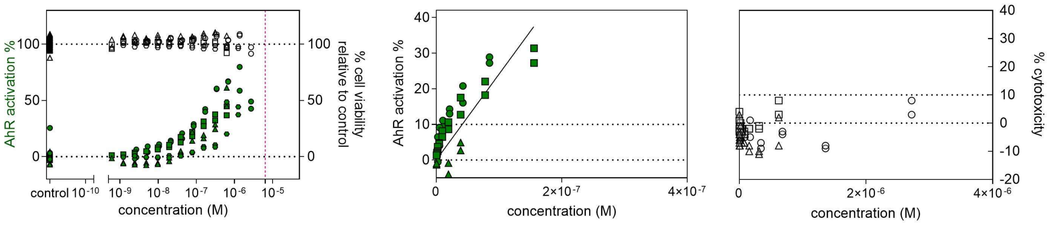

1-Methylchrysene

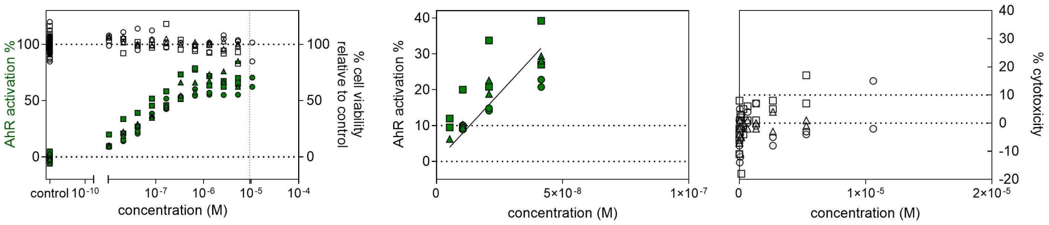

Continue

Benzo[b]fluoranthene

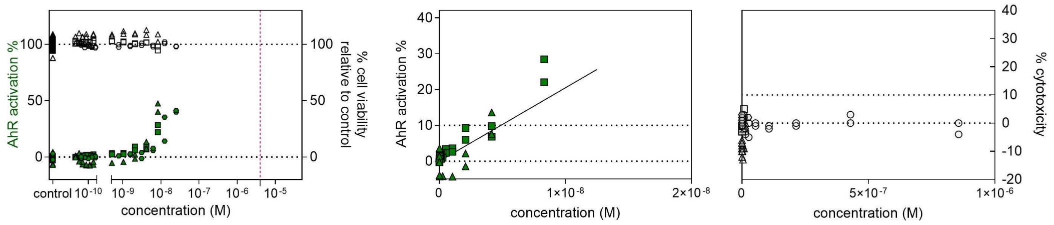

Benzo[j]fluoranthene

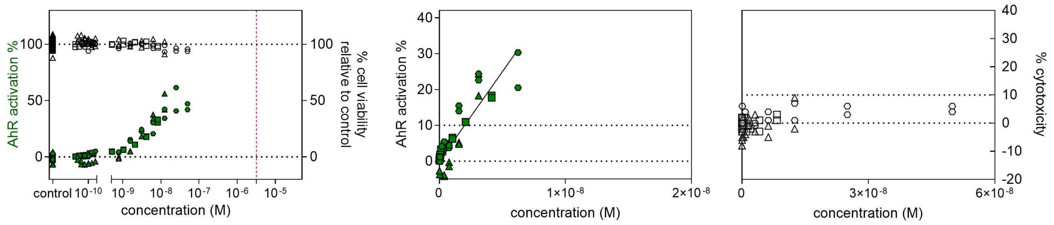

Benzo[k]fluoranthene

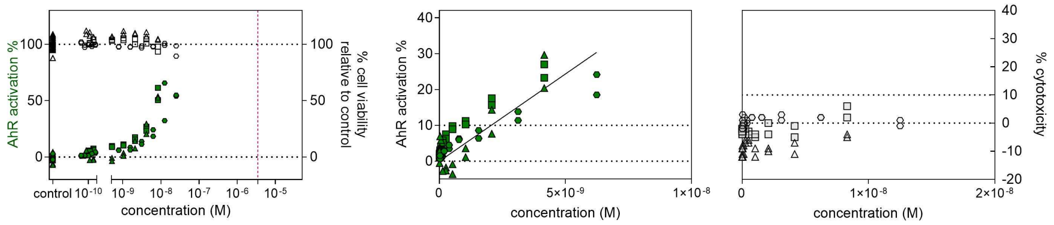

Benzo[e]pyrene

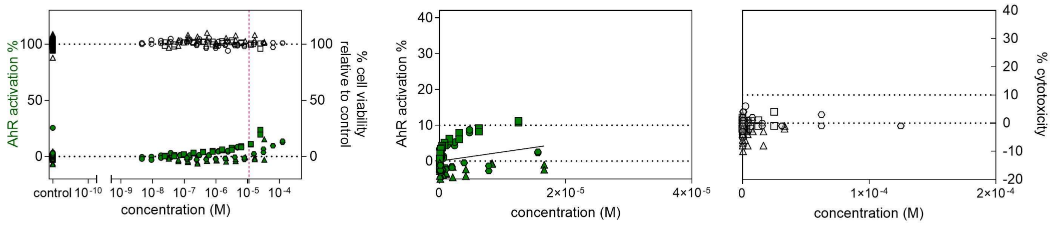

Benzo[a]pyrene

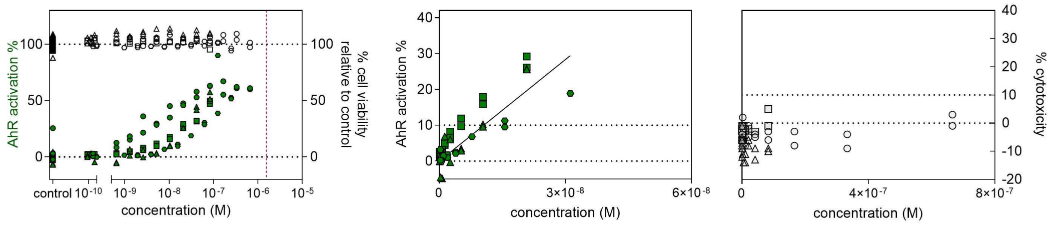

Continue

Perylene

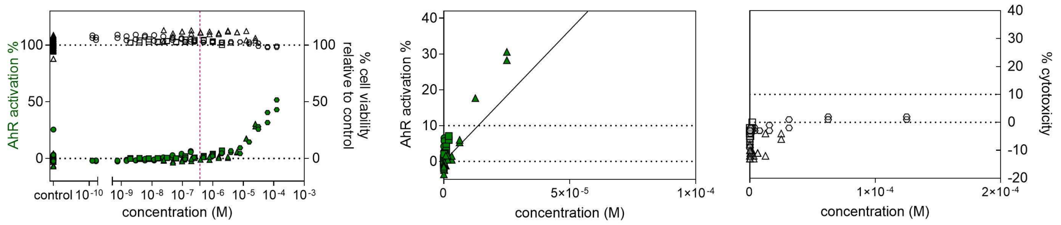

Anthanthrene

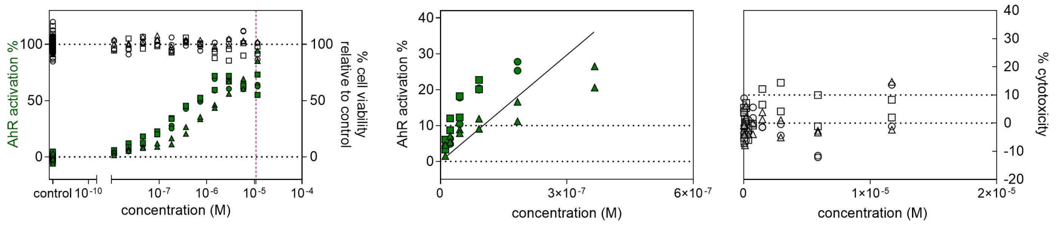

11H-Benzo[b]fluorene

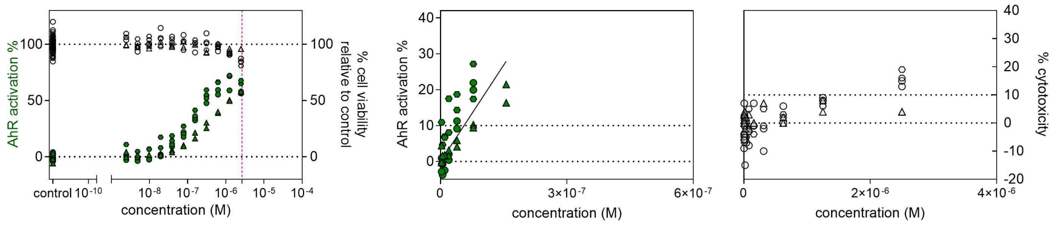

Benz[c]acridine

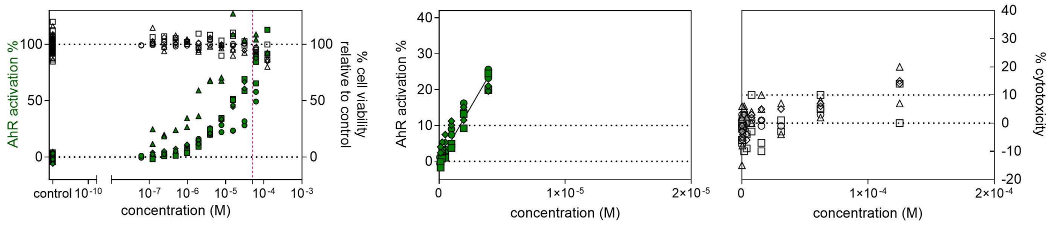

7H-Benzo(c)carbazole

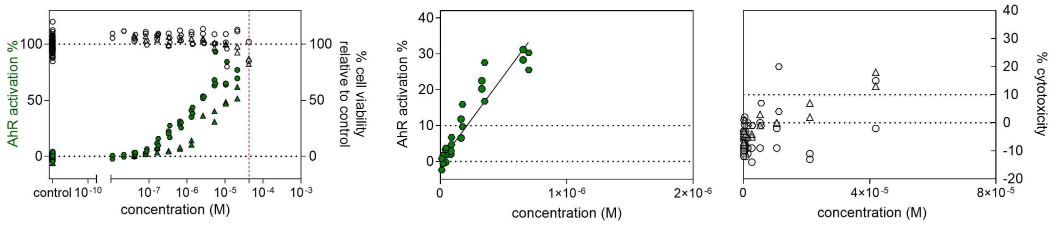

Continue

Benzo(b)naphtho(1,2-d)furan

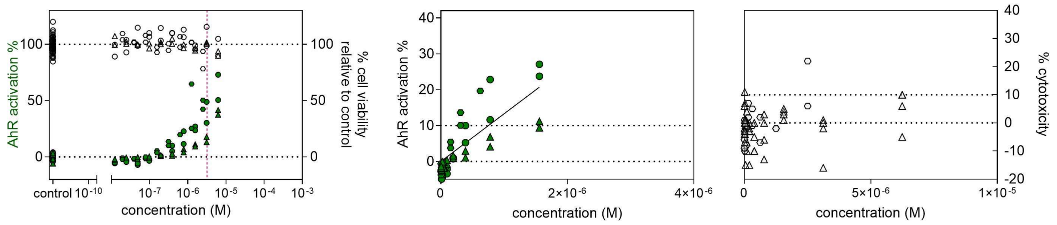

Benzo(b)naphtho(2,3-d)furan

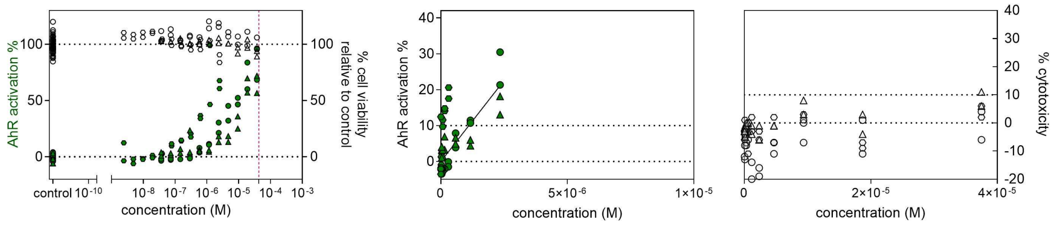

9,10-Anthracenedione

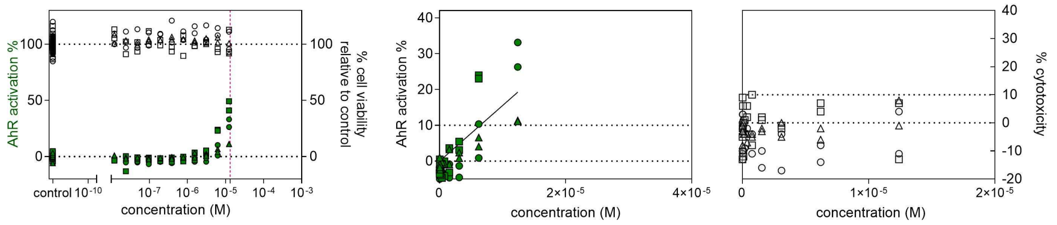

9,10-Anthracenedione, 2-methyl-

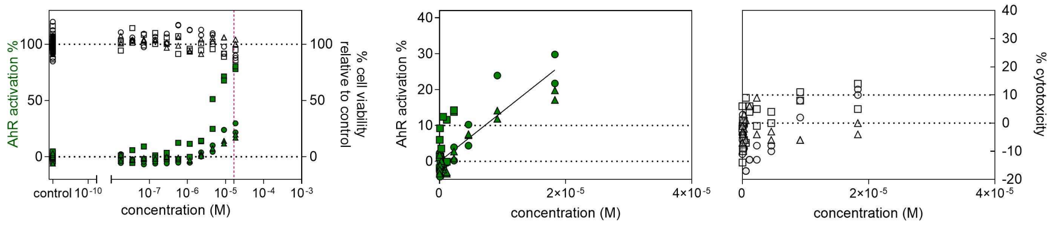

Benzo[b]naphtho[1,2-d]thiophene

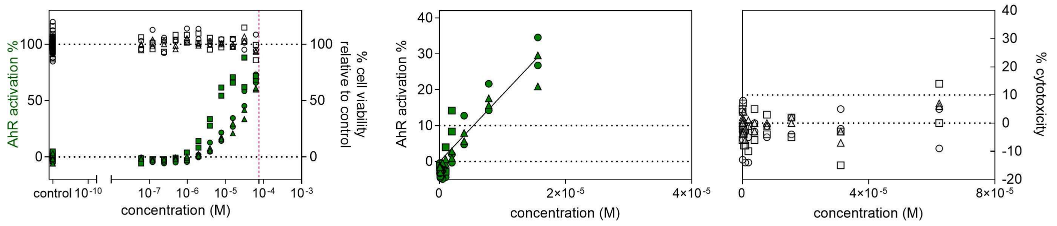

Continue

Benzo[b]naphtho[2,1-d]thiophene

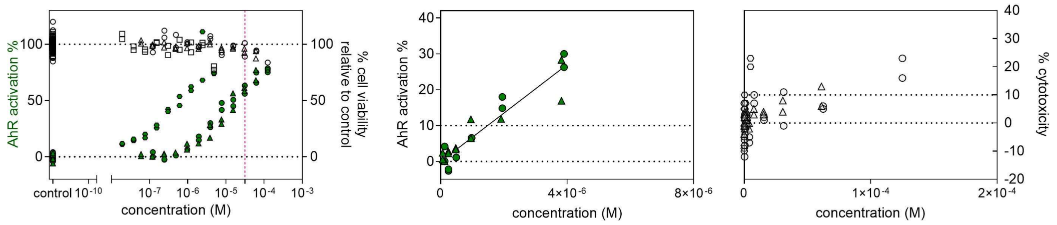

3,3',4,4',5-Pentachlorobiphenyl (PCB126)

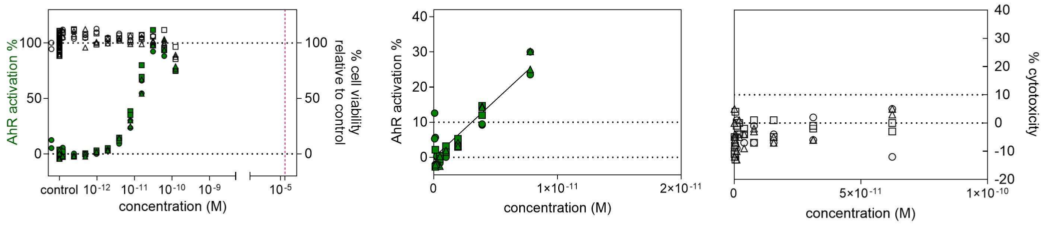

2,2',3,4,4',5'-Hexachlorobiphenyl (PCB138)

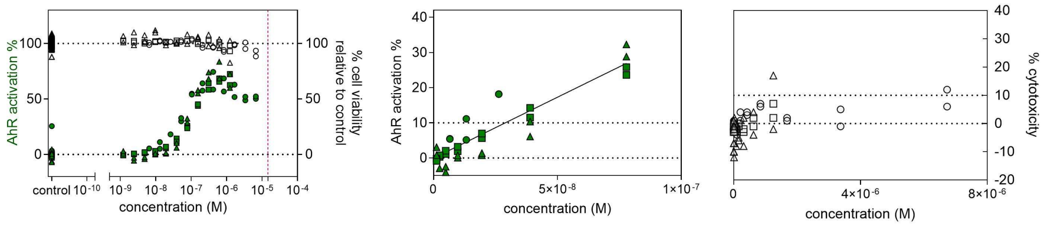

2,2',4,4',5,5'-Hexachlorobiphenyl (PCB 153)

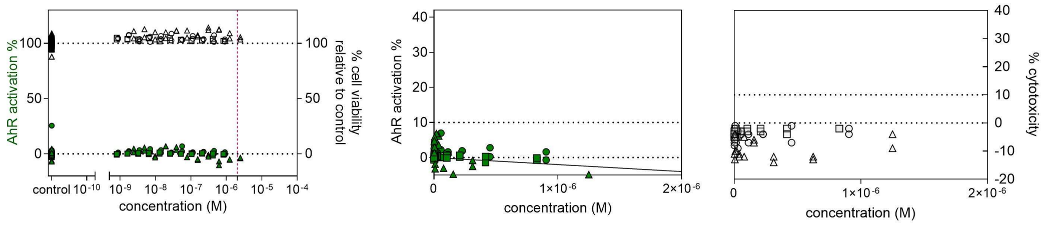

Decachlorobiphenyl (PCB 209)

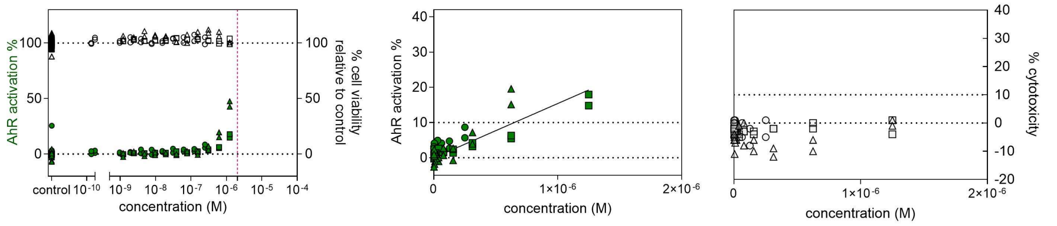

Figure S14. Concentration-response curves of the reference standards in the AhR CALUX bioassay. The calculated solubility of each tested compound in the bioassay is

indicated by a dotted line in the left panel of each plot. The solubility is provided in Table S21.

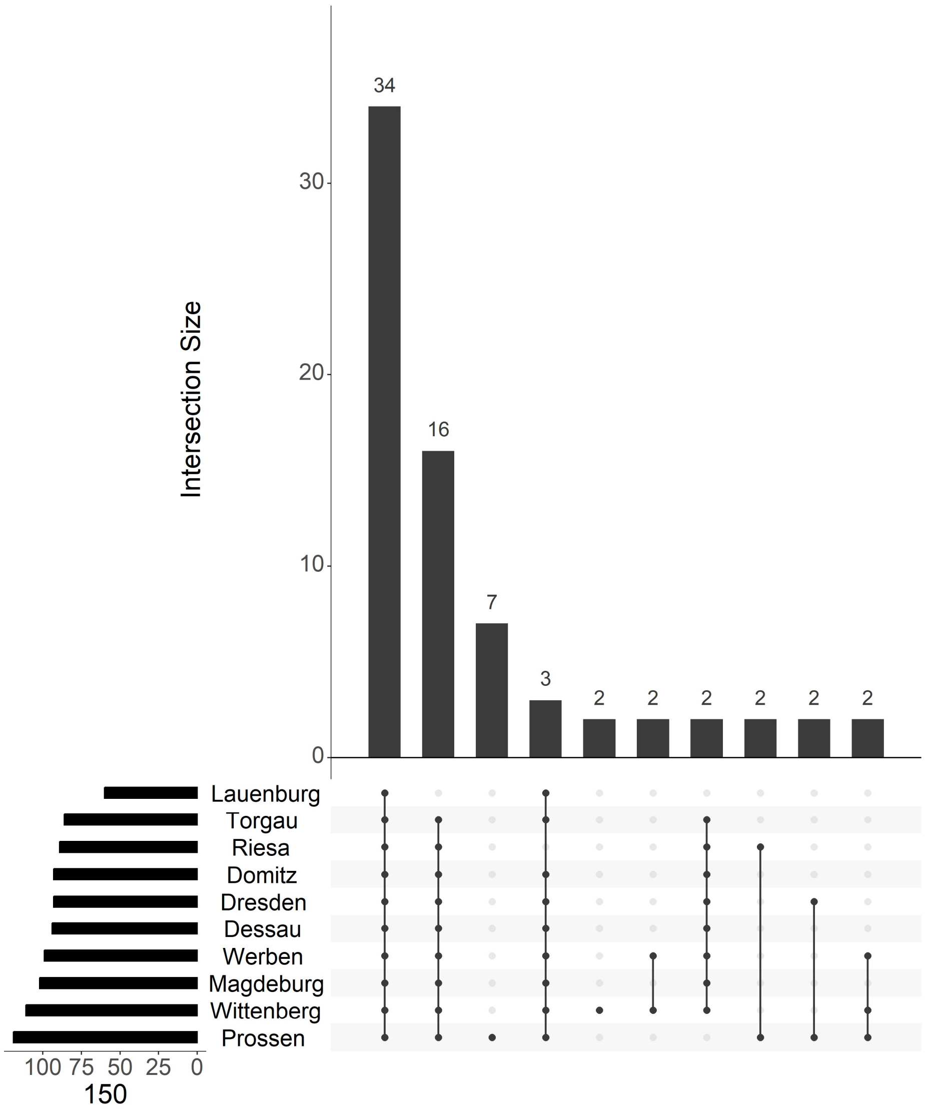

Figure S15. Intersection of detected AhR agonists in sediment samples.

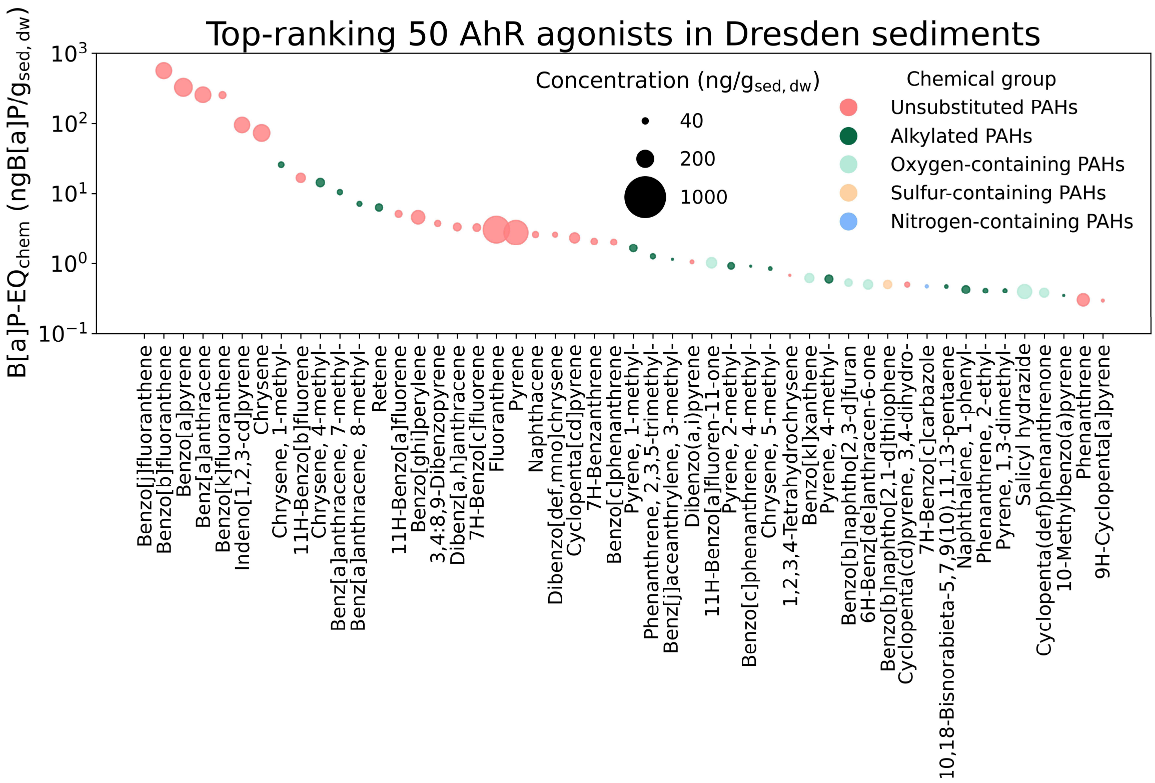
Top-ranking 50 AhR agonists in Dresden sediments
B[a]P-EQ$_{chem}$ (ngB[a]P/g$_{sed, dw}$)
10^{3}
10^{2}
10^{1}
10^{0}
10^{-1}
Concentration (ng/g$_{sed, dw}$)
40
200
1000
Chemical group
Unsubstituted PAHs
Alkylated PAHs
Oxygen-containing PAHs
Sulfur-containing PAHs
Nitrogen-containing PAHs
Benzo[j]fluoranthene
Benzo[b]fluoranthene
Benzo[a]pyrene
Benz[a]anthracene
Benzo[k]fluoranthene
Indeno[1,2,3-cd]pyrene
Chrysene
Chrysene, 1-methyl-
11H-Benzo[b]fluorene
Chrysene, 4-methyl-
Benz[a]anthracene, 7-methyl-
Benz[a]anthracene, 8-methyl-
Retene
11H-Benzo[a]fluorene
Benzo[ghi]perylene
3,4:8,9-Dibenzopyrene
Dibenz[a,h]anthracene
7H-Benzo[c]fluorene
Fluoranthene
Pyrene
Naphthacene
Dibenzo[def,mno]chrysene
Cyclopenta[cd]pyrene
7H-Benzanthrene
Benzo[c]phenanthrene
Pyrene, 1-methyl-
Phenanthrene, 2,3,5-trimethyl-
Benz[j]aceanthrylene, 3-methyl-
Dibenzo(a,i)pyrene
11H-Benzo[a]fluoren-11-one
Pyrene, 2-methyl-
Benzo[c]phenanthrene, 4-methyl-
Chrysene, 5-methyl-
1,2,3,4-Tetrahydrochrysene
Benzo[kl]xanthene
Pyrene, 4-methyl-
Benzo[b]naphtho[2,3-d]furan
6H-Benz[de]anthracen-6-one
Benzo[b]naphtho[2,1-d]thiophene
Cyclopenta(cd)pyrene, 3,4-dihydro-
7H-Benzo[c]carbazole
10,18-Bisnorabieta-5,7,9(10),11,13-pentaene
Naphthalene, 1-phenyl-
Phenanthrene, 2-ethyl-
Pyrene, 1,3-dimethyl-
Salicyl hydrazide
Cyclopenta(def)phenanthrenone
10-Methylbenzo(a)pyrene
Phenanthrene
9H-Cyclopenta[a]pyrene

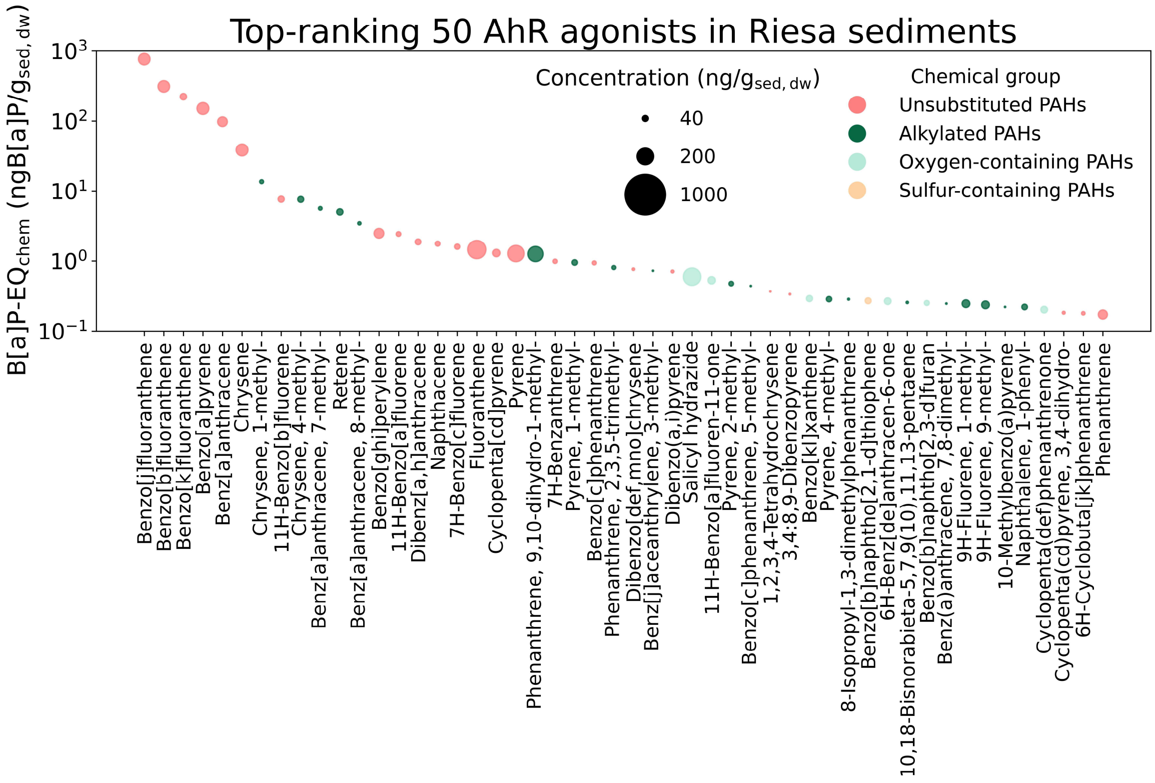
Top-ranking 50 AhR agonists in Riesa sediments
B[a]P-EQ$_{chem}$ (ngB[a]P/g$_{sed, dw}$)
10^{3}
10^{2}
10^{1}
10^{0}
10^{-1}
Concentration (ng/g$_{sed, dw}$)
40
200
1000
Chemical group
Unsubstituted PAHs
Alkylated PAHs
Oxygen-containing PAHs
Sulfur-containing PAHs
Benzo[j]fluoranthene
Benzo[b]fluoranthene
Benzo[k]fluoranthene
Benzo[a]pyrene
Benz[a]anthracene
Chrysene
Chrysene, 1-methyl-
11H-Benzo[b]fluorene
Chrysene, 4-methyl-
Benz[a]anthracene, 7-methyl-
Retene
Benz[a]anthracene, 8-methyl-
Benzo[ghi]perylene
11H-Benzo[a]fluorene
Dibenz[a,h]anthracene
Naphthacene
7H-Benzo[c]fluorene
Fluoranthene
Cyclopenta[cd]pyrene
Pyrene
Phenanthrene, 9,10-dihydro-1-methyl-
7H-Benzanthrene
Pyrene, 1-methyl-
Benzo[c]phenanthrene
Phenanthrene, 2,3,5-trimethyl-
Dibenzo[def,mno]chrysene
Benz[j]aceanthrylene, 3-methyl-
Dibenzo(a,i)pyrene
Salicyl hydrazide
11H-Benzo[a]fluoren-11-one
Pyrene, 2-methyl-
Benzo[c]phenanthrene, 5-methyl-
1,2,3,4-Tetrahydrochrysene
3,4:8,9-Dibenzopyrene
Benzo[kl]xanthene
Pyrene, 4-methyl-
8-Isopropyl-1,3-dimethylphenanthrene
Benzo[b]naphtho[2,1-d]thiophene
6H-Benz[de]anthracen-6-one
10,18-Bisnorabieta-5,7,9(10),11,13-pentaene
Benzo[b]naphtho[2,3-d]furan
Benz(a)anthracene, 7,8-dimethyl-
9H-Fluorene, 1-methyl-
9H-Fluorene, 9-methyl-
10-Methylbenzo(a)pyrene
Naphthalene, 1-phenyl-
Cyclopenta(def)phenanthrenone
Cyclopenta(cd)pyrene, 3,4-dihydro-
6H-Cyclobuta[jk]phenanthrene
Phenanthrene

Continue

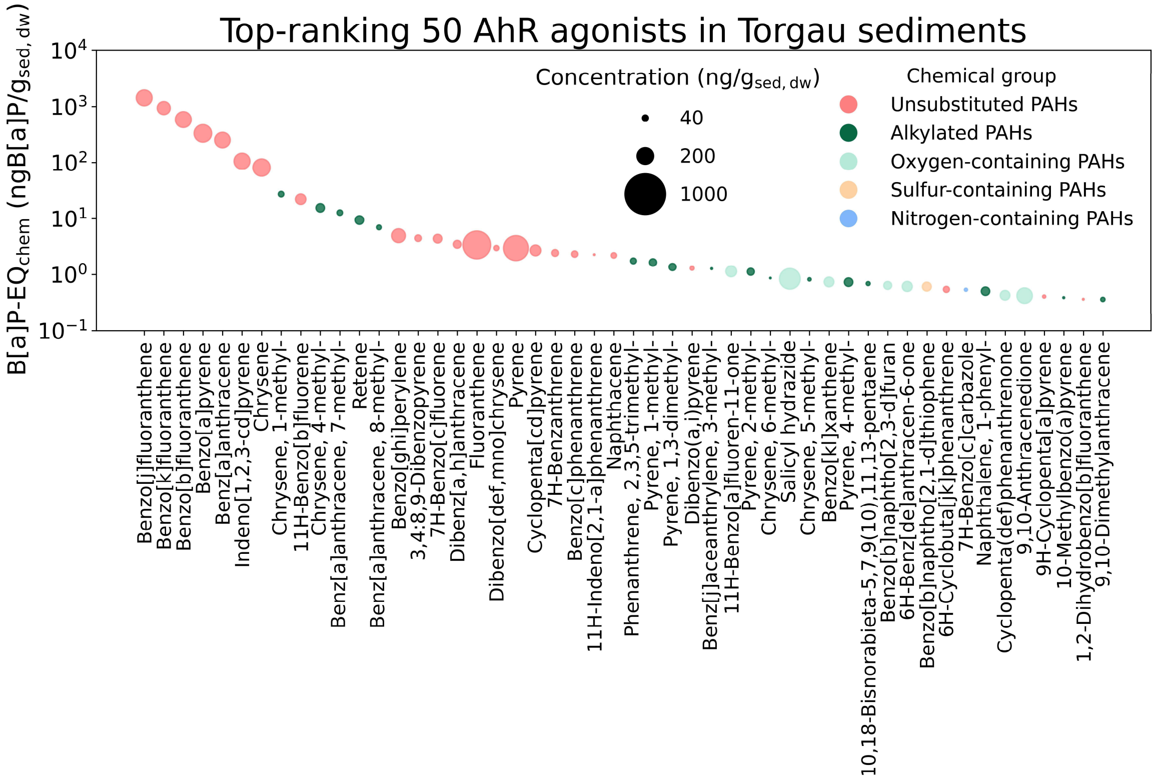
Top-ranking 50 AhR agonists in Torgau sediments
B[a]P-EQ$_{chem}$ (ngB[a]P/g$_{sed, dw}$)
10^4
10^3
10^2
10^1
10^0
10^{-1}
Concentration (ng/g$_{sed, dw}$)
40
200
1000
Chemical group
Unsubstituted PAHs
Alkylated PAHs
Oxygen-containing PAHs
Sulfur-containing PAHs
Nitrogen-containing PAHs
Benzo[j]fluoranthene
Benzo[k]fluoranthene
Benzo[b]fluoranthene
Benzo[a]pyrene
Benz[a]anthracene
Indeno[1,2,3-cd]pyrene
Chrysene
Chrysene, 1-methyl-
11H-Benzo[b]fluorene
Chrysene, 4-methyl-
Benz[a]anthracene, 7-methyl-
Retene
Benz[a]anthracene, 8-methyl-
Benzo[ghi]perylene
3,4:8,9-Dibenzopyrene
7H-Benzo[c]fluorene
Dibenz[a,h]anthracene
Fluoranthene
Dibenzo[def,mno]chrysene
Pyrene
Cyclopenta[cd]pyrene
7H-Benzanthrene
Benzo[c]phenanthrene
11H-Indeno[2,1-a]phenanthrene
Naphthacene
Phenanthrene, 2,3,5-trimethyl-
Pyrene, 1-methyl-
Pyrene, 1,3-dimethyl-
Dibenzo(a,i)pyrene
Benz[j]aceanthrylene, 3-methyl-
11H-Benzo[a]fluoren-11-one
Pyrene, 2-methyl-
Chrysene, 6-methyl-
Salicyl hydrazide
Chrysene, 5-methyl-
Benzo[kl]xanthene
Pyrene, 4-methyl-
10,18-Bisnorabieta-5,7,9(10),11,13-pentaene
Benzo[b]naphtho[2,3-d]furan
6H-Benz[de]anthracen-6-one
Benzo[b]naphtho[2,1-d]thiophene
6H-Cyclobuta[jk]phenanthrene
7H-Benzo[c]carbazole
Naphthalene, 1-phenyl-
Cyclopenta(def)phenanthrenone
9,10-Anthracenedione
9H-Cyclopenta[a]pyrene
10-Methylbenzo(a)pyrene
1,2-Dihydrobenzo[b]fluoranthene
9,10-Dimethylanthracene

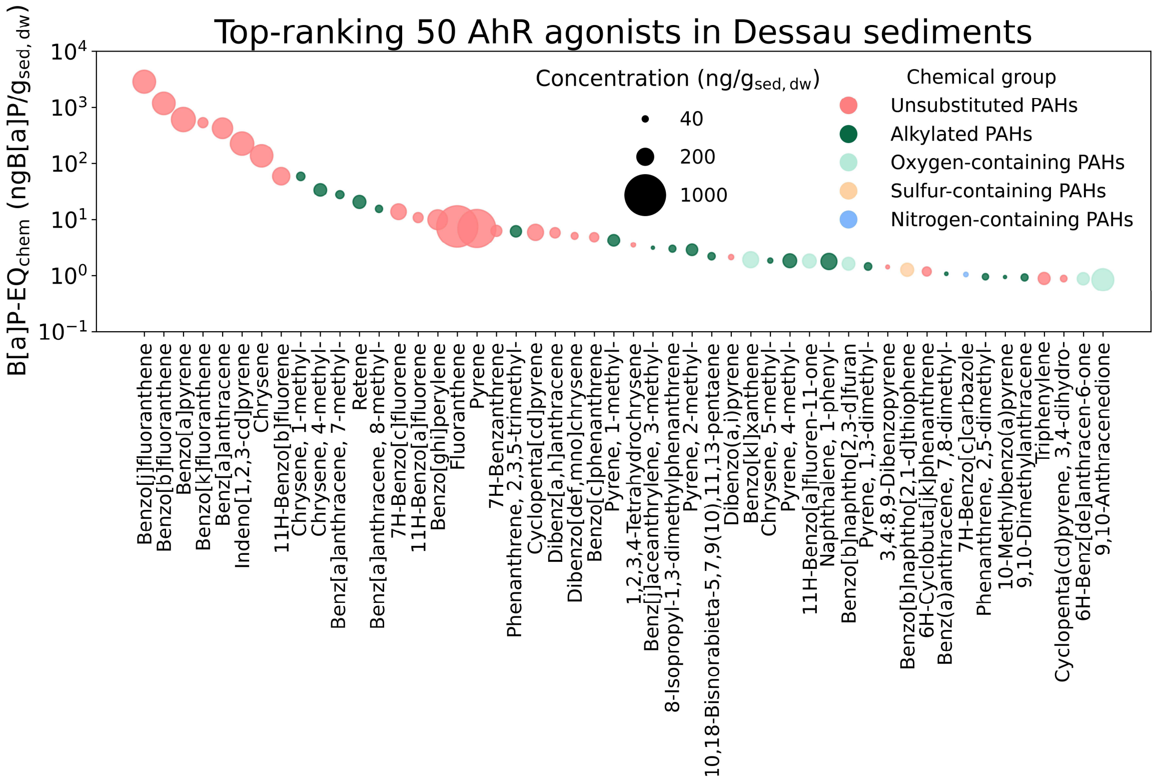
Top-ranking 50 AhR agonists in Dessau sediments
B[a]P-EQ$_{chem}$ (ngB[a]P/g$_{sed, dw}$)
10^4
10^3
10^2
10^1
10^0
10^{-1}
Concentration (ng/g$_{sed, dw}$)
40
200
1000
Chemical group
Unsubstituted PAHs
Alkylated PAHs
Oxygen-containing PAHs
Sulfur-containing PAHs
Nitrogen-containing PAHs
Benzo[j]fluoranthene
Benzo[b]fluoranthene
Benzo[a]pyrene
Benzo[k]fluoranthene
Benz[a]anthracene
Indeno[1,2,3-cd]pyrene
Chrysene
11H-Benzo[b]fluorene
Chrysene, 1-methyl-
Chrysene, 4-methyl-
Benz[a]anthracene, 7-methyl-
Retene
Benz[a]anthracene, 8-methyl-
7H-Benzo[c]fluorene
11H-Benzo[a]fluorene
Benzo[ghi]perylene
Fluoranthene
Pyrene
7H-Benzanthrene
Phenanthrene, 2,3,5-trimethyl-
Cyclopenta[cd]pyrene
Dibenz[a,h]anthracene
Dibenzo[def,mno]chrysene
Benzo[c]phenanthrene
Pyrene, 1-methyl-
1,2,3,4-Tetrahydrochrysene
Benz[j]aceanthrylene, 3-methyl-
8-Isopropyl-1,3-dimethylphenanthrene
Pyrene, 2-methyl-
10,18-Bisnorabieta-5,7,9(10),11,13-pentaene
Dibenzo(a,i)pyrene
Benzo[kl]xanthene
Chrysene, 5-methyl-
Pyrene, 4-methyl-
11H-Benzo[a]fluoren-11-one
Naphthalene, 1-phenyl-
Benzo[b]naphtho[2,3-d]furan
Pyrene, 1,3-dimethyl-
3,4:8,9-Dibenzopyrene
Benzo[b]naphtho[2,1-d]thiophene
6H-Cyclobuta[jk]phenanthrene
Benz(a)anthracene, 7,8-dimethyl-
7H-Benzo[c]carbazole
Phenanthrene, 2,5-dimethyl-
10-Methylbenzo(a)pyrene
9,10-Dimethylanthracene
Triphenylene
Cyclopenta(cd)pyrene, 3,4-dihydro-
6H-Benz[de]anthracen-6-one
9,10-Anthracenedione

Continue

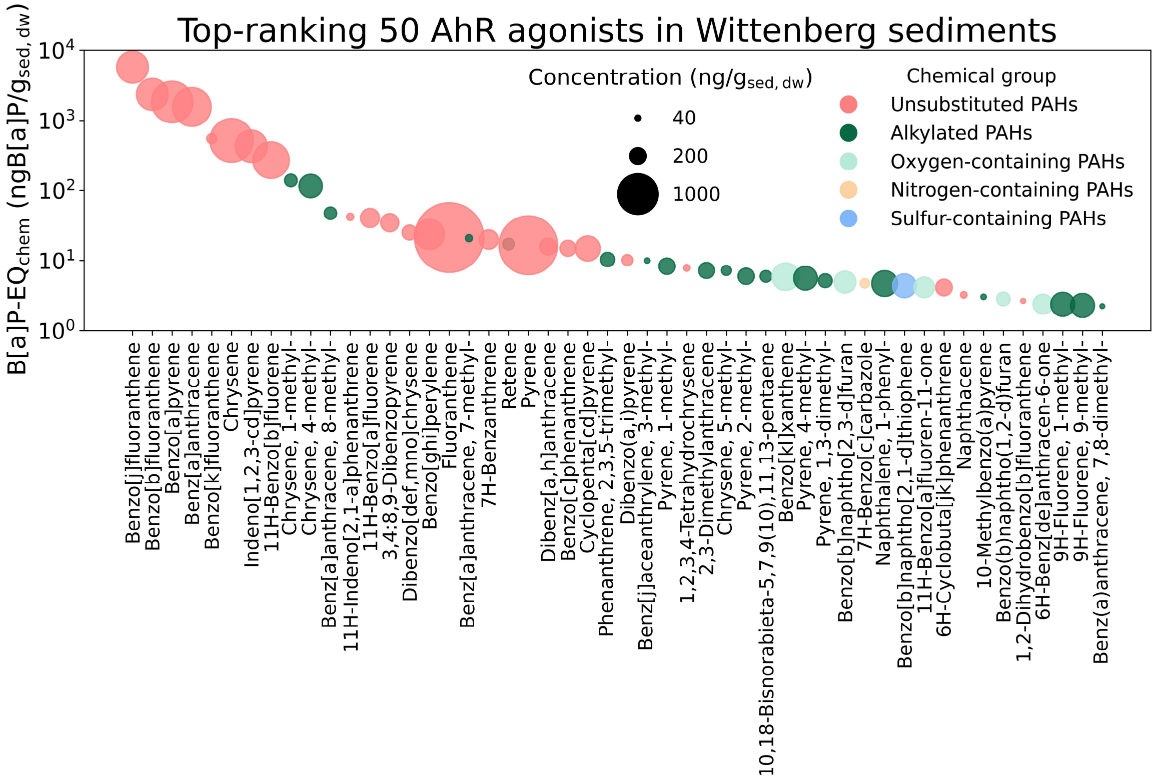
Top-ranking 50 AhR agonists in Wittenberg sediments
B[a]P-EQchem (ngB[a]P/gsed, dw)
10^4
10^3
10^2
10^1
10^0
Concentration (ng/gsed, dw)
40
200
1000
Chemical group
Unsubstituted PAHs
Alkylated PAHs
Oxygen-containing PAHs
Nitrogen-containing PAHs
Sulfur-containing PAHs
Benzo[j]fluoranthene
Benzo[b]fluoranthene
Benzo[a]pyrene
Benz[a]anthracene
Benzo[k]fluoranthene
Chrysene
Indeno[1,2,3-cd]pyrene
11H-Benzo[b]fluorene
Chrysene, 1-methyl-
Chrysene, 4-methyl-
Benz[a]anthracene, 8-methyl-
11H-Indeno[2,1-a]phenanthrene
11H-Benzo[a]fluorene
3,4:8,9-Dibenzopyrene
Dibenzo[def,mno]chrysene
Benzo[ghi]perylene
Fluoranthene
Benz[a]anthracene, 7-methyl-
7H-Benzanthrene
Retene
Pyrene
Dibenz[a,h]anthracene
Benzo[c]phenanthrene
Cyclopenta[cd]pyrene
Phenanthrene, 2,3,5-trimethyl-
Dibenzo(a,i)pyrene
Benz[j]aceanthrylene, 3-methyl-
Pyrene, 1-methyl-
1,2,3,4-Tetrahydrochrysene
2,3-Dimethylanthracene
Chrysene, 5-methyl-
Pyrene, 2-methyl-
10,18-Bisnorabieta-5,7,9(10),11,13-pentaene
Benzo[kl]xanthene
Pyrene, 4-methyl-
Pyrene, 1,3-dimethyl-
Benzo[b]naphtho[2,3-d]furan
7H-Benzo[c]carbazole
Naphthalene, 1-phenyl-
Benzo[b]naphtho[2,1-d]thiophene
11H-Benzo[a]fluoren-11-one
6H-Cyclobuta[jk]phenanthrene
Naphthacene
10-Methylbenzo(a)pyrene
Benzo(b)naphtho(1,2-d)furan
1,2-Dihydrobenzo[b]fluoranthene
6H-Benz[de]anthracen-6-one
9H-Fluorene, 1-methyl-
9H-Fluorene, 9-methyl-
Benz(a)anthracene, 7,8-dimethyl-

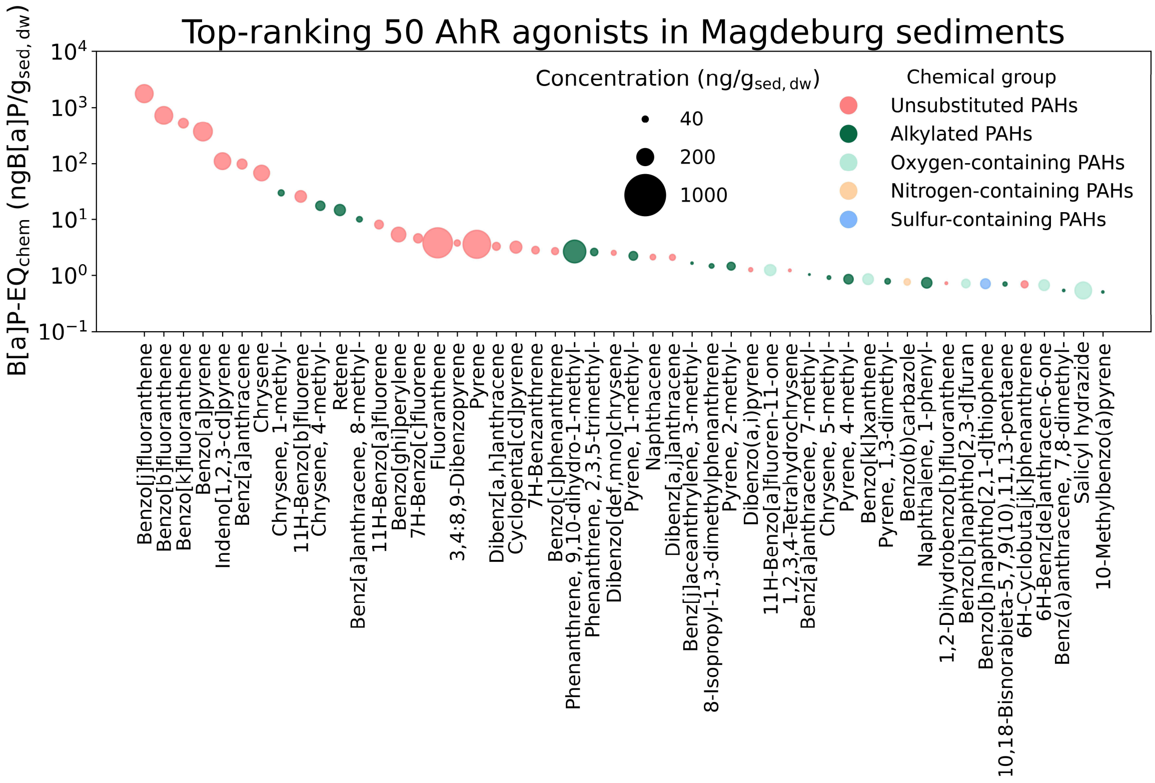
Top-ranking 50 AhR agonists in Magdeburg sediments
B[a]P-EQchem (ngB[a]P/gsed, dw)
10^4
10^3
10^2
10^1
10^0
10^−1
Concentration (ng/gsed, dw)
40
200
1000
Chemical group
Unsubstituted PAHs
Alkylated PAHs
Oxygen-containing PAHs
Nitrogen-containing PAHs
Sulfur-containing PAHs
Benzo[j]fluoranthene
Benzo[b]fluoranthene
Benzo[k]fluoranthene
Benzo[a]pyrene
Indeno[1,2,3-cd]pyrene
Benz[a]anthracene
Chrysene
Chrysene, 1-methyl-
11H-Benzo[b]fluorene
Chrysene, 4-methyl-
Retene
Benz[a]anthracene, 8-methyl-
11H-Benzo[a]fluorene
Benzo[ghi]perylene
7H-Benzo[c]fluorene
Fluoranthene
3,4:8,9-Dibenzopyrene
Pyrene
Dibenz[a,h]anthracene
Cyclopenta[cd]pyrene
7H-Benzanthrene
Benzo[c]phenanthrene
Phenanthrene, 9,10-dihydro-1-methyl-
Phenanthrene, 2,3,5-trimethyl-
Dibenzo[def,mno]chrysene
Pyrene, 1-methyl-
Naphthacene
Dibenz[a,j]anthracene
Benz[j]aceanthrylene, 3-methyl-
8-Isopropyl-1,3-dimethylphenanthrene
Pyrene, 2-methyl-
Dibenzo(a,i)pyrene
11H-Benzo[a]fluoren-11-one
1,2,3,4-Tetrahydrochrysene
Benz[a]anthracene, 7-methyl-
Chrysene, 5-methyl-
Pyrene, 4-methyl-
Benzo[kl]xanthene
Pyrene, 1,3-dimethyl-
Benzo(b)carbazole
Naphthalene, 1-phenyl-
1,2-Dihydrobenzo[b]fluoranthene
Benzo[b]naphtho[2,3-d]furan
Benzo[b]naphtho[2,1-d]thiophene
10,18-Bisnorabieta-5,7,9(10),11,13-pentaene
6H-Cyclobuta[jk]phenanthrene
6H-Benz[de]anthracen-6-one
Benz(a)anthracene, 7,8-dimethyl-
Salicyl hydrazide
10-Methylbenzo(a)pyrene

Continue

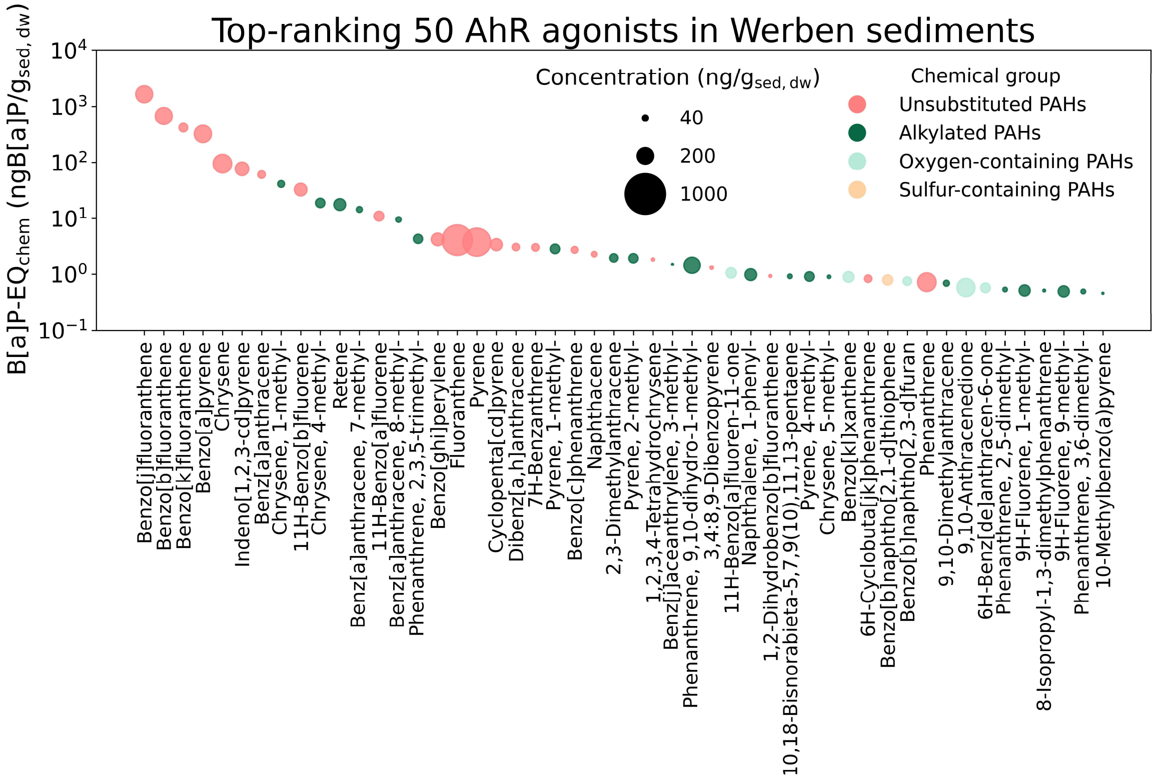
Top-ranking 50 AhR agonists in Werben sediments
B[a]P-EQchem (ngB[a]P/gsed, dw)
Concentration (ng/gsed, dw)
40
200
1000
Chemical group
Unsubstituted PAHs
Alkylated PAHs
Oxygen-containing PAHs
Sulfur-containing PAHs
Benzo[j]fluoranthene
Benzo[b]fluoranthene
Benzo[k]fluoranthene
Benzo[a]pyrene
Chrysene
Indeno[1,2,3-cd]pyrene
Benz[a]anthracene
Chrysene, 1-methyl-
11H-Benzo[b]fluorene
Chrysene, 4-methyl-
Retene
Benz[a]anthracene, 7-methyl-
11H-Benzo[a]fluorene
Benz[a]anthracene, 8-methyl-
Phenanthrene, 2,3,5-trimethyl-
Benzo[ghi]perylene
Fluoranthene
Pyrene
Cyclopenta[cd]pyrene
Dibenz[a,h]anthracene
7H-Benzanthrene
Pyrene, 1-methyl-
Benzo[c]phenanthrene
Naphthacene
2,3-Dimethylanthracene
Pyrene, 2-methyl-
1,2,3,4-Tetrahydrochrysene
Benz[j]aceanthrylene, 3-methyl-
Phenanthrene, 9,10-dihydro-1-methyl-
3,4:8,9-Dibenzopyrene
11H-Benzo[a]fluoren-11-one
Naphthalene, 1-phenyl-
1,2-Dihydrodibenzo[b]fluoranthene
10,18-Bisnorabieta-5,7,9(10),11,13-pentaene
Pyrene, 4-methyl-
Chrysene, 5-methyl-
Benzo[kl]xanthene
6H-Cyclobuta[jk]phenanthrene
Benzo[b]naphtho[2,1-d]thiophene
Benzo[b]naphtho[2,3-d]furan
Phenanthrene
9,10-Dimethylanthracene
9,10-Anthracenedione
6H-Benz[de]anthracen-6-one
Phenanthrene, 2,5-dimethyl-
9H-Fluorene, 1-methyl-
8-Isopropyl-1,3-dimethylphenanthrene
9H-Fluorene, 9-methyl-
Phenanthrene, 3,6-dimethyl-
10-Methylbenzo(a)pyrene

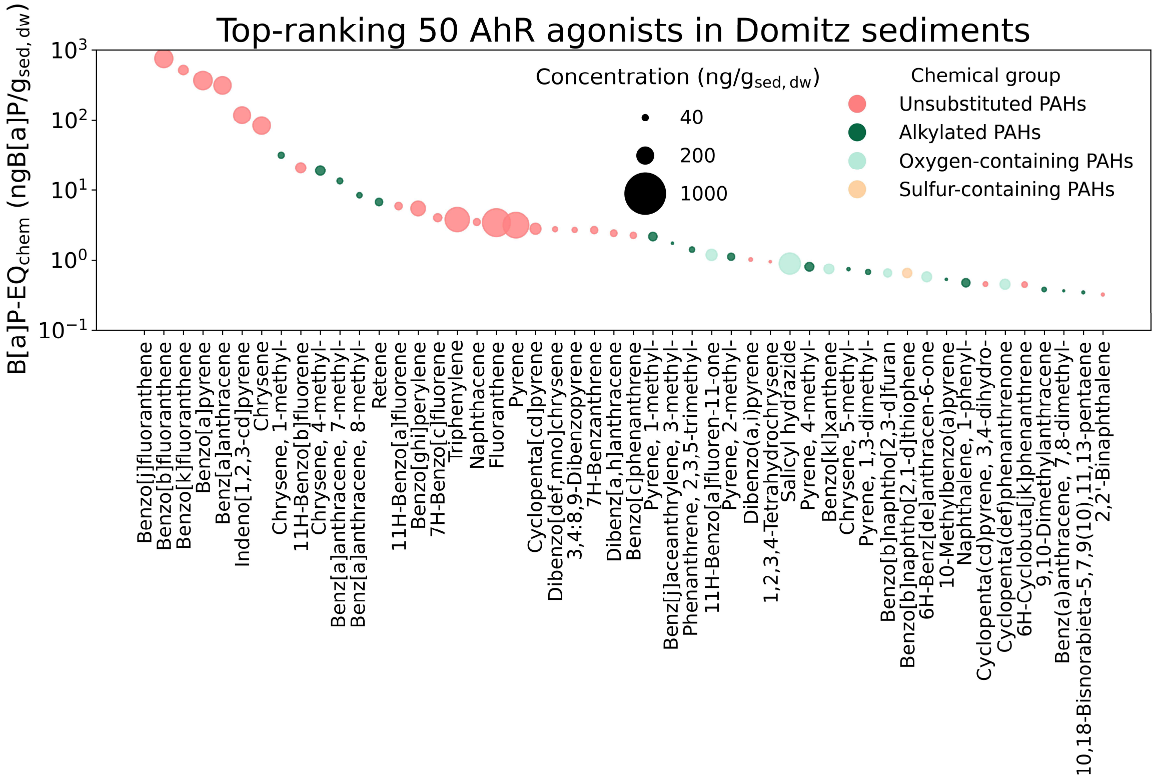
Top-ranking 50 AhR agonists in Domitz sediments
B[a]P-EQchem (ngB[a]P/gsed, dw)
Concentration (ng/gsed, dw)
40
200
1000
Chemical group
Unsubstituted PAHs
Alkylated PAHs
Oxygen-containing PAHs
Sulfur-containing PAHs
Benzo[j]fluoranthene
Benzo[b]fluoranthene
Benzo[k]fluoranthene
Benzo[a]pyrene
Benz[a]anthracene
Indeno[1,2,3-cd]pyrene
Chrysene
Chrysene, 1-methyl-
11H-Benzo[b]fluorene
Chrysene, 4-methyl-
Benz[a]anthracene, 7-methyl-
Benz[a]anthracene, 8-methyl-
Retene
11H-Benzo[a]fluorene
Benzo[ghi]perylene
7H-Benzo[c]fluorene
Triphenylene
Naphthacene
Fluoranthene
Pyrene
Cyclopenta[cd]pyrene
Dibenzo[def,mno]chrysene
3,4:8,9-Dibenzopyrene
7H-Benzanthrene
Dibenz[a,h]anthracene
Benzo[c]phenanthrene
Pyrene, 1-methyl-
Benz[j]aceanthrylene, 3-methyl-
Phenanthrene, 2,3,5-trimethyl-
11H-Benzo[a]fluoren-11-one
Pyrene, 2-methyl-
Dibenzo(a,i)pyrene
1,2,3,4-Tetrahydrochrysene
Salicyl hydrazide
Pyrene, 4-methyl-
Benzo[kl]xanthene
Chrysene, 5-methyl-
Pyrene, 1,3-dimethyl-
Benzo[b]naphtho[2,3-d]furan
Benzo[b]naphtho[2,1-d]thiophene
6H-Benz[de]anthracen-6-one
10-Methylbenzo(a)pyrene
Naphthalene, 1-phenyl-
Cyclopenta(cd)pyrene, 3,4-dihydro-
Cyclopenta(def)phenanthrenone
6H-Cyclobuta[jk]phenanthrene
9,10-Dimethylanthracene
Benz(a)anthracene, 7,8-dimethyl-
10,18-Bisnorabieta-5,7,9(10),11,13-pentaene
2,2'-Binaphthalene

Continue

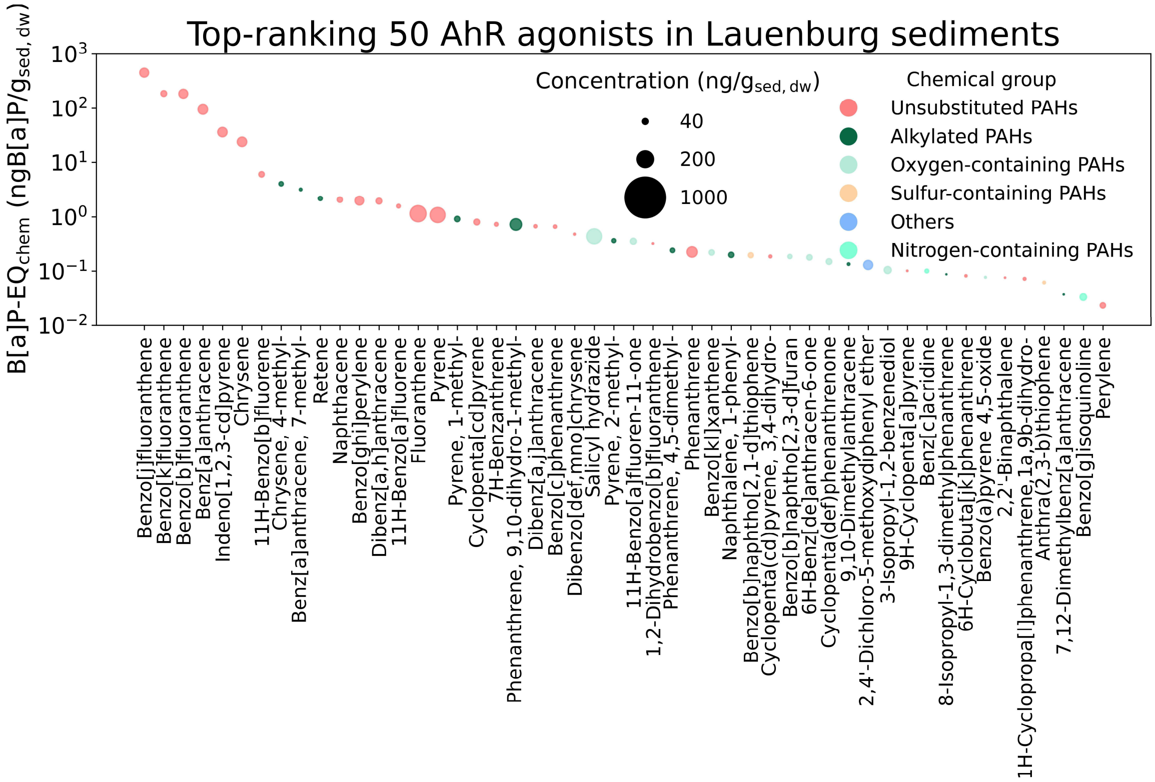

Figure S16. Prioritization of the top 50 identified AhR agonists based on their bioanalytical equivalent concentrations relative to benzo[a]pyrene (B[a]P-EQ) in each sediment extract.

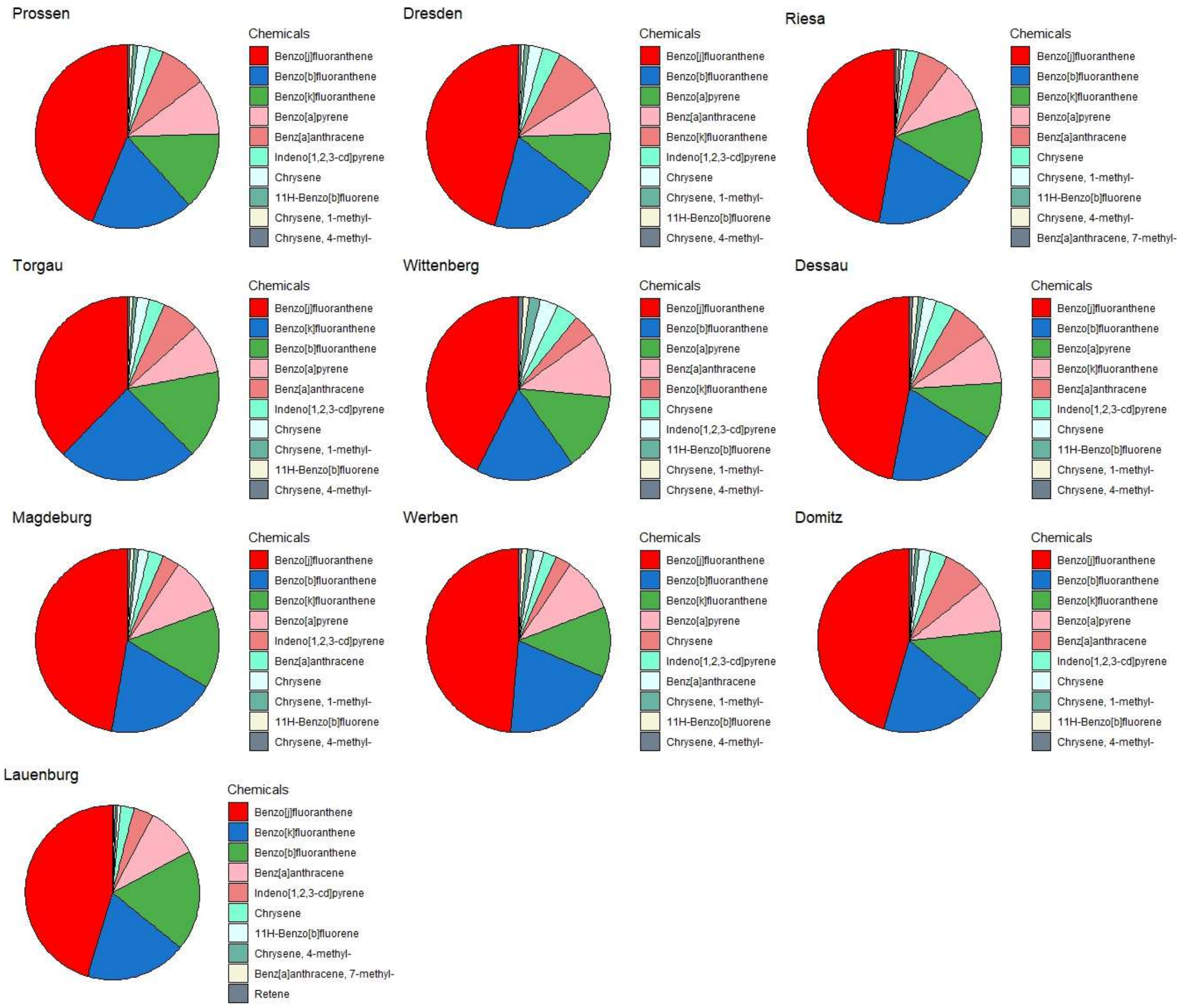

Figure S17. Relative contribution of the top 10 identified AhR agonists to the cumulative B[a]P-EQ in each sediment sample.
